# MicroRNA profiling of diabetic atherosclerosis in a rat model

**DOI:** 10.1186/s40001-018-0354-5

**Published:** 2018-11-03

**Authors:** Yuejin Li, Le Xiao, Jinyuan Li, Ping Sun, Lei Shang, Jian Zhang, Quan Zhao, Yiming Ouyang, Linhai Li, Kunmei Gong

**Affiliations:** 1 The First Department of General Surgery, The First People’s Hospital of Yunnan Province, 157 JinBi Road, Kunming, 650032 Yunnan People’s Republic of China; 2 Medical Faculty, Kunming University of Science and Technology, Kunming, Yunnan China

**Keywords:** Diabetic atherosclerosis, Differentially expressed miRNA, miRNA-gene regulatory network

## Abstract

**Objective:**

The incidence of diabetic atherosclerosis (DA) is increasing worldwide. The study aim was to identify differentially expressed microRNAs (DE-miRs) potentially associated with the initiation and/or progression of DA, thereby yielding new insights into this disease.

**Methods:**

Matched iliac artery tissue samples were isolated from 6 male rats with or without DA. The Affymetrix GeneChip microRNA 4.0 Array was used to detect miRs. Differential expression between atherosclerotic group and non-atherosclerotic group samples was analyzed using the Gene-Cloud of Biotechnology Information platform. Targetscan and miRanda were then used to predict targets of DE-miRs. Functions and pathways were identified for significantly enriched candidate target genes and a DE-miR functional regulatory network was assembled to identify DA-associated core target genes.

**Results:**

A total of nine DE-miRs (rno-miR-206-3p, rno-miR-133a-5p, rno-miR-133b-3p, rno-miR-133a-3p, rno-miR-325-5p, rno-miR-675-3p, rno-miR-411-5p, rno-miR-329-3p, and rno-miR-126a-3p) were identified, all of which were up-regulated and together predicted to target 3349 genes. The target genes were enriched in known functions and pathways related to lipid and glucose metabolism. The functional regulatory network indicated a modulatory pattern of these metabolic functions with DE-miRs. The miR-gene network suggested arpp19 and MDM4 as possible DA-related core target genes.

**Conclusion:**

The present study identified DE-miRs and miRNA-gene networks enriched for lipid and glucose metabolic functions and pathways, and arpp19 and MDM4 as potential DA-related core target genes, suggesting DE-miRs and/or arpp19 and MDM4 could act as potential diagnostic markers or therapeutic targets for DA.

**Electronic supplementary material:**

The online version of this article (10.1186/s40001-018-0354-5) contains supplementary material, which is available to authorized users.

## Introduction

The increasing global prevalence of type-2 diabetes mellitus (T2DM) is a major threat to human health. T2DM leads to vascular disease affecting nearly all blood vessel types. It is associated with a markedly increased incidence of cardiovascular disease (CVD) [1–3] including atherosclerosis (AS) affecting the macrovasculature [4, 5]. Indeed, DM induced atherosclerosis (DA) is a major cause of most of the morbidity, hospitalizations and deaths that occur among diabetes patients [6, 7]. However, not all DM patients develop DA over the course of a lifetime [8, 9] and identification of molecular markers distinguishing DA susceptibility among DM patients is an important goal.

In the last decade, microRNAs (miRNAs), conserved 19–25 nucleotide non-coding RNAs that post-translationally regulate gene expression have become a focus of translational research [10–13]. miRNAs are known to be involved in the onset and development of several disorders including diabetes and associated complications and regulate biological pathways implicated in DA [11, 14]. miRNAs or their regulated target genes could therefore prove useful as biomarkers to predict the onset, progression, and severity of diseases such as atherosclerosis associated with diabetic hyperglycemia [15–18]. The present study was designed to yield new insights into miRNAs associated with DA and identify potential molecular targets for novel diagnostics and therapeutic approaches.

## Materials and methods

### Establishment of streptozotocin-induced diabetic rat models

Male SD rats (4–6 weeks old, 280–310 g in weight) were obtained from Kunming Medical University. Rats were maintained under conditions of controlled temperature (22 °C ± 1 °C) and humidity (60%) with a 12-h light/dark cycle and with free access to food and water. All experimental procedures were approved by the Kunming Medical University Committee of Ethical Conduct in the Use of Animals in Experiments (2014YYGJ116) in accordance with the International Guiding Principles for Biomedical Research Involving Animals [19].

Animals were fed a high-fat, high glucose, high cholesterol diet (20% sucrose, 10% animal oil, 1.0% bile salt and 2.5% cholesterol; Boai Gang Trading Company, Beijing). After 2 weeks, rats received a single 30 mg/kg intraperitoneal injection of streptozotocin (STZ; Sigma, St. Louis, MO) in 10 mM sodium citrate buffer, pH 4.5 and were thereafter maintained on the same high-fat, high glucose, high cholesterol diet for the next 4 weeks. At the end of that time, blood glucose levels were determined with a Roche glucose meter. Diabetic rats were selected based upon fasting blood glucose > 6.9 mmol/L, random blood glucose > 16.7 mmol/L; with the same result obtained 3 days later. The diabetic rats continued on the high-fat, high glucose, high cholesterol diet with weekly monitoring of weight and random blood glucose levels.

Ultrasound Doppler detection was used to diagnose DA in diabetic rats during the 10th week after STZ administration. Based on the ultrasound results, we randomly chose three diabetic rats with atherosclerosis (atherosclerotic group, AG) and three diabetic rats without atherosclerosis (non-atherosclerotic group, NAG) for sample collection.

Iliac aorta tissue samples were removed from each of three AG/NAG randomly matched pairs of diabetic rats, then immediately stored at − 80 °C for subsequent microarray analysis. Tissue samples were taken from the arterial intima and inner media layer to ensure that miRNA profiles represent the diseased artery itself rather than merely the intimal plaque.

### Microarray profiling of miRNAs

Total RNA was extracted from tissue samples using the microRNAeasy Mini Kit (Qiagen) according to the manufacturer’s instructions. Affymetrix GeneChip microRNA 4.0 Array was used to analyze miRNA expression in the tissue samples. MicroRNA probe sequences were obtained from the Sanger miRBase 20.0 database (http://microrna.sanger.ac.uk/sequences/), in which the amount of rat miRNA adds up to 728. Candidate RNA expression in the six tissue samples was analyzed using qRT-PCR, and results were normalized to U6 expression.

### Differentially expressed gene (DE-miR) analysis

miRNA expression data from AG to NAG tissue samples were analyzed using the Gene-Cloud of Biotechnology Information (GCBI) platform (http://www.gcbi.com.cn) tool addressing differential expression based on parameters of fold change ≥ 1.2, *P* value < 0.05, *Q* value < 0.05. miRNA expression levels were recorded as normalized values of corresponding probes.

### Prediction of DE-miR gene targets

Targetscan and miRanda were used to predict gene targets of DE-miRs. Only those target genes predicted by both Targetscan and miRanda were further analyzed.

### Function and pathway enrichment analysis

The GCBI platform was used to analyze functions and pathways for genes of interest identified as potential targets of miRNA downregulation. Gene Ontology (GO, http://www.geneontology.org) and Kyoto Encyclopedia of Genes and Genomes (KEGG, http://www.kegg.jp/) were employed to determine biological processes and enriched pathways, respectively. The selection criterion for significant GO and KEGG pathway terms was *P* value < 0.05.

### Function and gene regulatory network analyses for DE-miRs

GCBI microRNAGONetwork and microRNAGeneNetwork analyses were applied to construct miRNA-function or miRNA-gene networks. MiRNA-GO or miRNA-gene analyses combined target gene prediction with a gene function database. Regulatory relationships between miRNAs and their functions or core genes were visually presented as networks that could be interactively formed by combining adjacent matrices. These suggested underlying core target genes or functions for a particular miRNA, as well as a certain functional target gene or biological process that had underlying effects on miRNAs. Thus, miRNA importance could be evaluated based upon the degree of node interconnectivity, with core miRNAs, genes, and functions exhibiting higher degrees in the network.

### Western blot

Iliac aorta tissue was removed from each of three AG/NAG randomly matched diabetic rats. Total protein was extracted by using Protein Extraction Kit (Boster, China) following the instructions of the kit. Protein concentration was determined by Bradford method. Equal amount of proteins was loaded into SDS-PAGE gels (12%), and then transferred onto the PVDF membrane. After transfer, the membrane was blocked with 5% non-fat dry milk in Tris-buffered saline (TBS) buffer for 1 h at room temperature. The membrane was incubated with primary antibodies against arpp19 (1:200, Abcam, USA), mdm4 (1:200, Abcam, USA), or β-actin (1:1000, Santa Cruz, USA) at 4 °C overnight, followed by 3 washes with TBST (+ 0.1% Tween-20). The membrane was then incubated with HRP-conjugated secondary antibody (1:5000 diluted in blocking buffer) for 1 h, followed by 3 washes with TBST again, and then detected by using enhanced chemiluminescence reagents (Fuji Japan).

### Statistical analyses

Data were expressed as mean ± SD. Two-way ANOVA was used for statistical analyses. miRNAs were considered to have significant differential expression if they were up- or down-regulated by at least 1.2 fold. Statistical significance was determined as *P* or *Q* value less than 0.05.

## Results

### Diabetic atherosclerotic rat model

**Fig. 1:**
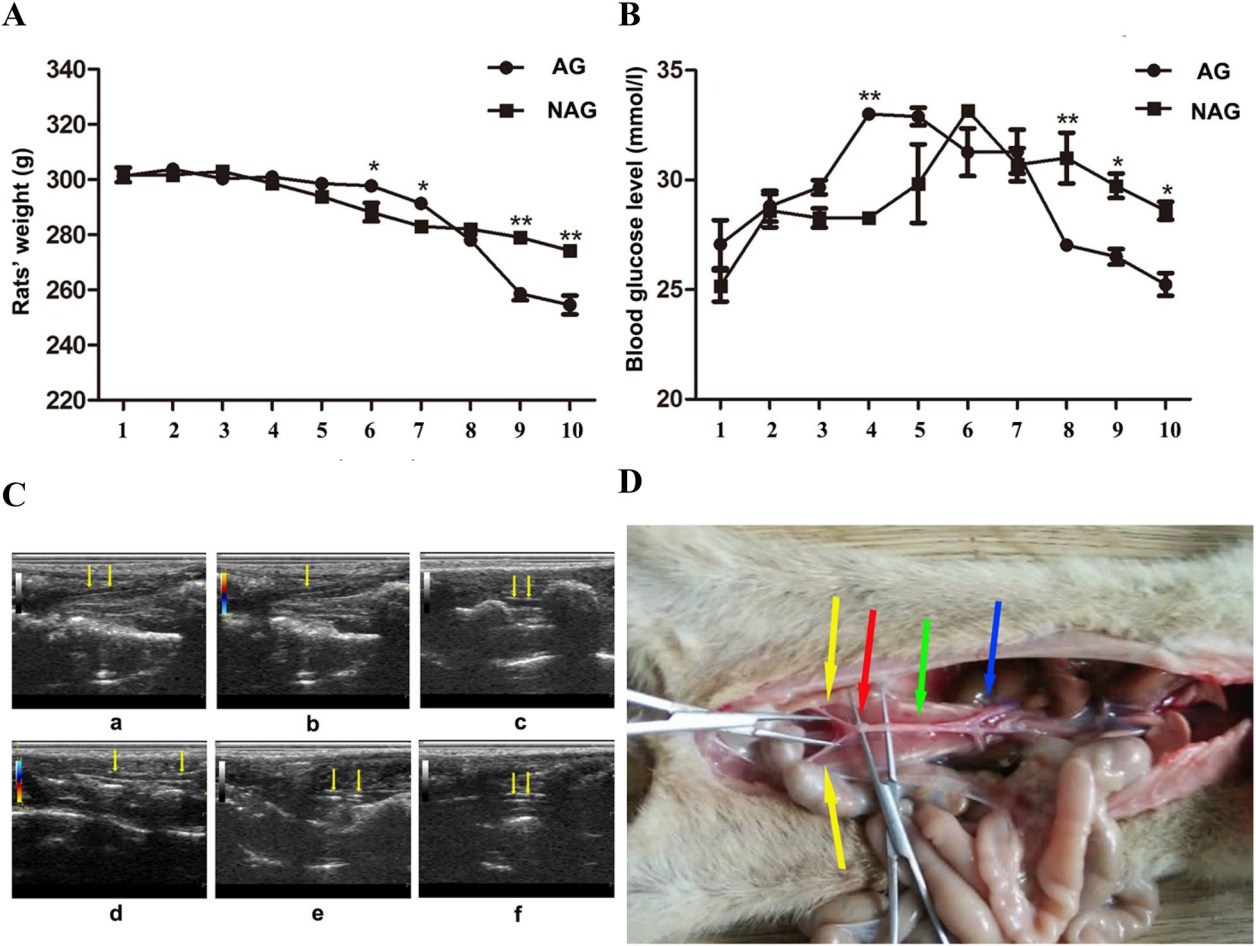
**A** Body weight monitoring of AG and NAG diabetic rats. Rats in the AG group weighed more before week 8, after which time AG rat weight decreased to a statistically significant lower level compared with NAG rats. **B** Random blood glucose levels in AG and NAG diabetic rats. After week 7 the mean blood glucose of NAG rats remained significantly higher than that of AG rats. **C** Doppler ultrasound examination of lesion morphology in iliac artery transverse sections in DA and non-DA rats (NAG: a, b, c; AG: d, e, f). Yellow arrows indicate iliac vascular walls. **D** Aortic, renal, and iliac arteries. Green arrow: aorta. Blue arrow: renal artery. Yellow arrows indicate the iliac artery where samples were collected. Red arrow indicates the iliac artery transverse section analyzed by Doppler ultrasound. *P < 0.05, **P < 0.01

The data on weights and random blood glucose levels of rats after STZ administration are summarized in Fig. 1A, B. Blood glucose levels for all diabetic rats remained > 16.7 mmol/L over the entire monitoring period, demonstrating the stability of the diabetic model. Doppler ultrasound examination of iliac artery transverse sections identified diabetic rats with (AG) and without (NAG) clear formation of atherosclerotic plaques, and three animals were randomly chosen from each group (Fig. 1C). Iliac artery tissue samples were taken (Fig. 1D) for microRNA analysis.

### DE-miR microRNA microarray chip screening

**Fig. 2:**
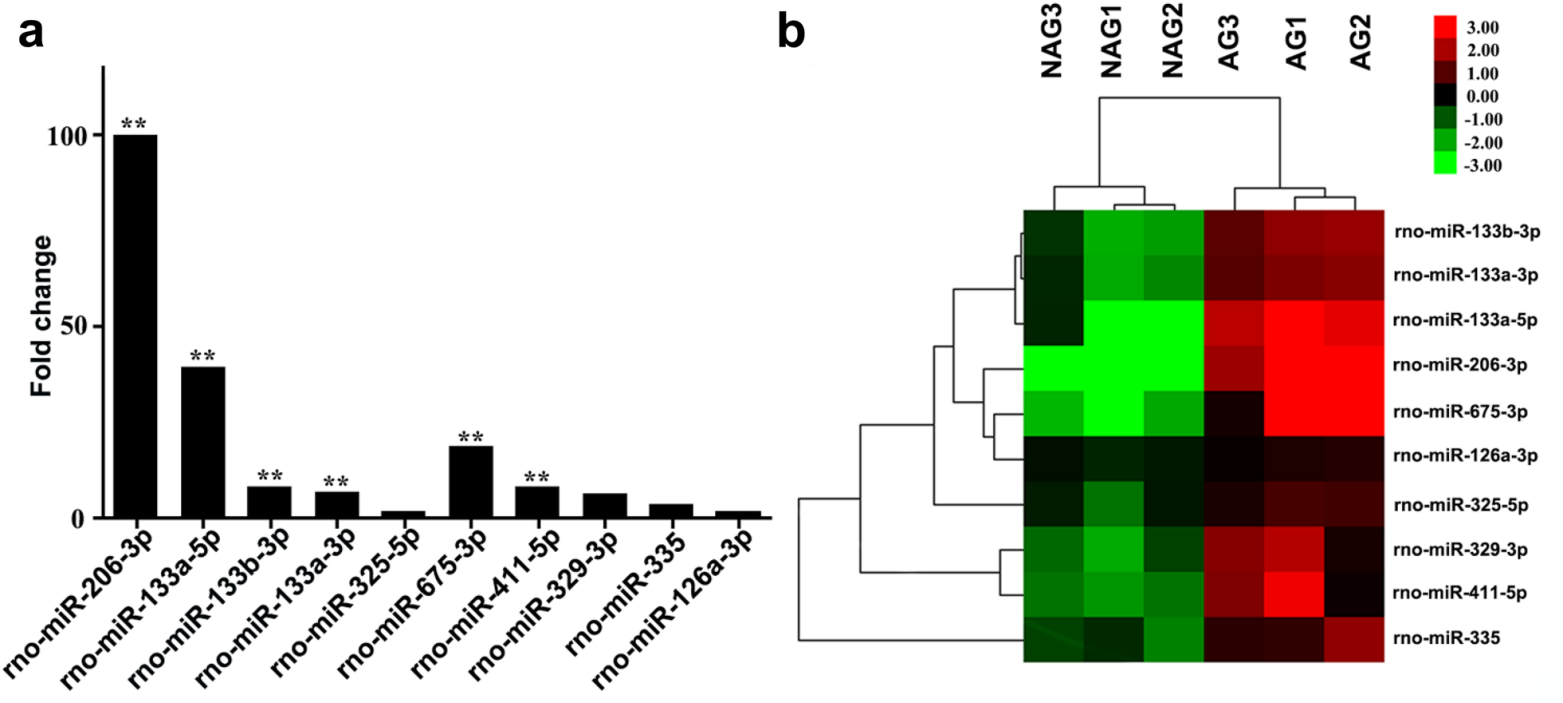
**a** Fold change in expression of 10 DE-miRs. **b** Heatmap of the 10 DE-miRs. The clustering feature trees on the left and above the heat map show stepwise miRNA connections and sample clustering, respectively. MiRNAs shown in red had higher expression levels compared with those in green. **P < 0.01

RNAs from iliac artery tissue samples collected from NAG to AG rats were compared using Affymetrix genechip microRNA 4.0 array. Relative to NAG miRNA levels, AG sample analysis showed 214 down-regulated and 137 up-regulated miRNAs. Average values for each miRNA cluster were normalized for statistical analysis. Setting *Q* value < 0.05 and fold change > 1.2 as parameters, GCBI platform analysis yielded 10 DE-miRs (Fig. 2a), nine of which were up-regulated (comprising rno-miR-206-3p, rno-miR-133a-5p, rno-miR-133b-3p, rno-miR-133a-3p, rno-miR-325-5p, rno-miR-675-3p, rno-miR-411-5p, rno-miR-329-3p, and rno-miR-126a-3p). The most striking differential upregulation was observed for rno-miR-206-3p (99 fold, *P* < 0.01), rno-miR-133a-5p (39 fold, *P* < 0.01) and rno-miR-675-3p (18 fold, *P* < 0.05). Figure 2b shows a heat map presentation of differential AG vs. NAG miRNA expression.

### MicroRNA array validation by qRT-PCR

**Fig. 3:**
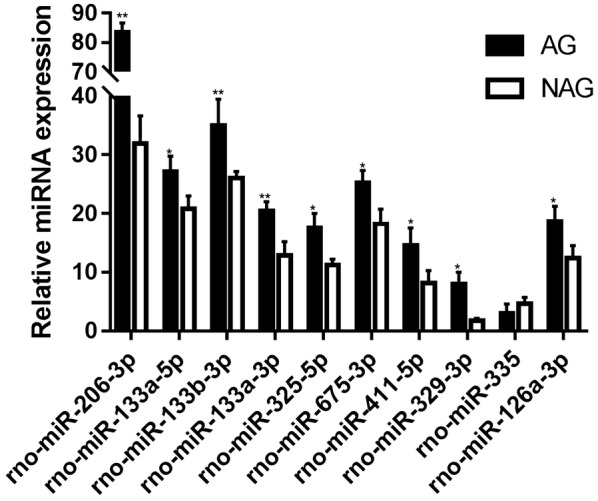
qRT-PCR analysis of relative miRNA expression. *N* = 6

**Fig. 4:**
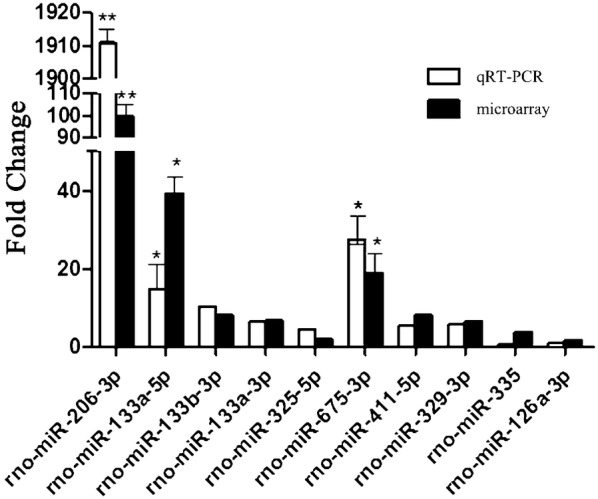
Comparative qRT-PCR and microarray gene chip analysis. *N* = 6

Array results were validated by real-time quantitative PCR analysis (data not shown) of the ten DE-miRs (all AG and NAG RNA samples were pooled for this analysis). qRT-PCR results (Additional file 1: Table S1 and Fig. 3) confirmed AG associated upregulation for nine of the ten DE-miRs, while rno-miR-335 appeared no significant change. With the exception of rno-miR-335 the miRNAs exhibited comparable differential expression levels when assessed by either microarray or qRT-PCR analysis (Additional file 1: Table S2, Fig. 4). rno-miR-335 was not further analyzed.

### Target gene prediction by Targetscan and miRanda

Overlapping results of Targetscan and miRanda analyses of the remaining nine DE-miRs predicted 3349 target genes. Correlations between DE-miRs and predicted target genes are shown in Additional file 1: Table S3.

### DE-miR biological functions and pathways enriched for glucose and lipid metabolism

GO functional enrichment and KEGG pathway analyses indicated that the DE-miRs were significantly enriched in biological processes and pathways related to glucose and lipid metabolism (*P* < 0.05, Additional file 1: Tables S4, S5). These include GO terms “insulin receptor signaling pathway”, “negative regulation of insulin secretion”, “positive regulation of glycolysis”, “response to glucose stimulus”, “cellular response to glucose starvation”, “lipid phosphorylation”, “positive regulation of insulin secretion”, “positive regulation of glucose import”, “glycogen metabolic process”, “cellular response to insulin stimulus”, and “cellular response to glucose stimulus”; and KEGG terms “insulin signaling pathway”, “metabolic pathways”, “insulin secretion”, and “Type II diabetes mellitus”.

### MiRNA-function and miRNA-gene network analyses

**Fig. 5:**
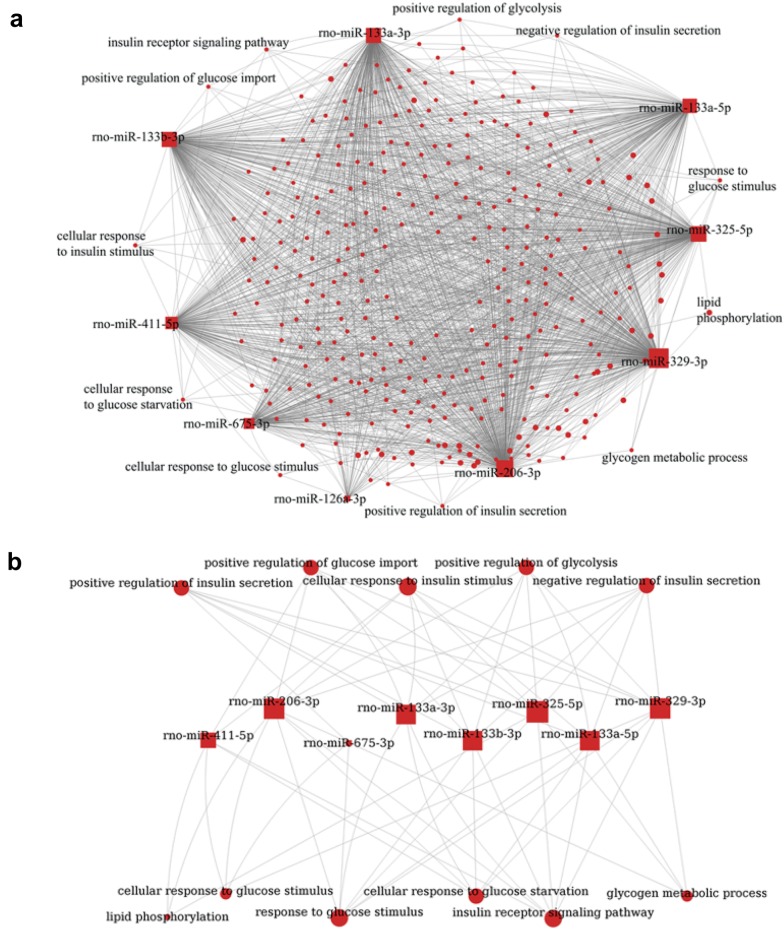
**a** Network of DE-miRs and their enriched functions. The enriched metabolic functions are labeled.** b** DE-miR modulatory pattern of enriched metabolic functions. The enriched glucose and lipid metabolism functions regulated by DE-miRs in which “insulin receptor signaling pathway”, “response to glucose stimulus”, “cellular response to insulin stimulus”, “positive regulation of insulin secretion” and “negative regulation of insulin secretion” were targeted by the most miRNAs, suggesting important roles in DA regulation. rno-miR-329-3p, 133a-5p, 325-5p, 133a-3p, and 133b-3p were predicted to modulate the majority of these metabolic functions

**Fig. 6:**
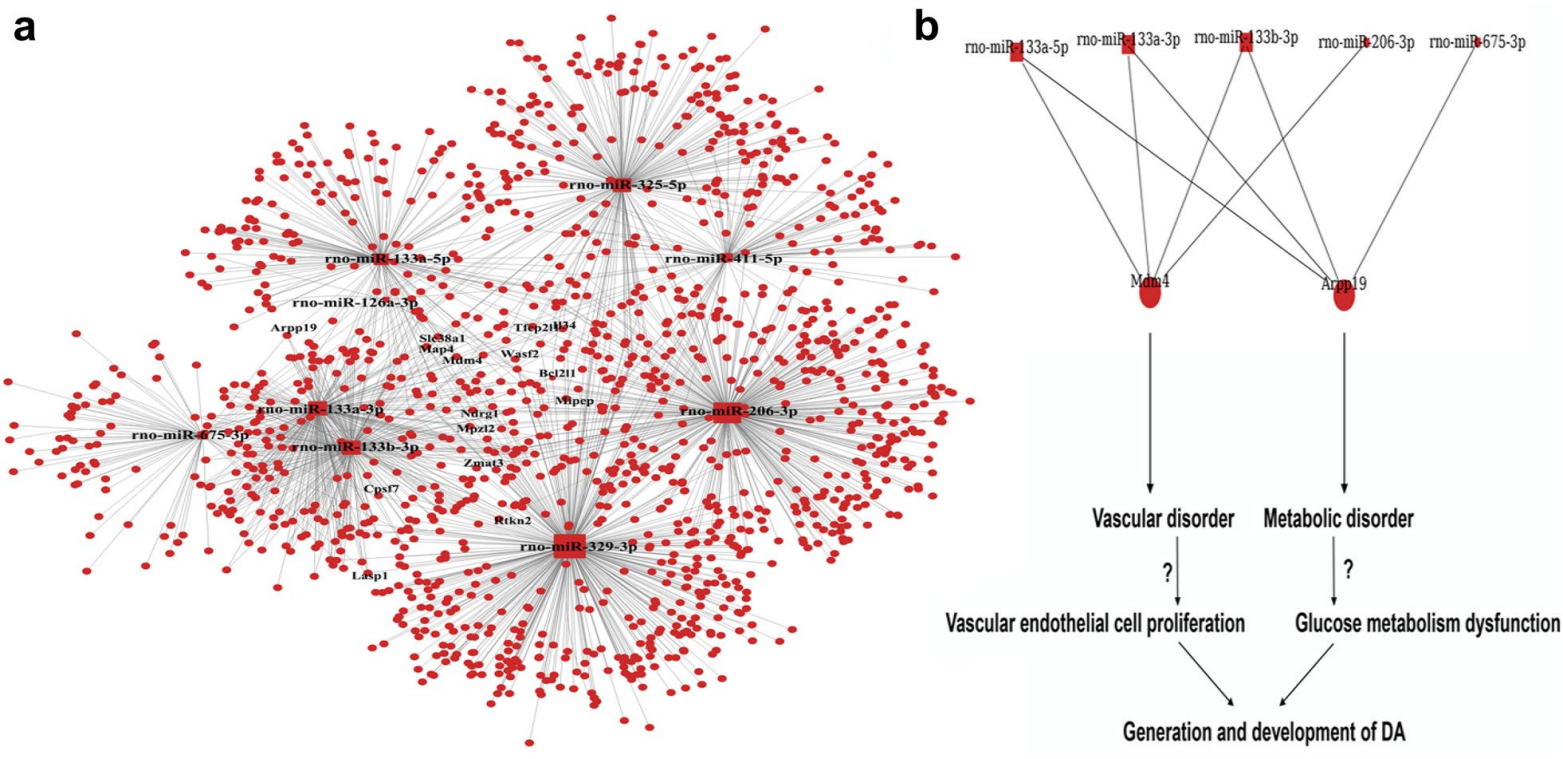
**a** Network of DE-miRs and their target genes. The 15 core target genes regulated by the most DE-miRs (≥ 4) are labeled. **b** MiRNA modulatory pattern of arpp19 and MDM4 and their possible functions in the initiation and development of DA. rno-miR-133a-3p, 133a-5p, and 133b-3p regulated both genes

Based on the function and pathway enrichment results, miRNA-function and miRNA-gene networks were constructed using the GCBI platform (Fig. 5a, Additional file 1: Table S6, Fig. 6a, Additional file 1: Table S7). miRNAs rno-miR-329-3p, rno-miR-206-3p and rno-miR-325-5p targeted the most functions; 304, 285 and 256, respectively (Table 1). The miRNA regulation pattern for glucose and the lipid metabolism functions are depicted in Fig. 3b. The metabolic functions modulated by the most DE-miRs (≥ 6) are comprised of “insulin receptor signaling pathway”, “response to glucose stimulus”, “cellular response to insulin stimulus”, “positive regulation of insulin secretion”, and “negative regulation of insulin secretion” (Fig. 5a, b, Additional file 1: Table S6).

**Table 1:** miRNA-function and miRNA-gene network connectivity

miRNA	Number of modulated functions	Number of modulated genes
rno-miR-329-3p	304	405
rno-miR-206-3p	285	379
rno-miR-325-5p	256	212
rno-miR-133a-3p	245	224
rno-miR-133b-3p	245	225
rno-miR-133a-5p	227	162
rno-miR-411-5p	184	102
rno-miR-675-3p	150	75
rno-miR-126a-3p	44	13

**Fig. 7:**
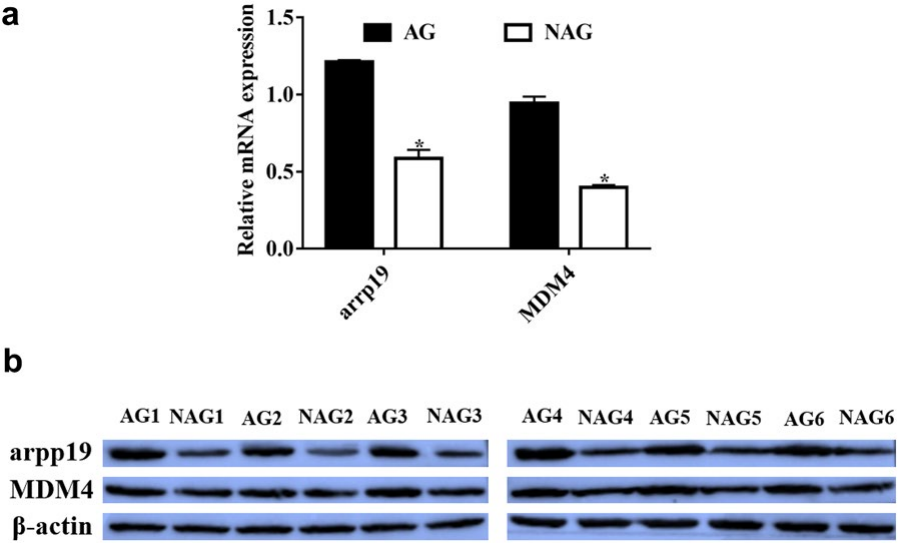
**a** The transcript levels of arpp19 and MDM4 in AG and NAG diabetic rats. **b** The protein levels of arpp19 and MDM4 in AG and NAG diabetic rats. *N* = 6

In the miRNA-gene network rno-miR-329-3p, rno-miR-206-3p, and rno-miR-133b-3p have the most target genes; 405, 379, and 225, respectively (Table 1). Furthermore, the 15 genes Bcl2l1, Arpp19, Cpsf7, Il34, Lasp1, Map4, Mdm4, Mipep, Mpzl2, Ndrg1, Rtkn2, Slc38a1, Tfcp2l1, Wasf2, and Zmat3 were targeted by the most DE-miRs (≥ 4) (Fig. 6a, Additional file 1: Table S7). To further confirm our data, we tested 2 of the central target genes of interest, arpp19 and Mdm4, at the transcript and protein levels in the atherosclerotic and non-atherosclerotic rats. The data showed that both arpp19 and Mdm4 are strikingly increased in atherosclerotic group compared to the non-atherosclerotic group (Fig. 7a, b).

## Discussion

Streptozotocin (STZ) has diabetogenic properties [20, 21] due to its selective destruction of pancreatic islet β-cells thereby causing insulin deficiency [22]. STZ is routinely used to induce diabetes in rats. The STZ-induced diabetic rat model has two major advantages: (1) its close resemblance to human DM with chronic pancreatic islet inflammation, insulitis, and insulin deficiency and (2) cost effectiveness The STZ-treated rat is a well validated animal model for T2DM [23]. Here, we established a diabetic rat model by continuously administering a high-fat, high glucose, high cholesterol diet with a single low dose streptozotocin intervention to effect the partial destruction of β-cells and deficient insulin production. Based on previous research, this regimen produces a T2DM model rat exhibiting a pathophysiological high blood glucose, insulin resistance and lipid metabolism disorder [24]. The diabetic rats thus generated were used to provide insight into potential miRNA involvement in DA.

Combined with contemporary gene expression analysis techniques and miRNA-gene prediction programs, miRNA profiling offers a novel approach to analyzing DA development and progression. We identified 9 out of 728 miRNAs that were differentially up-regulated in iliac aorta tissue samples from atherosclerotic diabetic rats. That these findings support an involvement of miRNAs in DA is strengthened by previous observations showing that arteries with higher levels of miR-126 were more calcified and atherosclerotic with increased miR-126 expression coincident with down-regulated VCAM-1 target gene expression and reduced leukocyte adhesion to endothelial cells [25]. In addition, miR-133 upregulation observed in the present study is consistent with previous observations that miR-133 was increased in patients with acute coronary syndromes [26]. miR-133 is a key regulator of vascular smooth muscle cell phenotypic switching in vitro and in vivo and miR-133 loss-of-function and gain-of-function experiments have shown that miR-133 plays a mechanistic role in VSMC growth, further suggesting a role in vascular disease [27]. Finally, the observed upregulation of miR-206 in AG rat tissues is consistent with previous observations that the presence of atherosusceptible arterial endothelial tissues in high-fat diet-induced diabetic rats was associated with up-regulated miR-206 in pancreatic islets [28].

Because multiple cell types including endothelial cells, macrophages, smooth muscle cells, and fibroblasts all contribute to the abnormal processes underlying diabetic atherosclerosis, it is difficult to precisely elucidate the pathogenic contributions of specific miRNAs. 3349 genes were identified using Targetscan and miRanda as potential regulated targets of the 9 DE-miRs isolated in the present study. KEGG pathway enrichment analysis identified several glucose and lipid metabolism related pathways associated with DA including “metabolic pathways”, “Type II diabetes mellitus”, “insulin signaling pathway”, and “insulin secretion” that are consistent with altered metabolic status of diabetic animals, supporting the relevance of the study approach. In DM patients, “metabolic pathways” and “type II diabetes mellitus” pathway signaling increase the risk of developing DA [29, 30]. Additionally, the insulin related pathway findings are consistent with the major role of insulin signaling in modulating the pathogenesis of both type 2 diabetes and AS [31, 32]. Our GO analysis suggested DA-related processes were enriched in glucose and lipid metabolism (Fig. 5a). Figure 3b shows how these functions were targeted by DE-miRs, especially rno-miR-329-3p, 133a-5p, 325-5p, 133a-3p, and 133b-3p, indicating their extensive involvement in DA pathophysiology. Furthermore, “insulin receptor signaling pathway”, “response to glucose stimulus”, “cellular response to insulin stimulus”, “positive regulation of insulin secretion”, and “negative regulation of insulin secretion” may be important in DA given their high degree of connectivity with the identified DE-miRs.

Among the 15 genes targeted by the most DE-miRs (≥ 4) it may be possible to identify core DA-associated molecular targets. Metabolic and vascular factors can contribute to the generation and progression of DA. It has been reported that arpp19, regulated by four DE-miRs, positively regulates gluconeogenesis [33]. Thus arpp19 may promote DA through the modulation of glucose metabolism under the control of miRNAs. MDM4 is another identified target gene of particular interest. MDM4 is involved in the growth of endothelium and in endothelial cells it plays a critical role in the regulation of the p53/Mdm2-Mdm4 pathway during vascular development [34]. MDM4 may promote vascular endothelial cell proliferation by controlling p53 levels and activity [34]. p53 activation in endothelial cells could inhibit endothelial to mesenchymal transition (EMT) and subsequent proliferation of mesenchymal cells [35, 36]. Also, release of MDM2 from p53 could enable p53 to bind to its target genes to influence cell proliferation and apoptosis in AS pathophysiology [37]. Additionally, p53 could inhibit SMC proliferation and thus interfere with artery formation [38]. Though implicated in the regulation of artery endothelial cell and SMC related processes, the specific mechanisms underlying p53/Mdm2-Mdm4 roles in DA are far from clear. Based on our observations, aberrant expression of candidate core target genes are closely linked to a specific microenvironment in DA. More research is justified to clarify core gene roles in AS, especially under diabetic conditions, and to identify potential differences in gene expression in AS with and without DM. We also found that rno-miR-133a-3p and rno-miR-133b-3p contributed to the regulation of arpp19 and Mdm4, implying non-trivial regulatory roles of these miRNAs in controlling downstream target gene expression and contributing to DA (Fig. 6b). The current findings provide a comprehensive characterization of miRNA expression in DA based on microarray analysis and bioinformatics-based predictions. Further studies are needed to validate miRNA candidate target gene expression and experimentally demonstrate functional roles in DA pathophysiology. Nevertheless, the current study provides valuable direction for larger scale analyses that will combine molecular biological experiments with detailed morphological and immunohistochemical characterizations of diabetes associated vascular lesions, which can yield crucial insight into miRNA roles in specific cell types. This study strongly suggests that miRNAs are indeed associated with the pathophysiology of DA in a useful rat model. We identified nine DE-miRs up-regulated in DA and demonstrated their significant enrichment in lipid and glucose metabolism related processes. By elucidating potential miRNA regulatory patterns among these functions we were able to pinpoint candidate target genes, including arpp19 and MDM4, which may play core roles in DA. These findings identify DE-miRs and/or target genes arpp19 and MDM4 as potential diagnostic markers or therapeutic targets for DA.

## Additional file

**Additional file 1: Table S1.** qRT-PCR analysis of AG vs. NAG relative expression levels. **Table S2.** DE-miR expression measured by miRNA microarray and qRT-PCR. **Table S3.** 3349 predicted target genes of the 9 DE-miRs. **Table S4.** GO analysis of target genes. **Table S5.** Pathway analysis of target genes. **Table S6.** Degree of functions in miRNA-function network. **Table S7.** Degree of target genes in miRNA-gene network.
