# Supplementary material for: MicroRNA profiling of diabetic atherosclerosis in a rat model
Source: Eur J Med Res. 2018 Nov 3;23:55. doi: 10.1186/s40001-018-0354-5 (PMC6215356; doi:10.1186/s40001-018-0354-5)
Supplement: Supplementary file 1 — Additional file 1: Table S1. qRT-PCR analysis of AG vs. NAG relative expression levels. Table S2. DE-miR expression measured by miRNA microarray and qRT-PCR. Table S3. 3349 predicted target genes of the 9 DE-miRs. Table S4. GO analysis of target genes. Table S5. Pathway analysis of target genes. Table S6. Degree of functions in miRNA-function network. Table S7. Degree of target genes in miRNA-gene network. [file 40001_2018_354_MOESM1_ESM.docx]

**Table S1 The result of RT-PCR relative expression level comparing AG with NAG**

|  | Relative MicroRNA expression | | *P value* |
| --- | --- | --- | --- |
|  | AG（n=3） | NAG(n=3) |  |
| rno-miR-206-3p | 20.38±0.574^**^ | 9.48±0.0925 | 0.000 |
| rno-miR-133a-5p | 19.51±0.217^*^ | 15.63±0.134 | 0.001 |
| rno-miR-133b-3p | 7.59±0.202^*^ | 4.22±0.117 | 0.001 |
| rno-miR-133a-3p | 7.91±0.222^*^ | 5.23±0.094 | 0.000 |
| rno-miR-325-5p | 19.78±0.229 | 17.61±0.205 | 0.001 |
| rno-miR-675-3p | 21.33±0.173^*^ | 16.55±0.123 | 0.000 |
| rno-miR-411-5p | 25.28±0.195 | 22.84±0.261 | 0.027 |
| rno-miR-329-3p | 22.44±0.134 | 19.92±0.0815 | 0.001 |
| rno-miR-335 | 16.86±0.112 | 17.46±0.104 | 0.001 |
| rno-miR-126a-3p | 6.11±0.146 | 6.07±0.109 | 0.002 |

**Table S2 DE-miR expression was measured by both miRNA chips and RT-PCR**

| microRNA | Fold change | 2^-△△Ct^ |
| --- | --- | --- |
| rno-miR-206-3p | 99.729425^**^ | 1910.852^**^ |
| rno-miR-133a-5p | 39.119590^*^ | 14.723^*^ |
| rno-miR-133b-3p | 8.134056 | 10.363 |
| rno-miR-133a-3p | 6.593260 | 6.423 |
| rno-miR-325-5p | 1.848884 | 4.500 |
| rno-miR-675-3p | 18.807941^*^ | 27.537^*^ |
| rno-miR-411-5p | 8.039888 | 5.426 |
| rno-miR-329-3p | 6.377054 | 5.736 |
| rno-miR-335 | 3.647267 | 0.660 |
| rno-miR-126a-3p | 1.512667 | 1.033 |

^**^<0.01，^*^<0.05.

**Table S3 3349 predicted target genes of the 9 DE-miRs.**

| Transcript ID | Gene Symbol | miRNA Feature |
| --- | --- | --- |
| rno-miR-133a-3p | Ccdc92 | up |
| rno-miR-133b-3p | Ccdc92 | up |
| rno-miR-411-5p | Zfp867 | up |
| rno-miR-206-3p | Nadk | up |
| rno-miR-133a-5p | Prr24 | up |
| rno-miR-133a-3p | Spns2 | up |
| rno-miR-133b-3p | Spns2 | up |
| rno-miR-133a-5p | Ddx3 | up |
| rno-miR-133a-5p | Nemf | up |
| rno-miR-411-5p | Nemf | up |
| rno-miR-329-3p | Ms4a18 | up |
| rno-miR-329-3p | LOC100359816 | up |
| rno-miR-325-5p | LOC100359937 | up |
| rno-miR-329-3p | Rslcan18 | up |
| rno-miR-325-5p | Tmem207 | up |
| rno-miR-329-3p | Fam57a | up |
| rno-miR-411-5p | Fam57a | up |
| rno-miR-675-3p | LOC100360606 | up |
| rno-miR-133a-3p | Rrp36 | up |
| rno-miR-133b-3p | Rrp36 | up |
| rno-miR-329-3p | Fosb | up |
| rno-miR-329-3p | LOC100361104 | up |
| rno-miR-126a-3p | Kank2 | up |
| rno-miR-675-3p | LOC100361383 | up |
| rno-miR-675-3p | Fam171a2 | up |
| rno-miR-206-3p | Syce1l | up |
| rno-miR-206-3p | Fam196b | up |
| rno-miR-329-3p | Fam196b | up |
| rno-miR-411-5p | Fam196b | up |
| rno-miR-133a-3p | Ociad2 | up |
| rno-miR-133b-3p | Ociad2 | up |
| rno-miR-329-3p | Ociad2 | up |
| rno-miR-206-3p | LOC100361946 | up |
| rno-miR-675-3p | LOC100361946 | up |
| rno-miR-411-5p | LOC100362040 | up |
| rno-miR-206-3p | Ift140 | up |
| rno-miR-133a-3p | LOC100362216 | up |
| rno-miR-133b-3p | LOC100362216 | up |
| rno-miR-325-5p | Mta3 | up |
| rno-miR-133a-5p | Ttc30a | up |
| rno-miR-329-3p | Kmt2d | up |
| rno-miR-325-5p | Epc1 | up |
| rno-miR-206-3p | Cdkl5 | up |
| rno-miR-329-3p | Cdkl5 | up |
| rno-miR-133a-5p | Irgm2 | up |
| rno-miR-206-3p | Ddx55 | up |
| rno-miR-206-3p | Zfp648 | up |
| rno-miR-411-5p | Fam228b | up |
| rno-miR-675-3p | LOC100363228 | up |
| rno-miR-675-3p | Btbd18 | up |
| rno-miR-329-3p | LOC100364568 | up |
| rno-miR-329-3p | Ctdsp2 | up |
| rno-miR-133a-3p | Prdm16 | up |
| rno-miR-133b-3p | Prdm16 | up |
| rno-miR-329-3p | Prdm16 | up |
| rno-miR-325-5p | LOC100909439 | up |
| rno-miR-329-3p | LOC100909443 | up |
| rno-miR-325-5p | Ube2w | up |
| rno-miR-329-3p | LOC100909476 | up |
| rno-miR-329-3p | LOC100909544 | up |
| rno-miR-329-3p | LOC100909590 | up |
| rno-miR-206-3p | LOC100909677 | up |
| rno-miR-133a-5p | LOC100909795 | up |
| rno-miR-206-3p | LOC100909998 | up |
| rno-miR-325-5p | LOC100910021 | up |
| rno-miR-206-3p | LOC100910163 | up |
| rno-miR-329-3p | LOC100910182 | up |
| rno-miR-133a-3p | LOC100910212 | up |
| rno-miR-133b-3p | LOC100910212 | up |
| rno-miR-325-5p | LOC100910212 | up |
| rno-miR-325-5p | LOC100910235 | up |
| rno-miR-133a-3p | LOC100910275 | up |
| rno-miR-133b-3p | LOC100910275 | up |
| rno-miR-675-3p | LOC100910275 | up |
| rno-miR-206-3p | LOC100910278 | up |
| rno-miR-206-3p | LOC100910308 | up |
| rno-miR-206-3p | LOC100910318 | up |
| rno-miR-329-3p | LOC100910506 | up |
| rno-miR-329-3p | LOC100910508 | up |
| rno-miR-206-3p | LOC100910732 | up |
| rno-miR-329-3p | LOC100910772 | up |
| rno-miR-325-5p | LOC100910806 | up |
| rno-miR-411-5p | LOC100910823 | up |
| rno-miR-329-3p | LOC100910843 | up |
| rno-miR-133a-5p | LOC100910848 | up |
| rno-miR-206-3p | LOC100910977 | up |
| rno-miR-329-3p | LOC100910977 | up |
| rno-miR-133a-5p | LOC100910996 | up |
| rno-miR-329-3p | LOC100911069 | up |
| rno-miR-325-5p | LOC100911090 | up |
| rno-miR-206-3p | LOC100911168 | up |
| rno-miR-325-5p | LOC100911168 | up |
| rno-miR-675-3p | LOC100911168 | up |
| rno-miR-411-5p | LOC100911261 | up |
| rno-miR-133a-3p | LOC100911305 | up |
| rno-miR-133b-3p | LOC100911305 | up |
| rno-miR-133a-3p | LOC100911356 | up |
| rno-miR-133b-3p | LOC100911356 | up |
| rno-miR-206-3p | LOC100911357 | up |
| rno-miR-133a-3p | LOC100911365 | up |
| rno-miR-133b-3p | LOC100911365 | up |
| rno-miR-206-3p | LOC100911373 | up |
| rno-miR-675-3p | LOC100911379 | up |
| rno-miR-206-3p | LOC100911440 | up |
| rno-miR-325-5p | LOC100911510 | up |
| rno-miR-206-3p | LOC100911548 | up |
| rno-miR-133a-5p | LOC100911549 | up |
| rno-miR-675-3p | LOC100911572 | up |
| rno-miR-133a-5p | LOC100911581 | up |
| rno-miR-411-5p | LOC100911581 | up |
| rno-miR-133a-3p | LOC100911597 | up |
| rno-miR-133b-3p | LOC100911597 | up |
| rno-miR-329-3p | LOC100911617 | up |
| rno-miR-329-3p | LOC100911625 | up |
| rno-miR-133a-5p | LOC100911674 | up |
| rno-miR-206-3p | LOC100911675 | up |
| rno-miR-325-5p | LOC100911794 | up |
| rno-miR-133a-3p | LOC100911807 | up |
| rno-miR-133b-3p | LOC100911807 | up |
| rno-miR-206-3p | Zfp182 | up |
| rno-miR-329-3p | LOC100911837 | up |
| rno-miR-325-5p | LOC100911867 | up |
| rno-miR-133a-3p | LOC100911951 | up |
| rno-miR-133b-3p | LOC100911951 | up |
| rno-miR-133a-5p | Mipol1 | up |
| rno-miR-329-3p | LOC100912019 | up |
| rno-miR-133a-3p | LOC100912062 | up |
| rno-miR-133b-3p | LOC100912062 | up |
| rno-miR-133a-5p | LOC100912151 | up |
| rno-miR-206-3p | LOC100912259 | up |
| rno-miR-206-3p | LOC100912347 | up |
| rno-miR-206-3p | LOC100912447 | up |
| rno-miR-206-3p | LOC100912450 | up |
| rno-miR-329-3p | LOC100912483 | up |
| rno-miR-329-3p | LOC100912524 | up |
| rno-miR-206-3p | LOC100912527 | up |
| rno-miR-675-3p | LOC100912578 | up |
| rno-miR-133a-3p | LOC100912604 | up |
| rno-miR-133b-3p | LOC100912604 | up |
| rno-miR-206-3p | LOC100912609 | up |
| rno-miR-329-3p | LOC100912948 | up |
| rno-miR-133a-3p | LOC102546312 | up |
| rno-miR-133a-5p | LOC102546312 | up |
| rno-miR-133b-3p | LOC102546312 | up |
| rno-miR-325-5p | LOC102546572 | up |
| rno-miR-329-3p | LOC102546572 | up |
| rno-miR-133a-5p | LOC102546678 | up |
| rno-miR-329-3p | LOC102546678 | up |
| rno-miR-206-3p | LOC102546754 | up |
| rno-miR-411-5p | LOC102546793 | up |
| rno-miR-329-3p | LOC102546809 | up |
| rno-miR-133a-3p | LOC102546838 | up |
| rno-miR-133b-3p | LOC102546838 | up |
| rno-miR-133a-5p | LOC102547059 | up |
| rno-miR-329-3p | LOC102547505 | up |
| rno-miR-675-3p | LOC102547505 | up |
| rno-miR-411-5p | LOC102547626 | up |
| rno-miR-206-3p | LOC102548129 | up |
| rno-miR-325-5p | LOC102548133 | up |
| rno-miR-133a-3p | LOC102548151 | up |
| rno-miR-133b-3p | LOC102548151 | up |
| rno-miR-329-3p | LOC102548320 | up |
| rno-miR-329-3p | LOC102548604 | up |
| rno-miR-411-5p | LOC102548695 | up |
| rno-miR-325-5p | LOC102549471 | up |
| rno-miR-325-5p | LOC102549542 | up |
| rno-miR-133a-5p | LOC102549548 | up |
| rno-miR-329-3p | LOC102549812 | up |
| rno-miR-206-3p | LOC102549817 | up |
| rno-miR-329-3p | LOC102550026 | up |
| rno-miR-133a-5p | LOC102550188 | up |
| rno-miR-133a-5p | LOC102550196 | up |
| rno-miR-206-3p | LOC102550438 | up |
| rno-miR-206-3p | LOC102550588 | up |
| rno-miR-411-5p | LOC102550711 | up |
| rno-miR-206-3p | LOC102551296 | up |
| rno-miR-325-5p | LOC102551337 | up |
| rno-miR-206-3p | LOC102551819 | up |
| rno-miR-206-3p | LOC102551901 | up |
| rno-miR-133a-5p | LOC102551971 | up |
| rno-miR-411-5p | LOC102552068 | up |
| rno-miR-133a-5p | LOC102552286 | up |
| rno-miR-329-3p | LOC102552640 | up |
| rno-miR-133a-3p | LOC102552988 | up |
| rno-miR-133b-3p | LOC102552988 | up |
| rno-miR-206-3p | LOC102552996 | up |
| rno-miR-206-3p | LOC102553158 | up |
| rno-miR-325-5p | LOC102553158 | up |
| rno-miR-329-3p | LOC102553158 | up |
| rno-miR-329-3p | LOC102553270 | up |
| rno-miR-329-3p | LOC102553278 | up |
| rno-miR-206-3p | LOC102553656 | up |
| rno-miR-329-3p | LOC102553656 | up |
| rno-miR-133a-5p | LOC102553670 | up |
| rno-miR-206-3p | LOC102553670 | up |
| rno-miR-325-5p | LOC102553760 | up |
| rno-miR-329-3p | LOC102553760 | up |
| rno-miR-675-3p | LOC102554015 | up |
| rno-miR-325-5p | LOC102554034 | up |
| rno-miR-329-3p | LOC102554034 | up |
| rno-miR-133a-3p | LOC102554183 | up |
| rno-miR-133b-3p | LOC102554183 | up |
| rno-miR-325-5p | LOC102554315 | up |
| rno-miR-411-5p | LOC102554605 | up |
| rno-miR-206-3p | LOC102554611 | up |
| rno-miR-325-5p | LOC102554611 | up |
| rno-miR-325-5p | LOC102554663 | up |
| rno-miR-329-3p | LOC102554884 | up |
| rno-miR-325-5p | LOC102555319 | up |
| rno-miR-675-3p | LOC102555319 | up |
| rno-miR-411-5p | LOC102555622 | up |
| rno-miR-206-3p | LOC102556205 | up |
| rno-miR-329-3p | LOC102556290 | up |
| rno-miR-133a-3p | LOC102556353 | up |
| rno-miR-133b-3p | LOC102556353 | up |
| rno-miR-133a-3p | LOC103689943 | up |
| rno-miR-133b-3p | LOC103689943 | up |
| rno-miR-206-3p | Impad1 | up |
| rno-miR-329-3p | LOC103689978 | up |
| rno-miR-325-5p | LOC103689988 | up |
| rno-miR-325-5p | LOC103690003 | up |
| rno-miR-133a-3p | LOC103690006 | up |
| rno-miR-133b-3p | LOC103690006 | up |
| rno-miR-329-3p | LOC103690007 | up |
| rno-miR-329-3p | Ccdc91 | up |
| rno-miR-325-5p | LOC103690017 | up |
| rno-miR-325-5p | Haus1 | up |
| rno-miR-133a-5p | LOC103690050 | up |
| rno-miR-133a-3p | LOC103690052 | up |
| rno-miR-133b-3p | LOC103690052 | up |
| rno-miR-206-3p | LOC103690069 | up |
| rno-miR-206-3p | LOC103690070 | up |
| rno-miR-329-3p | LOC103690082 | up |
| rno-miR-133a-5p | LOC103690085 | up |
| rno-miR-206-3p | LOC103690119 | up |
| rno-miR-329-3p | Hoxa2 | up |
| rno-miR-206-3p | LOC103690137 | up |
| rno-miR-329-3p | LOC103690156 | up |
| rno-miR-325-5p | LOC103690160 | Up |
| rno-miR-206-3p | LOC103690165 | up |
| rno-miR-206-3p | LOC103690174 | up |
| rno-miR-133a-5p | Tceal5 | up |
| rno-miR-411-5p | Tceal5 | up |
| rno-miR-325-5p | LOC103690230 | up |
| rno-miR-206-3p | LOC103690365 | up |
| rno-miR-133a-3p | LOC103690502 | up |
| rno-miR-133b-3p | LOC103690502 | up |
| rno-miR-329-3p | LOC103690502 | up |
| rno-miR-133a-5p | LOC103690519 | up |
| rno-miR-206-3p | LOC103690519 | up |
| rno-miR-133a-5p | LOC103690937 | up |
| rno-miR-411-5p | LOC103690937 | up |
| rno-miR-133a-3p | Bsph1 | up |
| rno-miR-133b-3p | Bsph1 | up |
| rno-miR-206-3p | LOC103691155 | up |
| rno-miR-206-3p | LOC103691166 | up |
| rno-miR-329-3p | LOC103691166 | up |
| rno-miR-325-5p | LOC103691235 | up |
| rno-miR-206-3p | LOC103691544 | up |
| rno-miR-206-3p | LOC103691556 | up |
| rno-miR-329-3p | LOC103691564 | up |
| rno-miR-411-5p | LOC103691636 | up |
| rno-miR-675-3p | LOC103691808 | up |
| rno-miR-206-3p | LOC103692093 | up |
| rno-miR-675-3p | LOC103692093 | up |
| rno-miR-206-3p | LOC103692111 | up |
| rno-miR-329-3p | Hoxa11 | up |
| rno-miR-325-5p | LOC103692147 | up |
| rno-miR-329-3p | Tlx2 | up |
| rno-miR-325-5p | Ino80b | up |
| rno-miR-206-3p | LOC103692304 | up |
| rno-miR-325-5p | LOC103692360 | up |
| rno-miR-206-3p | LOC103692385 | up |
| rno-miR-329-3p | LOC103692579 | up |
| rno-miR-329-3p | LOC103692699 | up |
| rno-miR-133a-5p | LOC103692792 | up |
| rno-miR-133a-3p | LOC103692813 | up |
| rno-miR-133b-3p | LOC103692813 | up |
| rno-miR-133a-5p | LOC103692841 | up |
| rno-miR-329-3p | LOC103692841 | up |
| rno-miR-325-5p | LOC103692848 | up |
| rno-miR-206-3p | LOC103692958 | up |
| rno-miR-133a-3p | LOC103692976 | up |
| rno-miR-133b-3p | LOC103692976 | up |
| rno-miR-325-5p | LOC103692995 | up |
| rno-miR-133a-5p | LOC103693084 | up |
| rno-miR-329-3p | LOC103693084 | up |
| rno-miR-329-3p | LOC103693254 | up |
| rno-miR-329-3p | LOC103693257 | up |
| rno-miR-329-3p | LOC103693321 | up |
| rno-miR-206-3p | Zbed2 | up |
| rno-miR-133a-3p | LOC103693608 | up |
| rno-miR-133b-3p | LOC103693608 | up |
| rno-miR-675-3p | LOC103693608 | up |
| rno-miR-329-3p | LOC103693647 | up |
| rno-miR-329-3p | Zbed6 | up |
| rno-miR-329-3p | LOC103693776 | up |
| rno-miR-411-5p | LOC103693919 | up |
| rno-miR-329-3p | LOC103694037 | up |
| rno-miR-329-3p | LOC103694079 | up |
| rno-miR-133a-3p | LOC103694430 | up |
| rno-miR-133b-3p | LOC103694430 | up |
| rno-miR-206-3p | LOC103694543 | up |
| rno-miR-206-3p | Cml3 | up |
| rno-miR-411-5p | Prnd | up |
| rno-miR-329-3p | Gpx5 | up |
| rno-miR-206-3p | Clec2d | up |
| rno-miR-329-3p | Snurf | up |
| rno-miR-325-5p | Pom121 | up |
| rno-miR-329-3p | Pom121 | up |
| rno-miR-133a-5p | Ctnnd2 | up |
| rno-miR-133a-3p | Inpp5e | up |
| rno-miR-133b-3p | Inpp5e | up |
| rno-miR-206-3p | Fcnb | up |
| rno-miR-411-5p | Cysltr1 | up |
| rno-miR-329-3p | Sc5d | up |
| rno-miR-325-5p | Dnm1l | up |
| rno-miR-206-3p | Gng4 | up |
| rno-miR-133a-5p | Spata2 | up |
| rno-miR-133a-3p | Chek2 | up |
| rno-miR-133b-3p | Chek2 | up |
| rno-miR-329-3p | Insl3 | up |
| rno-miR-325-5p | Nox1 | up |
| rno-miR-411-5p | Slfn3 | up |
| rno-miR-206-3p | Pde6h | up |
| rno-miR-329-3p | Nek2 | up |
| rno-miR-206-3p | Braf | up |
| rno-miR-411-5p | Pou4f1 | up |
| rno-miR-133a-3p | Msx3 | up |
| rno-miR-133b-3p | Msx3 | up |
| rno-miR-133a-5p | Clasp2 | up |
| rno-miR-133a-5p | Arhgef7 | up |
| rno-miR-329-3p | Arhgef7 | up |
| rno-miR-325-5p | Pitpnb | up |
| rno-miR-133a-5p | Ubqln1 | up |
| rno-miR-206-3p | Ubqln1 | up |
| rno-miR-329-3p | Wbp4 | up |
| rno-miR-133a-3p | Ccnc | up |
| rno-miR-133b-3p | Ccnc | up |
| rno-miR-133a-5p | Klf3 | up |
| rno-miR-133a-3p | Tjp2 | up |
| rno-miR-133b-3p | Tjp2 | up |
| rno-miR-411-5p | Ifngr1 | up |
| rno-miR-133a-3p | Stx1a | up |
| rno-miR-133b-3p | Stx1a | up |
| rno-miR-329-3p | F2rl3 | up |
| rno-miR-206-3p | Pou6f1 | up |
| rno-miR-206-3p | Csnk2a1 | up |
| rno-miR-325-5p | Pdk1 | up |
| rno-miR-329-3p | Akap8 | up |
| rno-miR-133a-5p | Eif4ebp1 | up |
| rno-miR-206-3p | Lgals8 | up |
| rno-miR-329-3p | Lgals8 | up |
| rno-miR-126a-3p | Cplx2 | up |
| rno-miR-206-3p | Cplx2 | up |
| rno-miR-329-3p | Cplx2 | up |
| rno-miR-329-3p | Sdc3 | up |
| rno-miR-411-5p | Jdp2 | up |
| rno-miR-329-3p | Nfasc | up |
| rno-miR-329-3p | Cyth3 | up |
| rno-miR-133a-5p | Kcnd1 | up |
| rno-miR-133a-3p | Pip4k2a | up |
| rno-miR-133b-3p | Pip4k2a | up |
| rno-miR-206-3p | Pip4k2a | up |
| rno-miR-133a-5p | Lpar1 | up |
| rno-miR-133a-3p | Pcdhgc3 | up |
| rno-miR-133b-3p | Pcdhgc3 | up |
| rno-miR-206-3p | Gps1 | up |
| rno-miR-411-5p | Nln | up |
| rno-miR-133a-5p | Rraga | up |
| rno-miR-329-3p | Rraga | up |
| rno-miR-206-3p | Eif4e | up |
| rno-miR-325-5p | Arfrp1 | up |
| rno-miR-329-3p | Kcnj12 | up |
| rno-miR-325-5p | Nlgn2 | up |
| rno-miR-133a-3p | Scarb2 | up |
| rno-miR-133b-3p | Scarb2 | up |
| rno-miR-329-3p | Zbtb7a | up |
| rno-miR-325-5p | Acsl6 | up |
| rno-miR-133a-5p | Gpr176 | up |
| rno-miR-329-3p | Rps6ka2 | up |
| rno-miR-411-5p | Prpsap2 | up |
| rno-miR-133a-3p | Pfkfb3 | up |
| rno-miR-133b-3p | Pfkfb3 | up |
| rno-miR-206-3p | Hnrnpu | up |
| rno-miR-325-5p | Hnrnpu | up |
| rno-miR-325-5p | Nsmf | up |
| rno-miR-206-3p | Baiap2 | up |
| rno-miR-675-3p | Sv2b | up |
| rno-miR-206-3p | Tpm3 | up |
| rno-miR-325-5p | Tpm3 | up |
| rno-miR-329-3p | Tpm3 | up |
| rno-miR-133a-3p | Sv2a | up |
| rno-miR-133b-3p | Sv2a | up |
| rno-miR-325-5p | Ackr2 | up |
| rno-miR-325-5p | Pcyt1a | up |
| rno-miR-325-5p | Inadl | up |
| rno-miR-329-3p | Ppfia4 | up |
| rno-miR-325-5p | Sytl4 | up |
| rno-miR-329-3p | Sytl4 | up |
| rno-miR-206-3p | Rcan2 | up |
| rno-miR-133a-5p | Gabra4 | up |
| rno-miR-206-3p | Baalc | up |
| rno-miR-133a-3p | Caskin1 | up |
| rno-miR-133b-3p | Caskin1 | up |
| rno-miR-325-5p | Arhgef5 | up |
| rno-miR-329-3p | Bnip3l | up |
| rno-miR-329-3p | Pde7b | up |
| rno-miR-329-3p | Bnip1 | up |
| rno-miR-206-3p | Higd1a | up |
| rno-miR-206-3p | Arf3 | up |
| rno-miR-329-3p | Slc28a3 | up |
| rno-miR-206-3p | Paics | up |
| rno-miR-329-3p | Lcp2 | up |
| rno-miR-133a-3p | Tmprss2 | up |
| rno-miR-133b-3p | Tmprss2 | up |
| rno-miR-126a-3p | Slc38a1 | up |
| rno-miR-133a-3p | Slc38a1 | up |
| rno-miR-133a-5p | Slc38a1 | up |
| rno-miR-133b-3p | Slc38a1 | up |
| rno-miR-675-3p | Slc38a4 | up |
| rno-miR-329-3p | Snrk | up |
| rno-miR-329-3p | Ndel1 | up |
| rno-miR-206-3p | Kcnq2 | up |
| rno-miR-329-3p | Lfng | up |
| rno-miR-329-3p | Pik3ca | up |
| rno-miR-329-3p | A1cf | up |
| rno-miR-133a-3p | Marf1 | up |
| rno-miR-133b-3p | Marf1 | up |
| rno-miR-329-3p | Ptpn21 | up |
| rno-miR-329-3p | Trak2 | up |
| rno-miR-325-5p | Shank2 | up |
| rno-miR-325-5p | Rasd2 | up |
| rno-miR-133a-5p | Cdc25a | up |
| rno-miR-329-3p | Ndrg2 | up |
| rno-miR-206-3p | Ehhadh | up |
| rno-miR-206-3p | Taf9b | up |
| rno-miR-329-3p | Taf9b | up |
| rno-miR-675-3p | Zhx1 | up |
| rno-miR-133a-5p | Epha7 | up |
| rno-miR-206-3p | Epha7 | up |
| rno-miR-325-5p | Epha7 | up |
| rno-miR-133a-3p | Nlgn3 | up |
| rno-miR-133b-3p | Nlgn3 | up |
| rno-miR-206-3p | Il12rb2 | up |
| rno-miR-206-3p | Cox8a | up |
| rno-miR-206-3p | Dnah1 | up |
| rno-miR-133a-3p | Foxc2 | up |
| rno-miR-133b-3p | Foxc2 | up |
| rno-miR-206-3p | Slc12a5 | up |
| rno-miR-325-5p | Clstn2 | up |
| rno-miR-675-3p | Clstn2 | up |
| rno-miR-206-3p | Elovl5 | up |
| rno-miR-206-3p | Dcxr | up |
| rno-miR-329-3p | Ipmk | up |
| rno-miR-329-3p | Osgin1 | up |
| rno-miR-133a-5p | Rnf38 | up |
| rno-miR-133a-3p | Dkk3 | up |
| rno-miR-133b-3p | Dkk3 | up |
| rno-miR-329-3p | Prpf18 | up |
| rno-miR-206-3p | Ero1l | up |
| rno-miR-206-3p | Ttl | up |
| rno-miR-329-3p | Dnm3 | up |
| rno-miR-411-5p | Dnm3 | up |
| rno-miR-329-3p | Ahnak | up |
| rno-miR-133a-3p | Pou3f3 | up |
| rno-miR-133b-3p | Pou3f3 | up |
| rno-miR-133a-5p | Pou3f1 | up |
| rno-miR-206-3p | Tgoln2 | up |
| rno-miR-329-3p | Tnrc6b | up |
| rno-miR-133a-5p | Podxl | up |
| rno-miR-206-3p | Podxl | up |
| rno-miR-329-3p | Podxl | up |
| rno-miR-329-3p | Pvrl1 | up |
| rno-miR-325-5p | Syt17 | up |
| rno-miR-206-3p | Svs3a | up |
| rno-miR-133a-3p | Gls2 | up |
| rno-miR-133b-3p | Gls2 | up |
| rno-miR-325-5p | Ppap2b | up |
| rno-miR-206-3p | Ppp1r3b | up |
| rno-miR-325-5p | Ppp1r2 | up |
| rno-miR-206-3p | Lamc2 | up |
| rno-miR-325-5p | Lamc2 | up |
| rno-miR-206-3p | Wbp2 | up |
| rno-miR-411-5p | Prokr1 | up |
| rno-miR-325-5p | Bsnd | up |
| rno-miR-325-5p | Rin1 | up |
| rno-miR-133a-3p | Pkn2 | up |
| rno-miR-133b-3p | Pkn2 | up |
| rno-miR-325-5p | Adcyap1r1 | up |
| rno-miR-133a-3p | Adra2c | up |
| rno-miR-133b-3p | Adra2c | up |
| rno-miR-206-3p | Agtr1a | up |
| rno-miR-133a-3p | Alpi | up |
| rno-miR-133b-3p | Alpi | up |
| rno-miR-126a-3p | Cacna1c | up |
| rno-miR-206-3p | Cebpa | up |
| rno-miR-206-3p | Cftr | up |
| rno-miR-329-3p | Chrm3 | up |
| rno-miR-133a-5p | Cyp21a1 | up |
| rno-miR-206-3p | Ddc | up |
| rno-miR-133a-5p | Drd2 | up |
| rno-miR-206-3p | Edn1 | up |
| rno-miR-133a-5p | Egfr | up |
| rno-miR-329-3p | Eno2 | up |
| rno-miR-133a-5p | Epo | up |
| rno-miR-206-3p | Ets1 | up |
| rno-miR-133a-3p | Fancc | up |
| rno-miR-133b-3p | Fancc | up |
| rno-miR-329-3p | Fancc | up |
| rno-miR-411-5p | Fst | up |
| rno-miR-133a-3p | Gck | up |
| rno-miR-133b-3p | Gck | up |
| rno-miR-325-5p | Glra2 | up |
| rno-miR-325-5p | Grin2a | up |
| rno-miR-206-3p | Igf1 | up |
| rno-miR-133a-3p | Il6r | up |
| rno-miR-133b-3p | Il6r | up |
| rno-miR-133a-3p | Irf1 | up |
| rno-miR-133b-3p | Irf1 | up |
| rno-miR-133a-3p | Slco2a1 | up |
| rno-miR-133b-3p | Slco2a1 | up |
| rno-miR-675-3p | Mos | up |
| rno-miR-206-3p | Cd200 | up |
| rno-miR-206-3p | Gucy2g | up |
| rno-miR-206-3p | Exoc8 | up |
| rno-miR-325-5p | Mme | up |
| rno-miR-675-3p | Gpr116 | up |
| rno-miR-329-3p | Rpl15 | up |
| rno-miR-133a-5p | Fas | up |
| rno-miR-133a-5p | Ptpn4 | up |
| rno-miR-206-3p | Ptpn4 | up |
| rno-miR-133a-5p | Bmf | up |
| rno-miR-325-5p | Wdr44 | up |
| rno-miR-325-5p | Nexn | up |
| rno-miR-206-3p | Fgd4 | up |
| rno-miR-675-3p | Kif27 | up |
| rno-miR-133a-3p | Ppp2r2d | up |
| rno-miR-133b-3p | Ppp2r2d | up |
| rno-miR-133a-5p | Pde4d | up |
| rno-miR-675-3p | Atrx | up |
| rno-miR-411-5p | Cep104 | up |
| rno-miR-325-5p | Arfgap1 | up |
| rno-miR-133a-5p | Serpina4 | up |
| rno-miR-133a-3p | Pfkfb2 | up |
| rno-miR-133b-3p | Pfkfb2 | up |
| rno-miR-206-3p | Pla2g4a | up |
| rno-miR-206-3p | Plcb1 | up |
| rno-miR-133a-5p | Ppm1a | up |
| rno-miR-206-3p | Ppm1b | up |
| rno-miR-133a-3p | Klhl17 | up |
| rno-miR-133b-3p | Klhl17 | up |
| rno-miR-206-3p | Slc25a25 | up |
| rno-miR-329-3p | Gimap5 | up |
| rno-miR-325-5p | Rara | up |
| rno-miR-329-3p | Rbp2 | up |
| rno-miR-133a-3p | RT1-N3 | up |
| rno-miR-133b-3p | RT1-N3 | up |
| rno-miR-206-3p | Cxcl12 | up |
| rno-miR-325-5p | Cxcl12 | up |
| rno-miR-133a-3p | Slc4a1 | up |
| rno-miR-133b-3p | Slc4a1 | up |
| rno-miR-206-3p | Slc4a1 | up |
| rno-miR-206-3p | Vamp2 | up |
| rno-miR-329-3p | Vamp2 | up |
| rno-miR-329-3p | Tacr1 | up |
| rno-miR-329-3p | Thrb | up |
| rno-miR-133a-3p | Bcl2l1 | up |
| rno-miR-133b-3p | Bcl2l1 | up |
| rno-miR-206-3p | Bcl2l1 | up |
| rno-miR-325-5p | Bcl2l1 | up |
| rno-miR-329-3p | Bcl2l1 | up |
| rno-miR-675-3p | Cyp2a2 | up |
| rno-miR-133a-3p | LOC24906 | up |
| rno-miR-133b-3p | LOC24906 | up |
| rno-miR-133a-3p | Cd4 | up |
| rno-miR-133b-3p | Cd4 | up |
| rno-miR-325-5p | Insr | up |
| rno-miR-329-3p | Glul | up |
| rno-miR-329-3p | Gipr | up |
| rno-miR-411-5p | Pigr | up |
| rno-miR-133a-3p | Glp1r | up |
| rno-miR-133b-3p | Glp1r | up |
| rno-miR-325-5p | Glp1r | up |
| rno-miR-329-3p | Hk2 | up |
| rno-miR-133a-5p | Tnfrsf8 | up |
| rno-miR-675-3p | Scarb1 | up |
| rno-miR-411-5p | Chrna5 | up |
| rno-miR-206-3p | Rgn | up |
| rno-miR-206-3p | Itga1 | up |
| rno-miR-133a-3p | Scnn1a | up |
| rno-miR-133b-3p | Scnn1a | up |
| rno-miR-329-3p | Myo5b | up |
| rno-miR-206-3p | Map1a | up |
| rno-miR-325-5p | Map1a | up |
| rno-miR-329-3p | Syk | up |
| rno-miR-329-3p | Cdkn2b | up |
| rno-miR-411-5p | Cdkn2b | up |
| rno-miR-329-3p | Zfp386 | up |
| rno-miR-206-3p | Il6st | up |
| rno-miR-133a-5p | Gucy1b2 | up |
| rno-miR-206-3p | Tspy1 | up |
| rno-miR-206-3p | Onecut1 | up |
| rno-miR-329-3p | Adrbk1 | up |
| rno-miR-325-5p | Arnt2 | up |
| rno-miR-329-3p | Arnt2 | up |
| rno-miR-325-5p | Bdkrb2 | up |
| rno-miR-325-5p | Dspp | up |
| rno-miR-325-5p | Pdgfa | up |
| rno-miR-325-5p | Pdgfra | up |
| rno-miR-329-3p | Pdgfra | up |
| rno-miR-133a-5p | Abcd3 | up |
| rno-miR-329-3p | Mapk10 | up |
| rno-miR-206-3p | Gclc | up |
| rno-miR-206-3p | Zc3hav1 | up |
| rno-miR-325-5p | Zc3hav1 | up |
| rno-miR-133a-3p | Gdf7 | up |
| rno-miR-133b-3p | Gdf7 | up |
| rno-miR-675-3p | Stx17 | up |
| rno-miR-325-5p | Rapgef4 | up |
| rno-miR-133a-3p | Adcy6 | up |
| rno-miR-133b-3p | Adcy6 | up |
| rno-miR-133a-5p | Sra1 | up |
| rno-miR-133a-5p | Pcdhga4 | up |
| rno-miR-133a-3p | Pcdhga9 | up |
| rno-miR-133b-3p | Pcdhga9 | up |
| rno-miR-133a-3p | Pcdhga11 | up |
| rno-miR-133b-3p | Pcdhga11 | up |
| rno-miR-206-3p | Dgat2 | up |
| rno-miR-206-3p | Slc38a3 | up |
| rno-miR-329-3p | Slc38a3 | up |
| rno-miR-329-3p | Aqp4 | up |
| rno-miR-329-3p | Chrna7 | up |
| rno-miR-133a-3p | Fgf1 | up |
| rno-miR-133b-3p | Fgf1 | up |
| rno-miR-206-3p | Ghrhr | up |
| rno-miR-329-3p | Lamp1 | up |
| rno-miR-329-3p | Mat1a | up |
| rno-miR-126a-3p | Vcam1 | up |
| rno-miR-206-3p | Adk | up |
| rno-miR-133a-3p | Adora2a | up |
| rno-miR-133b-3p | Adora2a | up |
| rno-miR-329-3p | Adrbk2 | up |
| rno-miR-133a-3p | Arrb1 | up |
| rno-miR-133b-3p | Arrb1 | up |
| rno-miR-133a-5p | Casp3 | up |
| rno-miR-206-3p | Cd44 | up |
| rno-miR-329-3p | Crmp1 | up |
| rno-miR-206-3p | Cyp7a1 | up |
| rno-miR-206-3p | Sparcl1 | up |
| rno-miR-329-3p | Fshr | up |
| rno-miR-133a-5p | Gabrb1 | up |
| rno-miR-133a-3p | Gdnf | up |
| rno-miR-133a-5p | Gdnf | up |
| rno-miR-133b-3p | Gdnf | up |
| rno-miR-411-5p | Gfra1 | up |
| rno-miR-206-3p | Icam1 | up |
| rno-miR-133a-3p | Il1rap | up |
| rno-miR-133b-3p | Il1rap | up |
| rno-miR-206-3p | Myo1e | up |
| rno-miR-325-5p | Nid1 | up |
| rno-miR-329-3p | Notch1 | up |
| rno-miR-325-5p | Npm1 | up |
| rno-miR-133a-3p | Phex | up |
| rno-miR-133b-3p | Phex | up |
| rno-miR-325-5p | Phex | up |
| rno-miR-329-3p | Lypla1 | up |
| rno-miR-675-3p | Sstr4 | up |
| rno-miR-133a-5p | Abcc8 | up |
| rno-miR-133a-3p | Ube2i | up |
| rno-miR-133b-3p | Ube2i | up |
| rno-miR-329-3p | Ube2i | up |
| rno-miR-206-3p | Utrn | up |
| rno-miR-329-3p | Oprm1 | up |
| rno-miR-133a-5p | Tub | up |
| rno-miR-325-5p | Tub | up |
| rno-miR-206-3p | Ptprz1 | up |
| rno-miR-329-3p | Hspa5 | up |
| rno-miR-411-5p | Acadsb | up |
| rno-miR-411-5p | Smad3 | up |
| rno-miR-675-3p | Gnai3 | up |
| rno-miR-325-5p | Atp1b1 | up |
| rno-miR-206-3p | Fn1 | up |
| rno-miR-206-3p | Dgkg | up |
| rno-miR-329-3p | Vldlr | up |
| rno-miR-411-5p | Vldlr | up |
| rno-miR-133a-5p | Mxi1 | up |
| rno-miR-329-3p | Slc11a2 | up |
| rno-miR-675-3p | Slc11a2 | up |
| rno-miR-133a-5p | Igf1r | up |
| rno-miR-329-3p | Igf1r | up |
| rno-miR-133a-5p | Got2 | up |
| rno-miR-133a-5p | Prkar1a | up |
| rno-miR-206-3p | Hck | up |
| rno-miR-411-5p | Plcg1 | up |
| rno-miR-206-3p | Pfkl | up |
| rno-miR-206-3p | Nf2 | up |
| rno-miR-133a-3p | Ppara | up |
| rno-miR-133b-3p | Ppara | up |
| rno-miR-329-3p | Ppara | up |
| rno-miR-411-5p | Maob | up |
| rno-miR-411-5p | Cpt1a | up |
| rno-miR-325-5p | Sdr9c7 | up |
| rno-miR-206-3p | Dock9 | up |
| rno-miR-329-3p | Zfp335 | up |
| rno-miR-675-3p | Brs3 | up |
| rno-miR-675-3p | Gpr56 | up |
| rno-miR-133a-3p | Sfxn5 | up |
| rno-miR-133a-5p | Sfxn5 | up |
| rno-miR-133b-3p | Sfxn5 | up |
| rno-miR-133a-3p | Ptpn9 | up |
| rno-miR-133b-3p | Ptpn9 | up |
| rno-miR-325-5p | Slc12a8 | up |
| rno-miR-325-5p | Klhl12 | up |
| rno-miR-133a-5p | Cst12 | up |
| rno-miR-325-5p | Rimbp2 | up |
| rno-miR-329-3p | Rimbp2 | up |
| rno-miR-206-3p | Zfp187 | up |
| rno-miR-133a-3p | Has3 | up |
| rno-miR-133b-3p | Has3 | up |
| rno-miR-206-3p | Has3 | up |
| rno-miR-133a-3p | Lrrcc1 | up |
| rno-miR-133b-3p | Lrrcc1 | up |
| rno-miR-329-3p | Sclt1 | up |
| rno-miR-133a-5p | Rims4 | up |
| rno-miR-675-3p | Hbp1 | up |
| rno-miR-325-5p | Impa2 | up |
| rno-miR-133a-3p | Dlgap3 | up |
| rno-miR-133b-3p | Dlgap3 | up |
| rno-miR-325-5p | Vps54 | up |
| rno-miR-206-3p | Elavl2 | up |
| rno-miR-325-5p | Vom2r40 | up |
| rno-miR-329-3p | Zfp689 | up |
| rno-miR-329-3p | Rogdi | up |
| rno-miR-325-5p | Cldn9 | up |
| rno-miR-133a-5p | Kctd5 | up |
| rno-miR-675-3p | Pgp | up |
| rno-miR-325-5p | Tmem204 | up |
| rno-miR-206-3p | Ergic1 | up |
| rno-miR-206-3p | Rnf145 | up |
| rno-miR-133a-3p | Nlrp3 | up |
| rno-miR-133b-3p | Nlrp3 | up |
| rno-miR-325-5p | Nlrp3 | up |
| rno-miR-206-3p | Pfas | up |
| rno-miR-329-3p | Fxr2 | up |
| rno-miR-206-3p | Zbtb4 | up |
| rno-miR-206-3p | Mnt | up |
| rno-miR-329-3p | Rpa1 | up |
| rno-miR-325-5p | Tnfaip1 | up |
| rno-miR-325-5p | Sarm1 | up |
| rno-miR-675-3p | Sarm1 | up |
| rno-miR-329-3p | Blmh | up |
| rno-miR-329-3p | Rhbdl3 | up |
| rno-miR-325-5p | Slfn14 | up |
| rno-miR-325-5p | Vezf1 | up |
| rno-miR-329-3p | Vezf1 | up |
| rno-miR-133a-5p | Wfikkn2 | up |
| rno-miR-329-3p | Socs7 | up |
| rno-miR-206-3p | Ddx5 | up |
| rno-miR-133a-3p | Cep112 | up |
| rno-miR-133b-3p | Cep112 | up |
| rno-miR-329-3p | Gprc5c | up |
| rno-miR-133a-3p | Recql5 | up |
| rno-miR-133b-3p | Recql5 | up |
| rno-miR-133a-3p | Chmp6 | up |
| rno-miR-133b-3p | Chmp6 | up |
| rno-miR-206-3p | Dgcr8 | up |
| rno-miR-206-3p | Lpp | up |
| rno-miR-675-3p | Zdhhc19 | up |
| rno-miR-206-3p | Mylk | up |
| rno-miR-325-5p | Kpna1 | up |
| rno-miR-325-5p | Poglut1 | up |
| rno-miR-329-3p | Naa50 | up |
| rno-miR-133a-3p | Sidt1 | up |
| rno-miR-133b-3p | Sidt1 | up |
| rno-miR-325-5p | Hoxd4 | up |
| rno-miR-329-3p | B3galt5 | up |
| rno-miR-329-3p | Wrb | up |
| rno-miR-329-3p | Chaf1b | up |
| rno-miR-325-5p | Ncam2 | up |
| rno-miR-206-3p | Lipi | up |
| rno-miR-675-3p | Qtrtd1 | up |
| rno-miR-133a-5p | Katnal1 | up |
| rno-miR-206-3p | Cyp2w1 | up |
| rno-miR-133a-3p | Get4 | up |
| rno-miR-133b-3p | Get4 | up |
| rno-miR-329-3p | Syna | up |
| rno-miR-329-3p | Tmem248 | up |
| rno-miR-133a-3p | Mmp17 | up |
| rno-miR-133b-3p | Mmp17 | up |
| rno-miR-133a-3p | Zcchc8 | up |
| rno-miR-133b-3p | Zcchc8 | up |
| rno-miR-133a-5p | Atxn2 | up |
| rno-miR-329-3p | Atxn2 | up |
| rno-miR-133a-3p | Ksr2 | up |
| rno-miR-133b-3p | Ksr2 | up |
| rno-miR-206-3p | Srsf9 | up |
| rno-miR-133a-5p | Sgsm1 | up |
| rno-miR-325-5p | RGD1306556 | up |
| rno-miR-206-3p | Trmt1 | up |
| rno-miR-325-5p | RGD1564093 | up |
| rno-miR-133a-3p | RGD1565158 | up |
| rno-miR-133b-3p | RGD1565158 | up |
| rno-miR-329-3p | Thsd7b | up |
| rno-miR-411-5p | Pik3c2b | up |
| rno-miR-206-3p | Adipor1 | up |
| rno-miR-329-3p | Zbtb41 | up |
| rno-miR-675-3p | Rgs18 | up |
| rno-miR-133a-3p | Rgl1 | up |
| rno-miR-133b-3p | Rgl1 | up |
| rno-miR-329-3p | Colgalt2 | up |
| rno-miR-675-3p | Edem3 | up |
| rno-miR-133a-3p | Swt1 | up |
| rno-miR-133b-3p | Swt1 | up |
| rno-miR-206-3p | Swt1 | up |
| rno-miR-206-3p | Hmcn1 | up |
| rno-miR-206-3p | Acbd6 | up |
| rno-miR-133a-5p | Cacybp | up |
| rno-miR-329-3p | Mettl13 | up |
| rno-miR-133a-5p | Mettl11b | up |
| rno-miR-325-5p | Slc19a2 | up |
| rno-miR-329-3p | Gpr161 | up |
| rno-miR-675-3p | Gpr161 | up |
| rno-miR-133a-5p | Creg1 | up |
| rno-miR-325-5p | Vangl2 | up |
| rno-miR-133a-5p | Nhlh1 | up |
| rno-miR-133a-3p | Pex19 | up |
| rno-miR-133b-3p | Pex19 | up |
| rno-miR-329-3p | Grem2 | up |
| rno-miR-206-3p | Adss | up |
| rno-miR-325-5p | Desi2 | up |
| rno-miR-133a-5p | Tfb2m | up |
| rno-miR-411-5p | Sde2 | up |
| rno-miR-133a-3p | Susd4 | up |
| rno-miR-133b-3p | Susd4 | up |
| rno-miR-325-5p | Gpatch2 | up |
| rno-miR-325-5p | Plxna2 | up |
| rno-miR-126a-3p | Camsap2 | up |
| rno-miR-133a-5p | Naa11 | up |
| rno-miR-133a-5p | Sdad1 | up |
| rno-miR-329-3p | Sdad1 | up |
| rno-miR-133a-3p | Fip1l1 | up |
| rno-miR-133b-3p | Fip1l1 | up |
| rno-miR-206-3p | Usp46 | up |
| rno-miR-206-3p | Nfxl1 | up |
| rno-miR-675-3p | Atp8a1 | up |
| rno-miR-206-3p | Ube2k | up |
| rno-miR-206-3p | Rell1 | up |
| rno-miR-206-3p | Nat8l | up |
| rno-miR-329-3p | Zmiz2 | up |
| rno-miR-329-3p | Prorsd1 | up |
| rno-miR-206-3p | Fam3d | up |
| rno-miR-325-5p | Pdhb | up |
| rno-miR-133a-3p | RGD1564324 | up |
| rno-miR-133b-3p | RGD1564324 | up |
| rno-miR-411-5p | RGD1564324 | up |
| rno-miR-675-3p | Ipo4 | up |
| rno-miR-133a-3p | Cab39l | up |
| rno-miR-133b-3p | Cab39l | up |
| rno-miR-133a-5p | Pdlim2 | up |
| rno-miR-126a-3p | Mustn1 | up |
| rno-miR-133a-3p | Zfp488 | up |
| rno-miR-133b-3p | Zfp488 | up |
| rno-miR-206-3p | Zfp488 | up |
| rno-miR-329-3p | Wapal | up |
| rno-miR-329-3p | B3gnt3 | up |
| rno-miR-133a-5p | Mau2 | up |
| rno-miR-206-3p | Gatad2a | up |
| rno-miR-206-3p | Nek1 | up |
| rno-miR-325-5p | Tnks | up |
| rno-miR-329-3p | Ubxn8 | up |
| rno-miR-325-5p | Ash2l | up |
| rno-miR-329-3p | Whsc1l1 | up |
| rno-miR-411-5p | Tm2d2 | up |
| rno-miR-329-3p | LOC290876 | up |
| rno-miR-329-3p | RGD1564807 | up |
| rno-miR-329-3p | Npepo | up |
| rno-miR-329-3p | Faf2 | up |
| rno-miR-411-5p | Ogn | up |
| rno-miR-133a-5p | Fam120a | up |
| rno-miR-206-3p | Gcm2 | up |
| rno-miR-325-5p | Eef1e1 | up |
| rno-miR-206-3p | Nqo2 | up |
| rno-miR-411-5p | Nmt2 | up |
| rno-miR-325-5p | Etl4 | up |
| rno-miR-206-3p | Bsn | up |
| rno-miR-329-3p | Zfp516 | up |
| rno-miR-206-3p | Zfp236 | up |
| rno-miR-411-5p | Zfp236 | up |
| rno-miR-329-3p | Ska1 | up |
| rno-miR-206-3p | Snx2 | up |
| rno-miR-325-5p | Matr3 | up |
| rno-miR-133a-3p | Capn1 | up |
| rno-miR-133b-3p | Capn1 | up |
| rno-miR-329-3p | Rnmt | up |
| rno-miR-133a-5p | Psmg2 | up |
| rno-miR-329-3p | Psmg2 | up |
| rno-miR-133a-3p | Oacyl | up |
| rno-miR-133b-3p | Oacyl | up |
| rno-miR-206-3p | Nars | up |
| rno-miR-133a-5p | Fbln5 | up |
| rno-miR-206-3p | Spry4 | up |
| rno-miR-329-3p | Spry4 | up |
| rno-miR-133a-3p | Pcdhga7 | up |
| rno-miR-133b-3p | Pcdhga7 | up |
| rno-miR-133a-3p | Pcdhga5 | up |
| rno-miR-133b-3p | Pcdhga5 | up |
| rno-miR-206-3p | Pcdhb11 | up |
| rno-miR-675-3p | Hspa9 | up |
| rno-miR-206-3p | Pkd2l2 | up |
| rno-miR-329-3p | Cdc23 | up |
| rno-miR-329-3p | Slc39a6 | up |
| rno-miR-206-3p | Ctsk | up |
| rno-miR-411-5p | Dsc2 | up |
| rno-miR-325-5p | Cdh22 | up |
| rno-miR-329-3p | Tepp | up |
| rno-miR-133a-3p | Gpr97 | up |
| rno-miR-133b-3p | Gpr97 | up |
| rno-miR-206-3p | Gpr97 | up |
| rno-miR-206-3p | Nlrc5 | up |
| rno-miR-411-5p | Psen1 | up |
| rno-miR-206-3p | Mylk3 | up |
| rno-miR-325-5p | Mmaa | up |
| rno-miR-411-5p | Mmaa | up |
| rno-miR-133a-3p | Ces4a | up |
| rno-miR-133b-3p | Ces4a | up |
| rno-miR-329-3p | Fhod1 | up |
| rno-miR-133a-5p | Cmtr2 | up |
| rno-miR-329-3p | Wwox | up |
| rno-miR-133a-3p | Cmip | up |
| rno-miR-133b-3p | Cmip | up |
| rno-miR-329-3p | Gins2 | up |
| rno-miR-329-3p | Pgbd5 | up |
| rno-miR-411-5p | Pgbd5 | up |
| rno-miR-133a-3p | RGD1562218 | up |
| rno-miR-133b-3p | RGD1562218 | up |
| rno-miR-133a-5p | Kiaa0408 | up |
| rno-miR-329-3p | Tmem242 | up |
| rno-miR-329-3p | Pabpc6 | up |
| rno-miR-133a-3p | Smok2a | up |
| rno-miR-133b-3p | Smok2a | up |
| rno-miR-206-3p | Aig1 | up |
| rno-miR-329-3p | Slc1a5 | up |
| rno-miR-206-3p | Psg29 | up |
| rno-miR-133a-3p | Fbxo46 | up |
| rno-miR-133b-3p | Fbxo46 | up |
| rno-miR-329-3p | Irgq | up |
| rno-miR-206-3p | Cyp2c11 | up |
| rno-miR-329-3p | Sipa1l3 | up |
| rno-miR-133a-3p | Lasp1 | up |
| rno-miR-133b-3p | Lasp1 | up |
| rno-miR-329-3p | Lasp1 | up |
| rno-miR-675-3p | Lasp1 | up |
| rno-miR-411-5p | Zfp536 | up |
| rno-miR-133a-3p | Ctu1 | up |
| rno-miR-133b-3p | Ctu1 | up |
| rno-miR-325-5p | Akt1s1 | up |
| rno-miR-133a-3p | Adora1 | up |
| rno-miR-133b-3p | Adora1 | up |
| rno-miR-206-3p | Otog | up |
| rno-miR-329-3p | Otog | up |
| rno-miR-206-3p | Chsy1 | up |
| rno-miR-133a-5p | Arrdc4 | up |
| rno-miR-325-5p | Arrdc4 | up |
| rno-miR-411-5p | Arrdc4 | up |
| rno-miR-206-3p | Akap13 | up |
| rno-miR-329-3p | Akap13 | up |
| rno-miR-206-3p | Cpeb1 | up |
| rno-miR-206-3p | Tmc3 | up |
| rno-miR-325-5p | Arl4a | up |
| rno-miR-133a-3p | Msmb | up |
| rno-miR-133b-3p | Msmb | up |
| rno-miR-329-3p | Stard10 | up |
| rno-miR-133a-3p | Arfip2 | up |
| rno-miR-133b-3p | Arfip2 | up |
| rno-miR-133a-5p | Oprk1 | up |
| rno-miR-325-5p | Oprk1 | up |
| rno-miR-675-3p | Hs3st2 | up |
| rno-miR-133a-5p | Faah | up |
| rno-miR-411-5p | Faah | up |
| rno-miR-133a-3p | Fam57b | up |
| rno-miR-133b-3p | Fam57b | up |
| rno-miR-206-3p | Arl6ip1 | up |
| rno-miR-411-5p | Olr300 | up |
| rno-miR-325-5p | Rhod | up |
| rno-miR-325-5p | Rbm4 | up |
| rno-miR-133a-3p | Otub1 | up |
| rno-miR-133b-3p | Otub1 | up |
| rno-miR-133a-3p | Uncx | up |
| rno-miR-133b-3p | Uncx | up |
| rno-miR-329-3p | Irs2 | up |
| rno-miR-133a-5p | Dtx4 | up |
| rno-miR-329-3p | Acvr1b | up |
| rno-miR-206-3p | Psat1 | up |
| rno-miR-675-3p | Prune2 | up |
| rno-miR-329-3p | Pnma3 | up |
| rno-miR-133a-3p | Dusp9 | up |
| rno-miR-133b-3p | Dusp9 | up |
| rno-miR-325-5p | Mecp2 | up |
| rno-miR-206-3p | Tpd52l3 | up |
| rno-miR-133a-5p | Prrg3 | up |
| rno-miR-329-3p | Prrg3 | up |
| rno-miR-206-3p | Pkd2l1 | up |
| rno-miR-675-3p | Bloc1s2 | up |
| rno-miR-329-3p | Stk10 | up |
| rno-miR-329-3p | Actr1a | up |
| rno-miR-133a-3p | RGD1311783 | up |
| rno-miR-133b-3p | RGD1311783 | up |
| rno-miR-133a-3p | Col17a1 | up |
| rno-miR-133b-3p | Col17a1 | up |
| rno-miR-206-3p | Pank1 | up |
| rno-miR-329-3p | Pank1 | up |
| rno-miR-133a-3p | Ifit2 | up |
| rno-miR-133a-5p | Ifit2 | up |
| rno-miR-133b-3p | Ifit2 | up |
| rno-miR-133a-5p | Papss2 | up |
| rno-miR-206-3p | Papss2 | up |
| rno-miR-675-3p | Adra1a | up |
| rno-miR-329-3p | S1pr2 | up |
| rno-miR-329-3p | Mrps18b | up |
| rno-miR-206-3p | Htt | up |
| rno-miR-206-3p | RT1-Db1 | up |
| rno-miR-329-3p | Brd2 | up |
| rno-miR-329-3p | Lhfpl5 | up |
| rno-miR-133a-5p | Kctd20 | up |
| rno-miR-325-5p | Btbd9 | up |
| rno-miR-206-3p | Pak3 | up |
| rno-miR-411-5p | Pcbp3 | up |
| rno-miR-325-5p | Tfpi | up |
| rno-miR-206-3p | Reep3 | up |
| rno-miR-411-5p | Kpna5 | up |
| rno-miR-206-3p | Nus1 | up |
| rno-miR-206-3p | Fam26e | up |
| rno-miR-133a-5p | Pald1 | up |
| rno-miR-329-3p | Pald1 | up |
| rno-miR-206-3p | Spata9 | up |
| rno-miR-325-5p | Spata9 | up |
| rno-miR-206-3p | Avpr1b | up |
| rno-miR-329-3p | Slc6a6 | up |
| rno-miR-325-5p | Aes | up |
| rno-miR-133a-3p | Enc1 | up |
| rno-miR-133b-3p | Enc1 | up |
| rno-miR-206-3p | Enc1 | up |
| rno-miR-329-3p | Sgtb | up |
| rno-miR-329-3p | Glg1 | up |
| rno-miR-329-3p | Mapt | up |
| rno-miR-133a-3p | Wdr70 | up |
| rno-miR-133b-3p | Wdr70 | up |
| rno-miR-675-3p | Skp2 | up |
| rno-miR-675-3p | Rgs4 | up |
| rno-miR-329-3p | Rai14 | up |
| rno-miR-325-5p | C1qtnf3 | up |
| rno-miR-133a-5p | 6-Mar | up |
| rno-miR-325-5p | Tpd52 | up |
| rno-miR-133a-3p | Mecom | up |
| rno-miR-133b-3p | Mecom | up |
| rno-miR-206-3p | Mecom | up |
| rno-miR-206-3p | Fndc3b | up |
| rno-miR-411-5p | Mccc1 | up |
| rno-miR-325-5p | Tm4sf1 | up |
| rno-miR-411-5p | Tm4sf1 | up |
| rno-miR-411-5p | Nfya | up |
| rno-miR-133a-3p | Mapk13 | up |
| rno-miR-133b-3p | Mapk13 | up |
| rno-miR-329-3p | Scamp1 | up |
| rno-miR-133a-3p | Slc50a1 | up |
| rno-miR-133b-3p | Slc50a1 | up |
| rno-miR-325-5p | Hist2h2be | up |
| rno-miR-133a-3p | Etv3 | up |
| rno-miR-133b-3p | Etv3 | up |
| rno-miR-133a-3p | Amigo1 | up |
| rno-miR-133b-3p | Amigo1 | up |
| rno-miR-411-5p | Vav3 | up |
| rno-miR-411-5p | Ntng1 | up |
| rno-miR-329-3p | Prmt6 | up |
| rno-miR-206-3p | Hsd17b7 | up |
| rno-miR-206-3p | Lppr4 | up |
| rno-miR-206-3p | Ints12 | up |
| rno-miR-329-3p | Metap1 | up |
| rno-miR-206-3p | Lsamp | up |
| rno-miR-329-3p | Prps1 | up |
| rno-miR-329-3p | Phospho2 | up |
| rno-miR-329-3p | Cybrd1 | up |
| rno-miR-329-3p | Mgat3 | up |
| rno-miR-411-5p | Gjb3 | up |
| rno-miR-411-5p | Tgfbr1 | up |
| rno-miR-133a-3p | P2ry2 | up |
| rno-miR-133b-3p | P2ry2 | up |
| rno-miR-329-3p | Atp2b3 | up |
| rno-miR-206-3p | Spred1 | up |
| rno-miR-133a-5p | Tp53bp1 | up |
| rno-miR-325-5p | Ell3 | up |
| rno-miR-206-3p | Stard7 | up |
| rno-miR-325-5p | Ntrk3 | up |
| rno-miR-329-3p | Ntrk3 | up |
| rno-miR-206-3p | RGD1566226 | up |
| rno-miR-325-5p | Tmx4 | up |
| rno-miR-133a-5p | Dstnl1 | up |
| rno-miR-206-3p | Snx5 | up |
| rno-miR-133a-3p | Ralgds | up |
| rno-miR-133b-3p | Ralgds | up |
| rno-miR-329-3p | Snph | up |
| rno-miR-411-5p | Snph | up |
| rno-miR-206-3p | Angpt4 | up |
| rno-miR-325-5p | Srxn1 | up |
| rno-miR-329-3p | Pdrg1 | up |
| rno-miR-133a-5p | Gria3 | up |
| rno-miR-206-3p | Bet1 | up |
| rno-miR-133a-3p | Aar2 | up |
| rno-miR-133b-3p | Aar2 | up |
| rno-miR-325-5p | Aar2 | up |
| rno-miR-329-3p | Ndst1 | up |
| rno-miR-206-3p | Svs3b | up |
| rno-miR-206-3p | Zmynd8 | up |
| rno-miR-133a-3p | Dpm2 | up |
| rno-miR-133b-3p | Dpm2 | up |
| rno-miR-133a-5p | Fam210b | up |
| rno-miR-411-5p | Ptprj | up |
| rno-miR-329-3p | Osbpl2 | up |
| rno-miR-325-5p | Ythdf1 | up |
| rno-miR-329-3p | Cask | up |
| rno-miR-206-3p | Helz2 | up |
| rno-miR-133a-3p | Dlx1 | up |
| rno-miR-133b-3p | Dlx1 | up |
| rno-miR-329-3p | Fut7 | up |
| rno-miR-206-3p | Clic3 | up |
| rno-miR-325-5p | Phpt1 | up |
| rno-miR-675-3p | Lcn9 | up |
| rno-miR-133b-3p | Gdi2 | up |
| rno-miR-206-3p | Fam73b | up |
| rno-miR-133a-3p | Ap1b1 | up |
| rno-miR-133b-3p | Ap1b1 | up |
| rno-miR-325-5p | Prrc2b | up |
| rno-miR-329-3p | Mapkap1 | up |
| rno-miR-133a-3p | Phf19 | up |
| rno-miR-133b-3p | Phf19 | up |
| rno-miR-411-5p | Olfml2a | up |
| rno-miR-133a-5p | Ube2h | up |
| rno-miR-206-3p | Ube2h | up |
| rno-miR-206-3p | Lrguk | up |
| rno-miR-133a-3p | Slc35b4 | up |
| rno-miR-133b-3p | Slc35b4 | up |
| rno-miR-411-5p | Slc35b4 | up |
| rno-miR-325-5p | Bpgm | up |
| rno-miR-206-3p | Kcnj2 | up |
| rno-miR-206-3p | Slc8a1 | up |
| rno-miR-206-3p | Cacna1d | up |
| rno-miR-329-3p | Cacna1d | up |
| rno-miR-325-5p | Kcnj16 | up |
| rno-miR-329-3p | S1pr1 | up |
| rno-miR-329-3p | Mob1a | up |
| rno-miR-325-5p | Gclm | up |
| rno-miR-411-5p | Gclm | up |
| rno-miR-325-5p | Tex261 | up |
| rno-miR-126a-3p | Pik3r2 | up |
| rno-miR-325-5p | Gfpt1 | up |
| rno-miR-329-3p | Gfpt1 | up |
| rno-miR-206-3p | Gkn2 | up |
| rno-miR-329-3p | Copg1 | up |
| rno-miR-411-5p | Kcnmb1 | up |
| rno-miR-133a-5p | Foxp1 | up |
| rno-miR-206-3p | Foxp1 | up |
| rno-miR-329-3p | Edem1 | up |
| rno-miR-206-3p | Sema3a | up |
| rno-miR-133a-3p | Colq | up |
| rno-miR-133b-3p | Colq | up |
| rno-miR-206-3p | Spsb2 | up |
| rno-miR-206-3p | RGD1311164 | up |
| rno-miR-675-3p | Dusp16 | up |
| rno-miR-325-5p | Terf1 | up |
| rno-miR-411-5p | Xkr4 | up |
| rno-miR-133a-5p | Plag1 | up |
| rno-miR-133a-3p | Tp53inp1 | up |
| rno-miR-133b-3p | Tp53inp1 | up |
| rno-miR-126a-3p | Dpy19l4 | up |
| rno-miR-325-5p | Wwp1 | up |
| rno-miR-206-3p | Ctnnal1 | up |
| rno-miR-206-3p | Tmem245 | up |
| rno-miR-411-5p | Tmem245 | up |
| rno-miR-329-3p | Snx30 | up |
| rno-miR-411-5p | Snx30 | up |
| rno-miR-206-3p | Ncbp1 | up |
| rno-miR-325-5p | RGD1305807 | up |
| rno-miR-411-5p | Trmt10b | up |
| rno-miR-206-3p | RGD1304595 | up |
| rno-miR-206-3p | LOC298139 | up |
| rno-miR-206-3p | Frmd3 | up |
| rno-miR-206-3p | Zfp353 | up |
| rno-miR-675-3p | Slc35d1 | up |
| rno-miR-329-3p | Coa7 | up |
| rno-miR-329-3p | Arhgef39 | up |
| rno-miR-133a-3p | Cmpk1 | up |
| rno-miR-133b-3p | Cmpk1 | up |
| rno-miR-329-3p | Nsun4 | up |
| rno-miR-329-3p | Cldn19 | up |
| rno-miR-329-3p | Smap2 | up |
| rno-miR-133a-3p | Zfp46 | up |
| rno-miR-133b-3p | Zfp46 | up |
| rno-miR-133a-5p | Mrto4 | up |
| rno-miR-206-3p | H6pd | up |
| rno-miR-325-5p | Morn1 | up |
| rno-miR-329-3p | B3galt6 | up |
| rno-miR-329-3p | Crim1 | up |
| rno-miR-206-3p | Atl2 | up |
| rno-miR-329-3p | Slc30a6 | up |
| rno-miR-329-3p | Clip4 | up |
| rno-miR-133a-5p | Rab3ip | up |
| rno-miR-325-5p | Slc35f6 | up |
| rno-miR-206-3p | Klhl29 | up |
| rno-miR-206-3p | Pum2 | up |
| rno-miR-133a-3p | Fam49a | up |
| rno-miR-133a-5p | Fam49a | up |
| rno-miR-133b-3p | Fam49a | up |
| rno-miR-325-5p | Fam49a | up |
| rno-miR-675-3p | Fam49a | up |
| rno-miR-325-5p | Scin | up |
| rno-miR-411-5p | Scin | up |
| rno-miR-133a-5p | Nova1 | up |
| rno-miR-206-3p | Eif2s3x | up |
| rno-miR-329-3p | Trappc6b | up |
| rno-miR-411-5p | Klhdc2 | up |
| rno-miR-206-3p | Dhrs7 | up |
| rno-miR-329-3p | Six4 | up |
| rno-miR-206-3p | Rab15 | up |
| rno-miR-329-3p | Zfyve1 | up |
| rno-miR-675-3p | Arel1 | up |
| rno-miR-133a-5p | Rps6kl1 | up |
| rno-miR-329-3p | Cpg1 | up |
| rno-miR-329-3p | Cpsf2 | up |
| rno-miR-325-5p | Asb2 | up |
| rno-miR-325-5p | Brf1 | up |
| rno-miR-675-3p | Brf1 | up |
| rno-miR-206-3p | Camkmt | up |
| rno-miR-411-5p | Apc2 | up |
| rno-miR-206-3p | Ano4 | up |
| rno-miR-411-5p | Myf5 | up |
| rno-miR-133a-5p | Cnot2 | up |
| rno-miR-329-3p | Yeats4 | up |
| rno-miR-133a-5p | Cpsf6 | up |
| rno-miR-133a-3p | Lrig3 | up |
| rno-miR-133b-3p | Lrig3 | up |
| rno-miR-133a-3p | Ndrg1 | up |
| rno-miR-133a-5p | Ndrg1 | up |
| rno-miR-133b-3p | Ndrg1 | up |
| rno-miR-206-3p | Ndrg1 | up |
| rno-miR-133a-3p | Nsmce2 | up |
| rno-miR-133b-3p | Nsmce2 | up |
| rno-miR-325-5p | Grhl2 | up |
| rno-miR-329-3p | Grhl2 | up |
| rno-miR-411-5p | Zc3h3 | up |
| rno-miR-325-5p | Tigd5 | up |
| rno-miR-675-3p | Tigd5 | up |
| rno-miR-329-3p | Pycrl | up |
| rno-miR-329-3p | Gga1 | up |
| rno-miR-411-5p | Rbx1 | up |
| rno-miR-133a-3p | Ldoc1l | up |
| rno-miR-133b-3p | Ldoc1l | up |
| rno-miR-206-3p | Ldoc1l | up |
| rno-miR-329-3p | Ldoc1l | up |
| rno-miR-329-3p | Fam118a | up |
| rno-miR-206-3p | Cerk | up |
| rno-miR-133a-3p | Mov10l1 | up |
| rno-miR-133b-3p | Mov10l1 | up |
| rno-miR-329-3p | Trabd | up |
| rno-miR-133a-3p | Gxylt1 | up |
| rno-miR-133b-3p | Gxylt1 | up |
| rno-miR-329-3p | Gxylt1 | up |
| rno-miR-325-5p | Senp1 | up |
| rno-miR-206-3p | Krt73 | up |
| rno-miR-206-3p | Lhfpl1 | up |
| rno-miR-675-3p | Amot | up |
| rno-miR-206-3p | LOC300308 | up |
| rno-miR-329-3p | Med17 | up |
| rno-miR-206-3p | Gramd1b | up |
| rno-miR-329-3p | Sorl1 | up |
| rno-miR-206-3p | Hinfp | up |
| rno-miR-133a-3p | Mpzl2 | up |
| rno-miR-133b-3p | Mpzl2 | up |
| rno-miR-206-3p | Mpzl2 | up |
| rno-miR-675-3p | Mpzl2 | up |
| rno-miR-329-3p | Tmprss13 | up |
| rno-miR-325-5p | Ubl7 | up |
| rno-miR-133a-3p | Coro2b | up |
| rno-miR-133b-3p | Coro2b | up |
| rno-miR-206-3p | Ptplad1 | up |
| rno-miR-206-3p | Dapk2 | up |
| rno-miR-329-3p | Dapk2 | up |
| rno-miR-329-3p | Rora | up |
| rno-miR-411-5p | Tmem30a | up |
| rno-miR-325-5p | Senp6 | up |
| rno-miR-325-5p | Mthfs | up |
| rno-miR-206-3p | Cacna2d2 | up |
| rno-miR-329-3p | Cacna2d2 | up |
| rno-miR-133a-3p | Wdr6 | up |
| rno-miR-133b-3p | Wdr6 | up |
| rno-miR-133a-3p | Shisa5 | up |
| rno-miR-133b-3p | Shisa5 | up |
| rno-miR-206-3p | Ltf | up |
| rno-miR-329-3p | Ubp1 | up |
| rno-miR-206-3p | Zfp445 | up |
| rno-miR-325-5p | Fyco1 | up |
| rno-miR-206-3p | Xcr1 | up |
| rno-miR-133a-5p | Plcl2 | up |
| rno-miR-133a-3p | Unc5cl | up |
| rno-miR-133a-5p | Unc5cl | up |
| rno-miR-133b-3p | Unc5cl | up |
| rno-miR-329-3p | Unc5cl | up |
| rno-miR-133a-3p | RGD1565959 | up |
| rno-miR-133b-3p | RGD1565959 | up |
| rno-miR-133a-5p | RGD1561662 | up |
| rno-miR-133a-5p | Gltscr1l | up |
| rno-miR-329-3p | Enpp4 | up |
| rno-miR-133a-3p | RGD1309049 | up |
| rno-miR-133b-3p | RGD1309049 | up |
| rno-miR-329-3p | Arhgef4 | up |
| rno-miR-329-3p | Sema4c | up |
| rno-miR-329-3p | Tgfbrap1 | up |
| rno-miR-206-3p | Nif3l1 | up |
| rno-miR-329-3p | Fzd7 | up |
| rno-miR-329-3p | Tns1 | up |
| rno-miR-411-5p | Col4a4 | up |
| rno-miR-411-5p | Cab39 | up |
| rno-miR-133a-5p | Cdh12 | up |
| rno-miR-206-3p | LOC302192 | up |
| rno-miR-133a-5p | Chst7 | up |
| rno-miR-126a-3p | Pof1b | up |
| rno-miR-133a-3p | RGD1560455 | up |
| rno-miR-133a-5p | Foxo4 | up |
| rno-miR-329-3p | Gdpd2 | up |
| rno-miR-133a-5p | Eda | up |
| rno-miR-133a-5p | Mbnl3 | up |
| rno-miR-325-5p | Mbnl3 | up |
| rno-miR-133a-3p | Rap2c | up |
| rno-miR-133b-3p | Rap2c | up |
| rno-miR-325-5p | Cul4b | up |
| rno-miR-329-3p | Nyx | up |
| rno-miR-329-3p | Usp27x | up |
| rno-miR-329-3p | RGD1565862 | up |
| rno-miR-675-3p | Car5b | up |
| rno-miR-206-3p | Syap1 | up |
| rno-miR-325-5p | Eif1ax | up |
| rno-miR-206-3p | Mbtps2 | up |
| rno-miR-133a-3p | Tbl1x | up |
| rno-miR-133b-3p | Tbl1x | up |
| rno-miR-411-5p | Tbl1x | up |
| rno-miR-325-5p | Bcorl1 | up |
| rno-miR-329-3p | Bcorl1 | up |
| rno-miR-329-3p | Rbmx | up |
| rno-miR-675-3p | Mmgt1 | up |
| rno-miR-133a-5p | Glis2 | up |
| rno-miR-206-3p | Glis2 | up |
| rno-miR-675-3p | Adcy9 | up |
| rno-miR-329-3p | Mapk8ip3 | up |
| rno-miR-329-3p | Sh3pxd2b | up |
| rno-miR-675-3p | Sh3pxd2b | up |
| rno-miR-133a-3p | Fbxw11 | up |
| rno-miR-133b-3p | Fbxw11 | up |
| rno-miR-329-3p | Pwwp2a | up |
| rno-miR-675-3p | Thg1l | up |
| rno-miR-206-3p | Adam19 | up |
| rno-miR-411-5p | Trim41 | up |
| rno-miR-133a-3p | 8-Sep | up |
| rno-miR-133b-3p | 8-Sep | up |
| rno-miR-329-3p | RGD1304587 | up |
| rno-miR-325-5p | Specc1 | up |
| rno-miR-411-5p | Mmgt2 | up |
| rno-miR-206-3p | Tnk1 | up |
| rno-miR-329-3p | Dvl2 | up |
| rno-miR-133a-3p | Rtn4rl1 | up |
| rno-miR-133b-3p | Rtn4rl1 | up |
| rno-miR-411-5p | Tmem199 | up |
| rno-miR-133a-5p | Slc46a1 | up |
| rno-miR-133a-3p | Rab11fip4 | up |
| rno-miR-133b-3p | Rab11fip4 | up |
| rno-miR-329-3p | Rab11fip4 | up |
| rno-miR-411-5p | Ssh2 | up |
| rno-miR-411-5p | Atad5 | up |
| rno-miR-325-5p | Lig3 | up |
| rno-miR-675-3p | Lig3 | up |
| rno-miR-329-3p | Slfn5 | up |
| rno-miR-206-3p | Appbp2 | up |
| rno-miR-329-3p | Appbp2 | up |
| rno-miR-329-3p | Tbx4 | up |
| rno-miR-329-3p | Igf2bp1 | up |
| rno-miR-133a-3p | Hoxb3 | up |
| rno-miR-133b-3p | Hoxb3 | up |
| rno-miR-206-3p | Sp2 | up |
| rno-miR-329-3p | Mllt6 | up |
| rno-miR-329-3p | Msl1 | up |
| rno-miR-325-5p | Rundc1 | up |
| rno-miR-329-3p | Kif18b | up |
| rno-miR-206-3p | Acbd4 | up |
| rno-miR-325-5p | 10-Mar | up |
| rno-miR-329-3p | Bptf | up |
| rno-miR-329-3p | Arsg | up |
| rno-miR-206-3p | Abca8a | up |
| rno-miR-206-3p | Cog1 | up |
| rno-miR-411-5p | Nat9 | up |
| rno-miR-329-3p | C1qtnf1 | up |
| rno-miR-329-3p | Mrpl12 | up |
| rno-miR-411-5p | Rab40b | up |
| rno-miR-133a-3p | Tnrc6c | up |
| rno-miR-133b-3p | Tnrc6c | up |
| rno-miR-206-3p | Slc7a4 | up |
| rno-miR-329-3p | Slc7a4 | up |
| rno-miR-329-3p | Senp5 | up |
| rno-miR-411-5p | Muc20 | up |
| rno-miR-325-5p | Dirc2 | up |
| rno-miR-325-5p | Parp14 | up |
| rno-miR-206-3p | Gtf2e1 | up |
| rno-miR-325-5p | RGD1560175 | up |
| rno-miR-329-3p | RGD1560175 | up |
| rno-miR-206-3p | Cep97 | up |
| rno-miR-329-3p | St3gal6 | up |
| rno-miR-329-3p | C2cd2 | up |
| rno-miR-329-3p | Brwd1 | up |
| rno-miR-133a-5p | Ets2 | up |
| rno-miR-133a-5p | Son | up |
| rno-miR-411-5p | Son | up |
| rno-miR-206-3p | Lrrc8e | up |
| rno-miR-325-5p | Gbp1 | up |
| rno-miR-329-3p | Sdk1 | up |
| rno-miR-329-3p | Mmd2 | up |
| rno-miR-411-5p | Tnrc18 | up |
| rno-miR-133a-3p | Ttyh3 | up |
| rno-miR-133b-3p | Ttyh3 | up |
| rno-miR-329-3p | Nxpe5 | up |
| rno-miR-206-3p | Agfg2 | up |
| rno-miR-329-3p | Cldn15 | up |
| rno-miR-206-3p | Gatsl2 | up |
| rno-miR-329-3p | Sbno1 | up |
| rno-miR-325-5p | Oas1d | up |
| rno-miR-133a-3p | Rnft2 | up |
| rno-miR-133b-3p | Rnft2 | up |
| rno-miR-675-3p | Fbxw8 | up |
| rno-miR-133a-5p | Sirt4 | up |
| rno-miR-329-3p | Mlec | up |
| rno-miR-133a-5p | Sez6l | up |
| rno-miR-411-5p | Hps4 | up |
| rno-miR-329-3p | Ung | up |
| rno-miR-329-3p | Tmem119 | up |
| rno-miR-126a-3p | Sart3 | up |
| rno-miR-133a-5p | Orai2 | up |
| rno-miR-329-3p | Orai2 | up |
| rno-miR-329-3p | Zswim4 | up |
| rno-miR-206-3p | Tnpo2 | up |
| rno-miR-133a-5p | Serpinb11 | up |
| rno-miR-329-3p | Bfar | up |
| rno-miR-329-3p | Ercc4 | up |
| rno-miR-133a-3p | Tfcp2l1 | up |
| rno-miR-133b-3p | Tfcp2l1 | up |
| rno-miR-325-5p | Tfcp2l1 | up |
| rno-miR-411-5p | Tfcp2l1 | up |
| rno-miR-206-3p | Ccdc93 | up |
| rno-miR-411-5p | Ccdc93 | up |
| rno-miR-133a-3p | Mdm4 | up |
| rno-miR-133a-5p | Mdm4 | up |
| rno-miR-133b-3p | Mdm4 | up |
| rno-miR-206-3p | Mdm4 | up |
| rno-miR-206-3p | Ppp1r12b | up |
| rno-miR-329-3p | Abl2 | up |
| rno-miR-325-5p | Rabgap1l | up |
| rno-miR-133a-5p | Tbx19 | up |
| rno-miR-325-5p | Pogk | up |
| rno-miR-325-5p | Atf6 | up |
| rno-miR-133a-5p | Tomm40l | up |
| rno-miR-329-3p | Tomm40l | up |
| rno-miR-206-3p | Tagln2 | up |
| rno-miR-126a-3p | Sdccag8 | up |
| rno-miR-206-3p | Sdccag8 | up |
| rno-miR-325-5p | Sdccag8 | up |
| rno-miR-325-5p | Angel2 | up |
| rno-miR-411-5p | Angel2 | up |
| rno-miR-325-5p | Prox1 | up |
| rno-miR-133a-3p | Lamb3 | up |
| rno-miR-133b-3p | Lamb3 | up |
| rno-miR-133a-5p | Zfp644 | up |
| rno-miR-675-3p | Hnrnpdl | up |
| rno-miR-325-5p | Paqr3 | up |
| rno-miR-206-3p | Adamts3 | up |
| rno-miR-133a-5p | Tmprss11e | up |
| rno-miR-325-5p | Rbm47 | up |
| rno-miR-325-5p | N4bp2 | up |
| rno-miR-411-5p | Arap2 | up |
| rno-miR-329-3p | Tapt1 | up |
| rno-miR-206-3p | Gpr125 | up |
| rno-miR-329-3p | Fbxl5 | up |
| rno-miR-133a-3p | Sh3bp2 | up |
| rno-miR-133b-3p | Sh3bp2 | up |
| rno-miR-206-3p | Zfyve28 | up |
| rno-miR-133a-3p | Nelfa | up |
| rno-miR-133b-3p | Nelfa | up |
| rno-miR-206-3p | Prr14l | up |
| rno-miR-329-3p | Prr14l | up |
| rno-miR-206-3p | Rnf215 | up |
| rno-miR-133a-3p | Cobl | up |
| rno-miR-133b-3p | Cobl | up |
| rno-miR-133a-3p | Aftph | up |
| rno-miR-133b-3p | Aftph | up |
| rno-miR-329-3p | Pus10 | up |
| rno-miR-325-5p | Papolg | up |
| rno-miR-206-3p | Bcl11a | up |
| rno-miR-133a-5p | Fancl | up |
| rno-miR-133a-3p | Efemp1 | up |
| rno-miR-133b-3p | Efemp1 | up |
| rno-miR-133a-5p | Dlg5 | up |
| rno-miR-206-3p | Dlg5 | up |
| rno-miR-329-3p | Vcl | up |
| rno-miR-325-5p | Nek10 | up |
| rno-miR-206-3p | Abhd6 | up |
| rno-miR-329-3p | Rcor2 | up |
| rno-miR-133a-3p | Atg14 | up |
| rno-miR-133b-3p | Atg14 | up |
| rno-miR-126a-3p | Zfp219 | up |
| rno-miR-133a-3p | Zfp219 | up |
| rno-miR-133b-3p | Zfp219 | up |
| rno-miR-206-3p | Zfhx2 | up |
| rno-miR-329-3p | Irf9 | up |
| rno-miR-329-3p | RGD1306353 | up |
| rno-miR-329-3p | Lats2 | up |
| rno-miR-133a-5p | Mtmr6 | up |
| rno-miR-329-3p | Wdfy2 | up |
| rno-miR-329-3p | Kif13b | up |
| rno-miR-675-3p | Kif13b | up |
| rno-miR-206-3p | Hmbox1 | up |
| rno-miR-325-5p | Dock5 | up |
| rno-miR-133a-5p | Rhobtb2 | up |
| rno-miR-329-3p | Fam160b2 | up |
| rno-miR-206-3p | Fndc3a | up |
| rno-miR-329-3p | Fndc3a | up |
| rno-miR-133a-5p | Lcp1 | up |
| rno-miR-133a-5p | Pcdh20 | up |
| rno-miR-206-3p | Pcdh9 | up |
| rno-miR-329-3p | Klf12 | up |
| rno-miR-325-5p | Sox21 | up |
| rno-miR-325-5p | Pxk | up |
| rno-miR-329-3p | Pxk | up |
| rno-miR-411-5p | Flnb | up |
| rno-miR-126a-3p | Duxbl1 | up |
| rno-miR-133a-5p | Il17rb | up |
| rno-miR-329-3p | Eaf1 | up |
| rno-miR-325-5p | Ccser2 | up |
| rno-miR-206-3p | Med26 | up |
| rno-miR-133a-5p | Psd3 | up |
| rno-miR-206-3p | Psd3 | up |
| rno-miR-411-5p | Psd3 | up |
| rno-miR-329-3p | Mtus1 | up |
| rno-miR-329-3p | Gtf2e2 | up |
| rno-miR-325-5p | Htra4 | up |
| rno-miR-133a-5p | Agpat5 | up |
| rno-miR-325-5p | Agpat5 | up |
| rno-miR-206-3p | Amt | up |
| rno-miR-325-5p | Tcta | up |
| rno-miR-329-3p | Tubgcp3 | up |
| rno-miR-133a-3p | Atp11a | up |
| rno-miR-133b-3p | Atp11a | up |
| rno-miR-133a-3p | Kbtbd11 | up |
| rno-miR-133b-3p | Kbtbd11 | up |
| rno-miR-325-5p | Arhgef10 | up |
| rno-miR-206-3p | Abhd13 | up |
| rno-miR-329-3p | Abhd13 | up |
| rno-miR-675-3p | Abhd13 | up |
| rno-miR-675-3p | Irx4 | up |
| rno-miR-329-3p | Ice1 | up |
| rno-miR-206-3p | Nsd1 | up |
| rno-miR-329-3p | Arl10 | up |
| rno-miR-329-3p | Bicd2 | up |
| rno-miR-329-3p | Phf2 | up |
| rno-miR-325-5p | Gfod1 | up |
| rno-miR-329-3p | Slc35b3 | up |
| rno-miR-206-3p | Fars2 | up |
| rno-miR-206-3p | Lrrc16a | up |
| rno-miR-329-3p | Lrrc16a | up |
| rno-miR-206-3p | Zfp438 | up |
| rno-miR-329-3p | Neto1 | up |
| rno-miR-133a-5p | Cndp1 | up |
| rno-miR-206-3p | Rnf165 | up |
| rno-miR-206-3p | Me2 | up |
| rno-miR-133a-3p | Fam210a | up |
| rno-miR-133b-3p | Fam210a | up |
| rno-miR-206-3p | Spire1 | up |
| rno-miR-206-3p | Onecut2 | up |
| rno-miR-133a-5p | Tcerg1 | up |
| rno-miR-329-3p | RGD735029 | up |
| rno-miR-133a-5p | Pura | up |
| rno-miR-206-3p | Pura | up |
| rno-miR-325-5p | Pura | up |
| rno-miR-329-3p | Cdc25c | up |
| rno-miR-206-3p | Ammecr1l | up |
| rno-miR-329-3p | Dsg2 | up |
| rno-miR-206-3p | Zfp521 | up |
| rno-miR-329-3p | Elac1 | up |
| rno-miR-133a-3p | Cdh16 | up |
| rno-miR-133b-3p | Cdh16 | up |
| rno-miR-206-3p | Csnk2a2 | up |
| rno-miR-329-3p | Ciapin1 | up |
| rno-miR-329-3p | Papd5 | up |
| rno-miR-675-3p | Usp38 | up |
| rno-miR-325-5p | Esrp2 | up |
| rno-miR-329-3p | Tango6 | up |
| rno-miR-206-3p | Nfat5 | up |
| rno-miR-329-3p | Nfat5 | up |
| rno-miR-675-3p | Nfat5 | up |
| rno-miR-675-3p | Sdr42e1 | up |
| rno-miR-133a-5p | RGD1304884 | up |
| rno-miR-329-3p | Gse1 | up |
| rno-miR-133a-3p | Snai3 | up |
| rno-miR-133b-3p | Snai3 | up |
| rno-miR-206-3p | Ccsap | up |
| rno-miR-133a-3p | Disc1 | up |
| rno-miR-133b-3p | Disc1 | up |
| rno-miR-325-5p | Disc1 | up |
| rno-miR-206-3p | Nhlrc2 | up |
| rno-miR-325-5p | Ablim1 | up |
| rno-miR-325-5p | Hspa12a | up |
| rno-miR-329-3p | Pdzd8 | up |
| rno-miR-329-3p | Nkd2 | up |
| rno-miR-675-3p | Zfp541 | up |
| rno-miR-206-3p | Rgs17 | up |
| rno-miR-133a-3p | Iyd | up |
| rno-miR-133b-3p | Iyd | up |
| rno-miR-133a-3p | Cyp26c1 | up |
| rno-miR-133b-3p | Cyp26c1 | up |
| rno-miR-329-3p | Chd1 | up |
| rno-miR-206-3p | Wdr27 | up |
| rno-miR-325-5p | Lats1 | up |
| rno-miR-133a-3p | Tab2 | up |
| rno-miR-133b-3p | Tab2 | up |
| rno-miR-206-3p | Tab2 | up |
| rno-miR-411-5p | Zbtb45 | up |
| rno-miR-133a-5p | Gpr126 | up |
| rno-miR-133a-3p | Spred3 | up |
| rno-miR-133b-3p | Spred3 | up |
| rno-miR-206-3p | Spred3 | up |
| rno-miR-325-5p | Cd22 | up |
| rno-miR-675-3p | Dpy19l3 | up |
| rno-miR-133a-5p | Zfp819 | up |
| rno-miR-206-3p | Fv1 | up |
| rno-miR-133a-5p | Heca | up |
| rno-miR-329-3p | Nhsl1 | up |
| rno-miR-675-3p | Ano5 | up |
| rno-miR-329-3p | Synm | up |
| rno-miR-133a-5p | Chd2 | up |
| rno-miR-133a-3p | Mctp2 | up |
| rno-miR-133b-3p | Mctp2 | up |
| rno-miR-206-3p | Mctp2 | up |
| rno-miR-206-3p | Hddc3 | up |
| rno-miR-329-3p | Prc1 | up |
| rno-miR-411-5p | Prc1 | up |
| rno-miR-133a-5p | Adamtsl3 | up |
| rno-miR-206-3p | Adamtsl3 | up |
| rno-miR-325-5p | Adamtsl3 | up |
| rno-miR-329-3p | Usp47 | up |
| rno-miR-206-3p | Wee1 | up |
| rno-miR-329-3p | Kdm8 | up |
| rno-miR-675-3p | Itgal | up |
| rno-miR-133a-3p | Fbxl19 | up |
| rno-miR-133b-3p | Fbxl19 | up |
| rno-miR-206-3p | Ikzf5 | up |
| rno-miR-206-3p | Slc25a22 | up |
| rno-miR-133a-5p | Tnpo1 | up |
| rno-miR-206-3p | Tnpo1 | up |
| rno-miR-206-3p | Ano1 | up |
| rno-miR-133a-3p | Rce1 | up |
| rno-miR-133b-3p | Rce1 | up |
| rno-miR-325-5p | Bbs1 | up |
| rno-miR-133a-3p | Ccdc85b | up |
| rno-miR-133b-3p | Ccdc85b | up |
| rno-miR-133a-3p | Rela | up |
| rno-miR-133b-3p | Rela | up |
| rno-miR-133a-3p | Map3k11 | up |
| rno-miR-133b-3p | Map3k11 | up |
| rno-miR-206-3p | Frmd8 | up |
| rno-miR-133a-3p | Batf2 | up |
| rno-miR-133b-3p | Batf2 | up |
| rno-miR-329-3p | Ttc9c | up |
| rno-miR-325-5p | Fan1 | up |
| rno-miR-133a-5p | Trex2 | up |
| rno-miR-411-5p | Rorb | up |
| rno-miR-133a-3p | RGD1310016 | up |
| rno-miR-133b-3p | RGD1310016 | up |
| rno-miR-329-3p | RGD1311595 | up |
| rno-miR-206-3p | Cstf2t | up |
| rno-miR-133a-3p | Zfyve27 | up |
| rno-miR-133b-3p | Zfyve27 | up |
| rno-miR-206-3p | Loxl4 | up |
| rno-miR-133a-3p | Cox15 | up |
| rno-miR-133b-3p | Cox15 | up |
| rno-miR-133a-5p | Pip5k1b | up |
| rno-miR-206-3p | Sh3pxd2a | up |
| rno-miR-329-3p | Hectd2 | up |
| rno-miR-411-5p | Kif20b | up |
| rno-miR-133a-5p | Lipm | up |
| rno-miR-206-3p | Sorcs1 | up |
| rno-miR-206-3p | Med23 | up |
| rno-miR-206-3p | Trim26 | up |
| rno-miR-206-3p | Trim39 | up |
| rno-miR-325-5p | Ly6g6f | up |
| rno-miR-329-3p | Uhrf1bp1 | up |
| rno-miR-133a-3p | Tcp11 | up |
| rno-miR-133b-3p | Tcp11 | up |
| rno-miR-206-3p | Tmem26 | up |
| rno-miR-133a-3p | Rtkn2 | up |
| rno-miR-133b-3p | Rtkn2 | up |
| rno-miR-206-3p | Rtkn2 | up |
| rno-miR-329-3p | Rtkn2 | up |
| rno-miR-329-3p | Cdk19 | up |
| rno-miR-325-5p | Rev3l | up |
| rno-miR-206-3p | Sec63 | up |
| rno-miR-133a-5p | Ascc3 | up |
| rno-miR-411-5p | Sim1 | up |
| rno-miR-329-3p | Mef2a | up |
| rno-miR-329-3p | Ercc8 | up |
| rno-miR-329-3p | Depdc1b | up |
| rno-miR-133a-3p | Snx18 | up |
| rno-miR-133b-3p | Snx18 | up |
| rno-miR-411-5p | Sema5a | up |
| rno-miR-329-3p | Zfhx4 | up |
| rno-miR-206-3p | Lrrc31 | up |
| rno-miR-133a-5p | Samd7 | up |
| rno-miR-133a-5p | Phc3 | up |
| rno-miR-206-3p | Hps3 | up |
| rno-miR-675-3p | Usp13 | up |
| rno-miR-126a-3p | Ttc14 | up |
| rno-miR-133a-5p | Ttc14 | up |
| rno-miR-325-5p | Ttc14 | up |
| rno-miR-675-3p | Ttc14 | up |
| rno-miR-325-5p | Plcxd3 | up |
| rno-miR-325-5p | Fbxo4 | up |
| rno-miR-206-3p | Plch1 | up |
| rno-miR-329-3p | Plch1 | up |
| rno-miR-206-3p | Ppm1l | up |
| rno-miR-206-3p | Slitrk3 | up |
| rno-miR-329-3p | RGD1560010 | up |
| rno-miR-325-5p | Fam198b | up |
| rno-miR-133a-3p | Gatad2b | up |
| rno-miR-133b-3p | Gatad2b | up |
| rno-miR-206-3p | Iqgap3 | up |
| rno-miR-206-3p | Rfx5 | up |
| rno-miR-133a-3p | Prune | up |
| rno-miR-133b-3p | Prune | up |
| rno-miR-325-5p | Otud7b | up |
| rno-miR-411-5p | Otud7b | up |
| rno-miR-126a-3p | Polr3c | up |
| rno-miR-206-3p | Kirrel | up |
| rno-miR-325-5p | Slc22a15 | up |
| rno-miR-411-5p | Lrig2 | up |
| rno-miR-133a-3p | Fam212b | up |
| rno-miR-133b-3p | Fam212b | up |
| rno-miR-206-3p | Mybphl | up |
| rno-miR-411-5p | Mybphl | up |
| rno-miR-133a-3p | Slc30a7 | up |
| rno-miR-133b-3p | Slc30a7 | up |
| rno-miR-325-5p | Ptbp2 | up |
| rno-miR-329-3p | Ptbp2 | up |
| rno-miR-329-3p | Arhgap29 | up |
| rno-miR-133a-3p | Sgms2 | up |
| rno-miR-133b-3p | Sgms2 | up |
| rno-miR-411-5p | Gstcd | up |
| rno-miR-329-3p | Tet2 | up |
| rno-miR-133a-3p | Rap1gds1 | up |
| rno-miR-133b-3p | Rap1gds1 | up |
| rno-miR-206-3p | Fpgt | up |
| rno-miR-206-3p | Usp33 | up |
| rno-miR-325-5p | Ttll7 | up |
| rno-miR-206-3p | Cytip | up |
| rno-miR-325-5p | Tlk1 | up |
| rno-miR-133a-3p | Ttc30a1 | up |
| rno-miR-133b-3p | Ttc30a1 | up |
| rno-miR-206-3p | Ccdc141 | up |
| rno-miR-206-3p | Lrrc55 | up |
| rno-miR-325-5p | Lrrc55 | up |
| rno-miR-675-3p | Fnbp4 | up |
| rno-miR-675-3p | Kbtbd4 | up |
| rno-miR-329-3p | Qser1 | up |
| rno-miR-325-5p | Disp2 | up |
| rno-miR-329-3p | Ttbk2 | up |
| rno-miR-133a-5p | Ppip5k1 | up |
| rno-miR-206-3p | RGD1561442 | up |
| rno-miR-329-3p | Slc4a11 | up |
| rno-miR-206-3p | Siglec1 | up |
| rno-miR-325-5p | Rassf2 | up |
| rno-miR-329-3p | Rassf2 | up |
| rno-miR-133a-3p | Btbd3 | up |
| rno-miR-133b-3p | Btbd3 | up |
| rno-miR-329-3p | Btbd3 | up |
| rno-miR-133a-3p | Kif16b | up |
| rno-miR-133b-3p | Kif16b | up |
| rno-miR-206-3p | Rrbp1 | up |
| rno-miR-329-3p | Polr3f | up |
| rno-miR-675-3p | Rin2 | up |
| rno-miR-325-5p | Ninl | up |
| rno-miR-329-3p | Sdcbp2 | up |
| rno-miR-133a-3p | Ttll9 | up |
| rno-miR-133b-3p | Ttll9 | up |
| rno-miR-329-3p | RGD1305202 | up |
| rno-miR-133a-3p | Pofut1 | up |
| rno-miR-133a-5p | Pofut1 | up |
| rno-miR-133b-3p | Pofut1 | up |
| rno-miR-206-3p | Itch | up |
| rno-miR-133a-3p | Soga1 | up |
| rno-miR-133a-5p | Soga1 | up |
| rno-miR-133b-3p | Soga1 | up |
| rno-miR-329-3p | Samhd1 | up |
| rno-miR-133a-5p | Zhx3 | up |
| rno-miR-329-3p | Zhx3 | up |
| rno-miR-329-3p | Fitm2 | up |
| rno-miR-325-5p | Slc9a8 | up |
| rno-miR-329-3p | Nfatc2 | up |
| rno-miR-206-3p | Cstf1 | up |
| rno-miR-329-3p | Cstf1 | up |
| rno-miR-411-5p | Col20a1 | up |
| rno-miR-206-3p | Lrrc26 | up |
| rno-miR-325-5p | Rexo4 | up |
| rno-miR-206-3p | Lrsam1 | up |
| rno-miR-329-3p | Zbtb43 | up |
| rno-miR-675-3p | Zbtb26 | up |
| rno-miR-411-5p | Rabgap1 | up |
| rno-miR-206-3p | Rsbn1l | up |
| rno-miR-206-3p | Scaf11 | up |
| rno-miR-206-3p | Ccdc132 | up |
| rno-miR-325-5p | Ccdc132 | up |
| rno-miR-206-3p | Ophn1 | up |
| rno-miR-206-3p | Rbm28 | up |
| rno-miR-206-3p | Dennd2a | up |
| rno-miR-329-3p | Zfp775 | up |
| rno-miR-206-3p | Avl9 | up |
| rno-miR-329-3p | Ppm1k | up |
| rno-miR-329-3p | Paip2b | up |
| rno-miR-206-3p | Fbxo41 | up |
| rno-miR-206-3p | Zfyve20 | up |
| rno-miR-133a-5p | Rybp | up |
| rno-miR-329-3p | Atg7 | up |
| rno-miR-329-3p | Rasgef1a | up |
| rno-miR-133a-3p | Fbxl14 | up |
| rno-miR-133b-3p | Fbxl14 | up |
| rno-miR-325-5p | Il17ra | up |
| rno-miR-206-3p | Tspan9 | up |
| rno-miR-329-3p | Eps8 | up |
| rno-miR-675-3p | Gdap1 | up |
| rno-miR-133a-3p | Slco5a1 | up |
| rno-miR-133b-3p | Slco5a1 | up |
| rno-miR-133a-5p | Rb1cc1 | up |
| rno-miR-206-3p | Sox17 | up |
| rno-miR-329-3p | Tgs1 | up |
| rno-miR-206-3p | Impad1 | up |
| rno-miR-133a-3p | Ubxn2b | up |
| rno-miR-133b-3p | Ubxn2b | up |
| rno-miR-329-3p | Ubxn2b | up |
| rno-miR-329-3p | Sfn | up |
| rno-miR-329-3p | Tmem222 | up |
| rno-miR-133a-3p | Wasf2 | up |
| rno-miR-133b-3p | Wasf2 | up |
| rno-miR-325-5p | Wasf2 | up |
| rno-miR-329-3p | Wasf2 | up |
| rno-miR-329-3p | Csmd2 | up |
| rno-miR-133a-3p | RGD1559904 | up |
| rno-miR-329-3p | Fbxl4 | up |
| rno-miR-206-3p | RGD1562865 | up |
| rno-miR-329-3p | Pm20d2 | up |
| rno-miR-329-3p | Slc35a1 | up |
| rno-miR-675-3p | RGD1359108 | up |
| rno-miR-329-3p | Nfx1 | up |
| rno-miR-411-5p | Fsd1l | up |
| rno-miR-325-5p | Tbc1d2 | up |
| rno-miR-133a-3p | Ptprd | up |
| rno-miR-133b-3p | Ptprd | up |
| rno-miR-411-5p | Psip1 | up |
| rno-miR-206-3p | Acer2 | up |
| rno-miR-133a-3p | Hook1 | up |
| rno-miR-133b-3p | Hook1 | up |
| rno-miR-329-3p | Cyp2j3 | up |
| rno-miR-329-3p | Usp24 | up |
| rno-miR-133a-5p | Zfyve9 | up |
| rno-miR-329-3p | Atpaf1 | up |
| rno-miR-133a-3p | Hectd3 | up |
| rno-miR-133b-3p | Hectd3 | up |
| rno-miR-325-5p | Tmem53 | up |
| rno-miR-329-3p | Hivep3 | up |
| rno-miR-206-3p | Exo5 | up |
| rno-miR-411-5p | Heyl | up |
| rno-miR-325-5p | Col8a2 | up |
| rno-miR-325-5p | Ago1 | up |
| rno-miR-133a-5p | Zmym4 | up |
| rno-miR-133a-3p | Ddi2 | up |
| rno-miR-133b-3p | Ddi2 | up |
| rno-miR-329-3p | Ddi2 | up |
| rno-miR-329-3p | Kazn | up |
| rno-miR-329-3p | Ptchd2 | up |
| rno-miR-329-3p | Casz1 | up |
| rno-miR-133a-3p | Gpr157 | up |
| rno-miR-133b-3p | Gpr157 | up |
| rno-miR-329-3p | Phf13 | up |
| rno-miR-329-3p | Klhl21 | up |
| rno-miR-325-5p | Nol9 | up |
| rno-miR-329-3p | Nol9 | up |
| rno-miR-329-3p | Plch2 | up |
| rno-miR-133a-3p | Slc35e2b | up |
| rno-miR-133a-5p | Slc35e2b | up |
| rno-miR-133b-3p | Slc35e2b | up |
| rno-miR-133a-3p | RGD1311517 | up |
| rno-miR-206-3p | Lacc1 | up |
| rno-miR-325-5p | Prkd3 | up |
| rno-miR-329-3p | Prkd3 | up |
| rno-miR-329-3p | Eml4 | up |
| rno-miR-329-3p | Fam98a | up |
| rno-miR-329-3p | Rasgrp3 | up |
| rno-miR-329-3p | Ncoa1 | up |
| rno-miR-133a-5p | RGD1311648 | up |
| rno-miR-329-3p | Hs1bp3 | up |
| rno-miR-206-3p | Mboat2 | up |
| rno-miR-411-5p | Trappc12 | up |
| rno-miR-206-3p | Klhl15 | up |
| rno-miR-325-5p | Klhl15 | up |
| rno-miR-675-3p | Klhl15 | up |
| rno-miR-133a-5p | Insm2 | up |
| rno-miR-206-3p | Six6 | up |
| rno-miR-133a-3p | Sptb | up |
| rno-miR-133b-3p | Sptb | up |
| rno-miR-133a-3p | Rdh12 | up |
| rno-miR-133b-3p | Rdh12 | up |
| rno-miR-133a-3p | Dcaf5 | up |
| rno-miR-133b-3p | Dcaf5 | up |
| rno-miR-133a-3p | Smoc1 | up |
| rno-miR-133b-3p | Smoc1 | up |
| rno-miR-206-3p | Papln | up |
| rno-miR-206-3p | Gpatch2l | up |
| rno-miR-133a-5p | Cipc | up |
| rno-miR-411-5p | Tmem63c | up |
| rno-miR-206-3p | Ston2 | up |
| rno-miR-329-3p | Ston2 | up |
| rno-miR-329-3p | Sel1l | up |
| rno-miR-325-5p | Rps6ka5 | up |
| rno-miR-133a-3p | Gpr68 | up |
| rno-miR-133b-3p | Gpr68 | up |
| rno-miR-133a-3p | Trip11 | up |
| rno-miR-133b-3p | Trip11 | up |
| rno-miR-206-3p | Trip11 | up |
| rno-miR-133a-5p | Ubr7 | up |
| rno-miR-133a-3p | Unc79 | up |
| rno-miR-133b-3p | Unc79 | up |
| rno-miR-329-3p | Tmem179 | up |
| rno-miR-329-3p | Macc1 | up |
| rno-miR-329-3p | Tspyl5 | up |
| rno-miR-133a-3p | Celf5 | up |
| rno-miR-133b-3p | Celf5 | up |
| rno-miR-325-5p | Ric8b | up |
| rno-miR-411-5p | Kera | up |
| rno-miR-206-3p | E2f7 | up |
| rno-miR-329-3p | E2f7 | up |
| rno-miR-206-3p | Ptprb | up |
| rno-miR-325-5p | Ptprb | up |
| rno-miR-329-3p | Best3 | up |
| rno-miR-206-3p | Frs2 | up |
| rno-miR-329-3p | Frs2 | up |
| rno-miR-325-5p | Irak3 | up |
| rno-miR-329-3p | Srgap1 | up |
| rno-miR-133a-5p | Nab2 | up |
| rno-miR-133a-5p | Zfpm2 | up |
| rno-miR-133a-3p | Deptor | up |
| rno-miR-133b-3p | Deptor | up |
| rno-miR-206-3p | Deptor | up |
| rno-miR-411-5p | Col14a1 | up |
| rno-miR-133a-3p | Dennd3 | up |
| rno-miR-133b-3p | Dennd3 | up |
| rno-miR-329-3p | Tssk5 | up |
| rno-miR-133a-5p | Josd1 | up |
| rno-miR-206-3p | Cbx6 | up |
| rno-miR-133a-5p | Mief1 | up |
| rno-miR-133a-3p | Zc3h7b | up |
| rno-miR-133b-3p | Zc3h7b | up |
| rno-miR-329-3p | Scube1 | up |
| rno-miR-206-3p | Twf1 | up |
| rno-miR-329-3p | Ano6 | up |
| rno-miR-133a-3p | Ccnt1 | up |
| rno-miR-133b-3p | Ccnt1 | up |
| rno-miR-133a-3p | Nckap5l | up |
| rno-miR-133b-3p | Nckap5l | up |
| rno-miR-206-3p | Hoxc5 | up |
| rno-miR-675-3p | Hoxc5 | up |
| rno-miR-206-3p | Nxt2 | up |
| rno-miR-329-3p | Amotl1 | up |
| rno-miR-329-3p | Panx1 | up |
| rno-miR-329-3p | Tmed1 | up |
| rno-miR-325-5p | Snx19 | up |
| rno-miR-329-3p | Tbcel | up |
| rno-miR-329-3p | Ube4a | up |
| rno-miR-411-5p | Scn4b | up |
| rno-miR-133a-5p | Pih1d2 | up |
| rno-miR-329-3p | Pih1d2 | up |
| rno-miR-329-3p | RGD1311251 | up |
| rno-miR-329-3p | Npat | up |
| rno-miR-133a-3p | Peak1 | up |
| rno-miR-133b-3p | Peak1 | up |
| rno-miR-133a-5p | Snx33 | up |
| rno-miR-325-5p | Edc3 | up |
| rno-miR-206-3p | Pml | up |
| rno-miR-133a-5p | Thsd4 | up |
| rno-miR-329-3p | Paqr5 | up |
| rno-miR-206-3p | Dennd4a | up |
| rno-miR-329-3p | Tln2 | up |
| rno-miR-329-3p | Cgnl1 | up |
| rno-miR-133a-3p | Zfp280d | up |
| rno-miR-133b-3p | Zfp280d | up |
| rno-miR-206-3p | Klhl31 | up |
| rno-miR-329-3p | Phip | up |
| rno-miR-133a-3p | Prss35 | up |
| rno-miR-133b-3p | Prss35 | up |
| rno-miR-133a-3p | Zic4 | up |
| rno-miR-133b-3p | Zic4 | up |
| rno-miR-133a-3p | Plscr2 | up |
| rno-miR-133b-3p | Plscr2 | up |
| rno-miR-411-5p | U2surp | up |
| rno-miR-325-5p | Pxylp1 | up |
| rno-miR-411-5p | Pxylp1 | up |
| rno-miR-206-3p | Ky | up |
| rno-miR-325-5p | Nek11 | up |
| rno-miR-675-3p | Dock3 | up |
| rno-miR-133a-5p | Plxnb1 | up |
| rno-miR-206-3p | Epm2aip1 | up |
| rno-miR-675-3p | Limd1 | up |
| rno-miR-206-3p | Plin3 | up |
| rno-miR-206-3p | Tesl | up |
| rno-miR-206-3p | Daam2 | up |
| rno-miR-329-3p | Daam2 | up |
| rno-miR-133a-3p | Usp49 | up |
| rno-miR-133b-3p | Usp49 | up |
| rno-miR-329-3p | Usp49 | up |
| rno-miR-325-5p | Guca1b | up |
| rno-miR-411-5p | Trerf1 | up |
| rno-miR-133a-5p | Nfkbie | up |
| rno-miR-329-3p | Tcte1 | up |
| rno-miR-133a-5p | Zfp451 | up |
| rno-miR-411-5p | Zfp451 | up |
| rno-miR-133a-5p | Imp4 | up |
| rno-miR-133a-5p | Hs6st1 | up |
| rno-miR-206-3p | Actr1b | up |
| rno-miR-329-3p | Actr1b | up |
| rno-miR-133a-3p | Mfsd9 | up |
| rno-miR-133b-3p | Mfsd9 | up |
| rno-miR-329-3p | Hecw2 | up |
| rno-miR-411-5p | Hecw2 | up |
| rno-miR-133a-3p | Mars2 | up |
| rno-miR-133b-3p | Mars2 | up |
| rno-miR-133a-3p | Tmem237 | up |
| rno-miR-133b-3p | Tmem237 | up |
| rno-miR-206-3p | Tmem237 | up |
| rno-miR-411-5p | Aox4 | up |
| rno-miR-206-3p | Ino80d | up |
| rno-miR-206-3p | Plekhm3 | up |
| rno-miR-411-5p | Plekhm3 | up |
| rno-miR-325-5p | Pikfyve | up |
| rno-miR-675-3p | Wnt6 | up |
| rno-miR-329-3p | Csprs | up |
| rno-miR-133a-3p | Agap1 | up |
| rno-miR-133b-3p | Agap1 | up |
| rno-miR-329-3p | Agap1 | up |
| rno-miR-133a-3p | Sned1 | up |
| rno-miR-133b-3p | Sned1 | up |
| rno-miR-206-3p | Sned1 | up |
| rno-miR-325-5p | Fam114a1l1 | up |
| rno-miR-206-3p | Mtcl1 | up |
| rno-miR-133a-3p | Ankrd12 | up |
| rno-miR-133b-3p | Ankrd12 | up |
| rno-miR-411-5p | Mettl20 | up |
| rno-miR-133a-3p | Nhsl2 | up |
| rno-miR-133b-3p | Nhsl2 | up |
| rno-miR-206-3p | Nhsl2 | up |
| rno-miR-206-3p | Rpl7l1 | up |
| rno-miR-329-3p | Mtmr1 | up |
| rno-miR-206-3p | RGD1565685 | up |
| rno-miR-325-5p | Tfe3 | up |
| rno-miR-325-5p | Ccdc22 | up |
| rno-miR-206-3p | Shroom4 | up |
| rno-miR-325-5p | Ddx21 | up |
| rno-miR-206-3p | MGC109340 | up |
| rno-miR-329-3p | MGC109340 | up |
| rno-miR-206-3p | Wnk3 | up |
| rno-miR-325-5p | FAM120C | up |
| rno-miR-329-3p | Kdm5c | up |
| rno-miR-126a-3p | Ocrl | up |
| rno-miR-329-3p | Atp11c | up |
| rno-miR-411-5p | Yrdc | up |
| rno-miR-133a-3p | Sla | up |
| rno-miR-133b-3p | Sla | up |
| rno-miR-325-5p | Tnfsf9 | up |
| rno-miR-133a-5p | Myrip | up |
| rno-miR-325-5p | Zfp191 | up |
| rno-miR-411-5p | Slit2 | up |
| rno-miR-133a-3p | Ptprk | up |
| rno-miR-133b-3p | Ptprk | up |
| rno-miR-133a-3p | RT1-N2 | up |
| rno-miR-133b-3p | RT1-N2 | up |
| rno-miR-329-3p | Zc3h7a | up |
| rno-miR-329-3p | Usp7 | up |
| rno-miR-206-3p | Alg1 | up |
| rno-miR-411-5p | Cdip1 | up |
| rno-miR-329-3p | Dnaja3 | up |
| rno-miR-329-3p | Nprl3 | up |
| rno-miR-206-3p | RGD1311343 | up |
| rno-miR-325-5p | Akap10 | up |
| rno-miR-206-3p | Phf23 | up |
| rno-miR-206-3p | Bcl6b | up |
| rno-miR-133a-5p | Nlrp1a | up |
| rno-miR-206-3p | Rab34 | up |
| rno-miR-133a-3p | Ypel2 | up |
| rno-miR-133b-3p | Ypel2 | up |
| rno-miR-206-3p | Ypel2 | up |
| rno-miR-133a-3p | Prr11 | up |
| rno-miR-133b-3p | Prr11 | up |
| rno-miR-325-5p | Igfbp4 | up |
| rno-miR-329-3p | Cep131 | up |
| rno-miR-329-3p | Narf | up |
| rno-miR-206-3p | Wdr45b | up |
| rno-miR-325-5p | RGD1563888 | up |
| rno-miR-133a-5p | RGD1562683 | up |
| rno-miR-329-3p | RGD1310335 | up |
| rno-miR-329-3p | Rabl3 | up |
| rno-miR-133a-3p | Dgcr2 | up |
| rno-miR-133b-3p | Dgcr2 | up |
| rno-miR-675-3p | Dgcr2 | up |
| rno-miR-133a-3p | Pan3 | up |
| rno-miR-133b-3p | Pan3 | up |
| rno-miR-133a-3p | Zfand2a | up |
| rno-miR-133b-3p | Zfand2a | up |
| rno-miR-411-5p | RGD1563400 | up |
| rno-miR-126a-3p | Lrch4 | up |
| rno-miR-206-3p | Ap1s1 | up |
| rno-miR-133a-5p | Ankrd13a | up |
| rno-miR-329-3p | Vps4b | up |
| rno-miR-206-3p | Srgap2 | up |
| rno-miR-325-5p | Srgap2 | up |
| rno-miR-329-3p | Plekha6 | up |
| rno-miR-329-3p | Nek7 | up |
| rno-miR-411-5p | Rcsd1 | up |
| rno-miR-411-5p | Taf1a | up |
| rno-miR-329-3p | RGD1310587 | up |
| rno-miR-325-5p | Mtf2 | up |
| rno-miR-411-5p | Mtf2 | up |
| rno-miR-329-3p | Cdc7 | up |
| rno-miR-133a-3p | Tmem150c | up |
| rno-miR-133b-3p | Tmem150c | up |
| rno-miR-411-5p | Tmem150c | up |
| rno-miR-206-3p | Sel1l3 | up |
| rno-miR-133a-3p | Tmem128 | up |
| rno-miR-133b-3p | Tmem128 | up |
| rno-miR-206-3p | Tacc3 | up |
| rno-miR-133a-3p | Gas2l1 | up |
| rno-miR-133b-3p | Gas2l1 | up |
| rno-miR-206-3p | Gas2l1 | up |
| rno-miR-133a-5p | Fam107a | up |
| rno-miR-206-3p | Ktn1 | up |
| rno-miR-206-3p | Arhgef40 | up |
| rno-miR-329-3p | Xpo7 | up |
| rno-miR-206-3p | Dgkh | up |
| rno-miR-133a-5p | Zic2 | up |
| rno-miR-206-3p | RGD1309676 | up |
| rno-miR-206-3p | 1-Mar | up |
| rno-miR-206-3p | Purg | up |
| rno-miR-329-3p | Purg | up |
| rno-miR-133a-5p | Bag4 | up |
| rno-miR-133a-3p | Adam32 | up |
| rno-miR-133b-3p | Adam32 | up |
| rno-miR-133a-5p | Golga7 | up |
| rno-miR-329-3p | Golga7 | up |
| rno-miR-329-3p | Alg11 | up |
| rno-miR-329-3p | Usp19 | up |
| rno-miR-133a-5p | Ercc6l2 | up |
| rno-miR-325-5p | Rnf44 | up |
| rno-miR-133a-5p | Dhtkd1 | up |
| rno-miR-133a-3p | Plxdc2 | up |
| rno-miR-133b-3p | Plxdc2 | up |
| rno-miR-206-3p | Riok3 | up |
| rno-miR-206-3p | Ss18 | up |
| rno-miR-133a-3p | Rnf125 | up |
| rno-miR-133b-3p | Rnf125 | up |
| rno-miR-329-3p | Rnf125 | up |
| rno-miR-206-3p | Yipf5 | up |
| rno-miR-206-3p | Sema6a | up |
| rno-miR-133a-5p | Snx24 | up |
| rno-miR-411-5p | Fech | up |
| rno-miR-133a-5p | Phkb | up |
| rno-miR-133a-5p | Zfp330 | up |
| rno-miR-329-3p | Gab1 | up |
| rno-miR-329-3p | Abce1 | up |
| rno-miR-206-3p | Vat1l | up |
| rno-miR-329-3p | Fam92b | up |
| rno-miR-206-3p | Ust | up |
| rno-miR-206-3p | Echdc1 | up |
| rno-miR-206-3p | Tppp | up |
| rno-miR-206-3p | Eps8l1 | up |
| rno-miR-206-3p | Ube2m | up |
| rno-miR-411-5p | Hnrnpul1 | up |
| rno-miR-325-5p | U2af1l4 | up |
| rno-miR-206-3p | Lsm14a | up |
| rno-miR-206-3p | Vstm2b | up |
| rno-miR-133a-3p | Prr12 | up |
| rno-miR-133b-3p | Prr12 | up |
| rno-miR-133a-3p | Pcf11 | up |
| rno-miR-133b-3p | Pcf11 | up |
| rno-miR-675-3p | Pcf11 | up |
| rno-miR-329-3p | Fam168a | up |
| rno-miR-325-5p | Trim66 | up |
| rno-miR-133a-5p | Tead1 | up |
| rno-miR-325-5p | Ears2 | up |
| rno-miR-206-3p | Plekha1 | up |
| rno-miR-206-3p | Lhpp | up |
| rno-miR-206-3p | Ebf3 | up |
| rno-miR-206-3p | Pwwp2b | up |
| rno-miR-675-3p | Tnfrsf26 | up |
| rno-miR-675-3p | Mta2 | up |
| rno-miR-206-3p | Trub1 | up |
| rno-miR-206-3p | Srpk1 | up |
| rno-miR-133a-3p | Rnf8 | up |
| rno-miR-133b-3p | Rnf8 | up |
| rno-miR-325-5p | Zfand3 | up |
| rno-miR-329-3p | RGD1566085 | up |
| rno-miR-206-3p | RGD1306739 | up |
| rno-miR-325-5p | Spock2 | up |
| rno-miR-329-3p | Spock2 | up |
| rno-miR-133a-5p | Aifm2 | up |
| rno-miR-206-3p | Hace1 | up |
| rno-miR-206-3p | Cxadrl1 | up |
| rno-miR-329-3p | Ssbp2 | up |
| rno-miR-329-3p | Papd4 | up |
| rno-miR-133a-5p | Wdr41 | up |
| rno-miR-206-3p | Fbxl7 | up |
| rno-miR-329-3p | Fbxl7 | up |
| rno-miR-206-3p | Chmp4c | up |
| rno-miR-325-5p | Ythdf3 | up |
| rno-miR-133a-3p | Elf2 | up |
| rno-miR-133b-3p | Elf2 | up |
| rno-miR-206-3p | Supt20 | up |
| rno-miR-133a-3p | Lekr1 | up |
| rno-miR-133b-3p | Lekr1 | up |
| rno-miR-329-3p | Tmem144 | up |
| rno-miR-206-3p | Trim2 | up |
| rno-miR-329-3p | Them4 | up |
| rno-miR-325-5p | Prpf3 | up |
| rno-miR-329-3p | Sike1 | up |
| rno-miR-133a-3p | Ahcyl1 | up |
| rno-miR-133b-3p | Ahcyl1 | up |
| rno-miR-133a-5p | Ostc | up |
| rno-miR-329-3p | Unc5c | up |
| rno-miR-206-3p | Cryz | up |
| rno-miR-329-3p | Brd3 | up |
| rno-miR-206-3p | Aif1l | up |
| rno-miR-133a-5p | Mvb12b | up |
| rno-miR-133a-3p | Celf1 | up |
| rno-miR-133b-3p | Celf1 | up |
| rno-miR-325-5p | Celf1 | up |
| rno-miR-325-5p | Arfgap2 | up |
| rno-miR-206-3p | Atg13 | up |
| rno-miR-411-5p | Alkbh3 | up |
| rno-miR-325-5p | Rcn1 | up |
| rno-miR-206-3p | Slc5a12 | up |
| rno-miR-206-3p | Dph6 | up |
| rno-miR-675-3p | Nop56 | up |
| rno-miR-329-3p | Dtd1 | up |
| rno-miR-206-3p | Xrn2 | up |
| rno-miR-329-3p | Tbc1d20 | up |
| rno-miR-133a-3p | Dusp15 | up |
| rno-miR-133b-3p | Dusp15 | up |
| rno-miR-206-3p | Nol4l | up |
| rno-miR-133a-3p | Snhg11 | up |
| rno-miR-133b-3p | Snhg11 | up |
| rno-miR-206-3p | Ralgapb | up |
| rno-miR-329-3p | Dhx35 | up |
| rno-miR-206-3p | Ptprt | up |
| rno-miR-325-5p | Ptprt | up |
| rno-miR-206-3p | Rnf114 | up |
| rno-miR-329-3p | Rnf114 | up |
| rno-miR-133a-3p | Rbm33 | up |
| rno-miR-133a-5p | Rbm33 | up |
| rno-miR-133b-3p | Rbm33 | up |
| rno-miR-206-3p | Agap3 | up |
| rno-miR-206-3p | Cdk14 | up |
| rno-miR-325-5p | Slc25a13 | up |
| rno-miR-411-5p | Slc25a13 | up |
| rno-miR-206-3p | Mdfic | up |
| rno-miR-329-3p | Mdfic | up |
| rno-miR-329-3p | Hipk2 | up |
| rno-miR-133a-3p | Fam115a | up |
| rno-miR-133b-3p | Fam115a | up |
| rno-miR-325-5p | Cul1 | up |
| rno-miR-675-3p | Mpp6 | up |
| rno-miR-206-3p | Herc6 | up |
| rno-miR-411-5p | Reep1 | up |
| rno-miR-133a-5p | Mxd1 | up |
| rno-miR-133a-5p | Clec4a1 | up |
| rno-miR-411-5p | Loh12cr1 | up |
| rno-miR-329-3p | Crebl2 | up |
| rno-miR-133a-3p | Tmtc1 | up |
| rno-miR-133b-3p | Tmtc1 | up |
| rno-miR-133a-5p | Bicd1 | up |
| rno-miR-325-5p | Ndufaf4 | up |
| rno-miR-329-3p | Shb | up |
| rno-miR-133a-3p | Fktn | up |
| rno-miR-133b-3p | Fktn | up |
| rno-miR-411-5p | Fktn | up |
| rno-miR-329-3p | Zfp462 | up |
| rno-miR-206-3p | Ptpn3 | up |
| rno-miR-133a-3p | Fkbp15 | up |
| rno-miR-133b-3p | Fkbp15 | up |
| rno-miR-325-5p | Ptplad2 | up |
| rno-miR-411-5p | Ptplad2 | up |
| rno-miR-133a-5p | Ttc4 | up |
| rno-miR-329-3p | Ttc4 | up |
| rno-miR-329-3p | Zyg11b | up |
| rno-miR-133a-3p | Mtf1 | up |
| rno-miR-133a-5p | Mtf1 | up |
| rno-miR-133b-3p | Mtf1 | up |
| rno-miR-206-3p | Sh3d21 | up |
| rno-miR-206-3p | Trappc3 | up |
| rno-miR-325-5p | Sync | up |
| rno-miR-133a-3p | Gpn2 | up |
| rno-miR-133b-3p | Gpn2 | up |
| rno-miR-675-3p | Man1c1 | up |
| rno-miR-325-5p | Srsf10 | up |
| rno-miR-133a-5p | Zbtb40 | up |
| rno-miR-329-3p | Mthfr | up |
| rno-miR-329-3p | Camta1 | up |
| rno-miR-329-3p | Lrrc47 | up |
| rno-miR-133a-5p | Pusl1 | up |
| rno-miR-206-3p | Heatr5b | up |
| rno-miR-206-3p | Cebpz | up |
| rno-miR-206-3p | Tmem178a | up |
| rno-miR-206-3p | Tmem18 | up |
| rno-miR-329-3p | Tmem18 | up |
| rno-miR-329-3p | Atxn7l4 | up |
| rno-miR-325-5p | Stxbp6 | up |
| rno-miR-206-3p | Tmx1 | up |
| rno-miR-329-3p | Angel1 | up |
| rno-miR-329-3p | Alkbh1 | up |
| rno-miR-206-3p | Ttc7b | up |
| rno-miR-133a-5p | Btbd7 | up |
| rno-miR-206-3p | Btbd7 | up |
| rno-miR-325-5p | Hhipl1 | up |
| rno-miR-675-3p | Traf3 | up |
| rno-miR-206-3p | Zfyve21 | up |
| rno-miR-329-3p | Ncapg2 | up |
| rno-miR-206-3p | Nabp2 | up |
| rno-miR-126a-3p | Sarnp | up |
| rno-miR-133a-3p | Mfsd12 | up |
| rno-miR-133b-3p | Mfsd12 | up |
| rno-miR-675-3p | Atcay | up |
| rno-miR-133a-3p | Sppl2b | up |
| rno-miR-133b-3p | Sppl2b | up |
| rno-miR-133a-3p | Dot1l | up |
| rno-miR-133b-3p | Dot1l | up |
| rno-miR-329-3p | Mum1 | up |
| rno-miR-206-3p | Gnptab | up |
| rno-miR-329-3p | Chpt1 | up |
| rno-miR-133a-5p | Plxnc1 | up |
| rno-miR-133a-5p | Arhgap9 | up |
| rno-miR-206-3p | Rnf19a | up |
| rno-miR-329-3p | St3gal1 | up |
| rno-miR-329-3p | Ly6e | up |
| rno-miR-329-3p | Ly6al | up |
| rno-miR-133a-5p | Ankrd54 | up |
| rno-miR-329-3p | Rangap1 | up |
| rno-miR-133a-3p | Mpped1 | up |
| rno-miR-329-3p | Parvb | up |
| rno-miR-133a-5p | Tubgcp6 | up |
| rno-miR-133a-5p | Dennd6b | up |
| rno-miR-206-3p | Uhrf1bp1l | up |
| rno-miR-325-5p | Endod1 | up |
| rno-miR-329-3p | Slc44a2 | up |
| rno-miR-133a-3p | Spc24 | up |
| rno-miR-133b-3p | Spc24 | up |
| rno-miR-675-3p | RGD1307500 | up |
| rno-miR-411-5p | Dixdc1 | up |
| rno-miR-329-3p | Sin3a | up |
| rno-miR-675-3p | Car12 | up |
| rno-miR-325-5p | Fam63b | up |
| rno-miR-675-3p | Slc17a5 | up |
| rno-miR-329-3p | Nmnat3 | up |
| rno-miR-206-3p | Ppp2r3a | up |
| rno-miR-325-5p | Rad54l2 | up |
| rno-miR-329-3p | Rad54l2 | up |
| rno-miR-329-3p | Wdr48 | up |
| rno-miR-206-3p | Ulk4 | up |
| rno-miR-675-3p | Zfp167 | up |
| rno-miR-133a-3p | Klhdc3 | up |
| rno-miR-133b-3p | Klhdc3 | up |
| rno-miR-206-3p | Phf3 | up |
| rno-miR-411-5p | Rnf149 | up |
| rno-miR-411-5p | Tmeff2 | up |
| rno-miR-329-3p | Als2 | up |
| rno-miR-133a-3p | Raph1 | up |
| rno-miR-133b-3p | Raph1 | up |
| rno-miR-133a-5p | Klf7 | up |
| rno-miR-675-3p | Atg9a | up |
| rno-miR-325-5p | Agfg1 | up |
| rno-miR-329-3p | Cops7b | up |
| rno-miR-411-5p | Scly | up |
| rno-miR-206-3p | Vmac | up |
| rno-miR-411-5p | Reps2 | up |
| rno-miR-206-3p | Mid2 | up |
| rno-miR-133a-5p | Ids | up |
| rno-miR-325-5p | Irak1 | up |
| rno-miR-133a-3p | Naa60 | up |
| rno-miR-133b-3p | Naa60 | up |
| rno-miR-206-3p | Havcr2 | up |
| rno-miR-325-5p | RGD1560464 | up |
| rno-miR-206-3p | Tcf7 | up |
| rno-miR-325-5p | Tcf7 | up |
| rno-miR-133a-5p | Natd1 | up |
| rno-miR-325-5p | Rnf222 | up |
| rno-miR-329-3p | Mrm1 | up |
| rno-miR-133a-5p | Meox1 | up |
| rno-miR-329-3p | Krtap7-1 | up |
| rno-miR-133a-3p | Lmln | up |
| rno-miR-133b-3p | Lmln | up |
| rno-miR-329-3p | Hira | up |
| rno-miR-329-3p | Map2k7 | up |
| rno-miR-675-3p | Ubl3 | up |
| rno-miR-206-3p | Fzd10 | up |
| rno-miR-133a-5p | Tnfsf18 | up |
| rno-miR-411-5p | Slamf7 | up |
| rno-miR-411-5p | Irf6 | up |
| rno-miR-206-3p | Tmem55b | up |
| rno-miR-329-3p | Rnase9 | up |
| rno-miR-329-3p | Poteg | up |
| rno-miR-329-3p | Rnf144b | up |
| rno-miR-675-3p | Serpinb6b | up |
| rno-miR-133a-3p | Foxc1 | up |
| rno-miR-133b-3p | Foxc1 | up |
| rno-miR-329-3p | Foxf2 | up |
| rno-miR-133a-3p | Fzd8 | up |
| rno-miR-133b-3p | Fzd8 | up |
| rno-miR-329-3p | Calml5 | up |
| rno-miR-325-5p | Suv39h2 | up |
| rno-miR-325-5p | Itga8 | up |
| rno-miR-206-3p | Reep5 | up |
| rno-miR-133a-3p | Pcdhga8 | up |
| rno-miR-133b-3p | Pcdhga8 | up |
| rno-miR-133a-3p | Pcdhgb7 | up |
| rno-miR-133b-3p | Pcdhgb7 | up |
| rno-miR-133a-3p | Pcdhgb8 | up |
| rno-miR-133b-3p | Pcdhgb8 | up |
| rno-miR-206-3p | St8sia5 | up |
| rno-miR-325-5p | Asphd2 | up |
| rno-miR-329-3p | Scoc | up |
| rno-miR-325-5p | Rnf150 | up |
| rno-miR-329-3p | Rnf150 | up |
| rno-miR-206-3p | Sash1 | up |
| rno-miR-411-5p | Jakmip3 | up |
| rno-miR-133a-3p | Cpsf7 | up |
| rno-miR-133b-3p | Cpsf7 | up |
| rno-miR-329-3p | Cpsf7 | up |
| rno-miR-675-3p | Cpsf7 | up |
| rno-miR-133a-3p | Cfap43 | up |
| rno-miR-133b-3p | Cfap43 | up |
| rno-miR-206-3p | Cfap43 | up |
| rno-miR-329-3p | Cfap43 | up |
| rno-miR-325-5p | Cacul1 | up |
| rno-miR-325-5p | Nanos1 | up |
| rno-miR-329-3p | Col4a3bp | up |
| rno-miR-206-3p | Bhlhe22 | up |
| rno-miR-329-3p | Tbl1xr1 | up |
| rno-miR-411-5p | Tbl1xr1 | up |
| rno-miR-206-3p | Hipk1 | up |
| rno-miR-329-3p | Tmem56 | up |
| rno-miR-329-3p | Crb2 | up |
| rno-miR-325-5p | Nutm1 | up |
| rno-miR-133a-5p | Rtf1 | up |
| rno-miR-329-3p | Slc2a10 | up |
| rno-miR-133a-3p | Clvs1 | up |
| rno-miR-133b-3p | Clvs1 | up |
| rno-miR-206-3p | Faxc | up |
| rno-miR-329-3p | Mob3b | up |
| rno-miR-329-3p | RGD1309821 | up |
| rno-miR-133a-5p | Btf3l4 | up |
| rno-miR-325-5p | Col16a1 | up |
| rno-miR-206-3p | Epha2 | up |
| rno-miR-133a-3p | Pik3cd | up |
| rno-miR-133b-3p | Pik3cd | up |
| rno-miR-133a-5p | Atp6v1e2 | up |
| rno-miR-411-5p | Sypl1 | up |
| rno-miR-329-3p | Rbm25 | up |
| rno-miR-329-3p | Diras1 | up |
| rno-miR-206-3p | Trhde | up |
| rno-miR-206-3p | Tcf20 | up |
| rno-miR-206-3p | Pphln1 | up |
| rno-miR-206-3p | Zfp26 | up |
| rno-miR-206-3p | Ccdc15 | up |
| rno-miR-206-3p | Alg9 | up |
| rno-miR-411-5p | Hmgcll1 | up |
| rno-miR-133a-3p | Map4 | up |
| rno-miR-133b-3p | Map4 | up |
| rno-miR-325-5p | Map4 | up |
| rno-miR-329-3p | Map4 | up |
| rno-miR-206-3p | Mgat4a | up |
| rno-miR-325-5p | Kansl1l | up |
| rno-miR-675-3p | Kcne4 | up |
| rno-miR-325-5p | Alppl2 | up |
| rno-miR-133a-5p | Gpr35 | up |
| rno-miR-206-3p | Gpr35 | up |
| rno-miR-206-3p | Nudt12 | up |
| rno-miR-206-3p | MGC116197 | up |
| rno-miR-675-3p | Rp2 | up |
| rno-miR-133a-3p | Tmem164 | up |
| rno-miR-133b-3p | Tmem164 | up |
| rno-miR-325-5p | Scml2 | up |
| rno-miR-133a-5p | Tceal8 | up |
| rno-miR-329-3p | Tceal8 | up |
| rno-miR-206-3p | Ankib1 | up |
| rno-miR-329-3p | Hpse2 | up |
| rno-miR-133a-3p | Ermp1 | up |
| rno-miR-133b-3p | Ermp1 | up |
| rno-miR-206-3p | Ermp1 | up |
| rno-miR-206-3p | Trmt11 | up |
| rno-miR-329-3p | E2f1 | up |
| rno-miR-325-5p | Notch4 | up |
| rno-miR-329-3p | Pbx2 | up |
| rno-miR-675-3p | Btla | up |
| rno-miR-133a-5p | St6galnac4 | up |
| rno-miR-206-3p | Pan2 | up |
| rno-miR-206-3p | Elavl4 | up |
| rno-miR-329-3p | Apold1 | up |
| rno-miR-206-3p | Dnmt3a | up |
| rno-miR-206-3p | Il22ra2 | up |
| rno-miR-206-3p | Rbm4b | up |
| rno-miR-133a-5p | Gimap9 | up |
| rno-miR-329-3p | Oas3 | up |
| rno-miR-206-3p | Eogt | up |
| rno-miR-206-3p | Bin2a | up |
| rno-miR-206-3p | Kctd10 | up |
| rno-miR-133a-5p | Scn3a | up |
| rno-miR-133a-5p | Lmo3 | up |
| rno-miR-675-3p | Nrcam | up |
| rno-miR-329-3p | Dexi | up |
| rno-miR-329-3p | Atf7ip2 | up |
| rno-miR-133a-5p | Tmem186 | up |
| rno-miR-329-3p | Zfp286a | up |
| rno-miR-133a-5p | Trpv3 | up |
| rno-miR-206-3p | Trpv3 | up |
| rno-miR-325-5p | Proca1 | up |
| rno-miR-206-3p | Fam222b | up |
| rno-miR-329-3p | Nlk | up |
| rno-miR-206-3p | Lyrm9 | up |
| rno-miR-133a-3p | Fam117a | up |
| rno-miR-133b-3p | Fam117a | up |
| rno-miR-206-3p | RGD1565533 | up |
| rno-miR-133a-3p | Mrc2 | up |
| rno-miR-133b-3p | Mrc2 | up |
| rno-miR-206-3p | Pitpnc1 | up |
| rno-miR-329-3p | Pitpnc1 | up |
| rno-miR-133a-3p | Kctd2 | up |
| rno-miR-133b-3p | Kctd2 | up |
| rno-miR-206-3p | Anapc11 | up |
| rno-miR-206-3p | Vgll3 | up |
| rno-miR-206-3p | RGD1562726 | up |
| rno-miR-329-3p | Dtx3l | up |
| rno-miR-411-5p | Gmnc | up |
| rno-miR-329-3p | Stard13 | up |
| rno-miR-411-5p | RGD1561730 | up |
| rno-miR-325-5p | Fam163a | up |
| rno-miR-329-3p | Fcrlb | up |
| rno-miR-133a-3p | Arhgap30 | up |
| rno-miR-133b-3p | Arhgap30 | up |
| rno-miR-329-3p | Cnst | up |
| rno-miR-329-3p | Bmp2k | up |
| rno-miR-329-3p | Lcorl | up |
| rno-miR-133a-3p | Zfp518b | up |
| rno-miR-133b-3p | Zfp518b | up |
| rno-miR-206-3p | Zfp518b | up |
| rno-miR-206-3p | Cytl1 | up |
| rno-miR-329-3p | Msantd1 | up |
| rno-miR-325-5p | Sun3 | up |
| rno-miR-325-5p | Grb10 | up |
| rno-miR-133a-5p | Naa30 | up |
| rno-miR-325-5p | Khnyn | up |
| rno-miR-206-3p | Akap11 | up |
| rno-miR-329-3p | Akap11 | up |
| rno-miR-329-3p | Il17rd | up |
| rno-miR-329-3p | Ldb3 | up |
| rno-miR-206-3p | Fam129c | up |
| rno-miR-325-5p | Rbpms | up |
| rno-miR-206-3p | Fam155a | up |
| rno-miR-329-3p | Fam155a | up |
| rno-miR-329-3p | Ccdc71 | up |
| rno-miR-325-5p | Aaed1 | up |
| rno-miR-329-3p | RGD1566359 | up |
| rno-miR-329-3p | Nol7 | up |
| rno-miR-206-3p | Elovl2 | up |
| rno-miR-206-3p | Tdp2 | up |
| rno-miR-206-3p | Ccdc3 | up |
| rno-miR-126a-3p | Otud1 | up |
| rno-miR-206-3p | Ankrd29 | up |
| rno-miR-133a-3p | Pcdhga2 | up |
| rno-miR-133b-3p | Pcdhga2 | up |
| rno-miR-133a-3p | Pcdhga3 | up |
| rno-miR-133b-3p | Pcdhga3 | up |
| rno-miR-133a-3p | Pcdhga12 | up |
| rno-miR-133b-3p | Pcdhga12 | up |
| rno-miR-329-3p | RGD1560813 | up |
| rno-miR-329-3p | Arl2bp | up |
| rno-miR-675-3p | Lsm6 | up |
| rno-miR-133a-3p | Il34 | up |
| rno-miR-133b-3p | Il34 | up |
| rno-miR-206-3p | Il34 | up |
| rno-miR-325-5p | Il34 | up |
| rno-miR-329-3p | Acsf3 | up |
| rno-miR-325-5p | Themis | up |
| rno-miR-206-3p | Tmem200a | up |
| rno-miR-329-3p | Wtap | up |
| rno-miR-411-5p | Znf761 | up |
| rno-miR-206-3p | Nlrp4 | up |
| rno-miR-133a-5p | Zfp780b | up |
| rno-miR-206-3p | Zfp14 | up |
| rno-miR-329-3p | Spib | up |
| rno-miR-206-3p | Gas2 | up |
| rno-miR-325-5p | Svip | up |
| rno-miR-133a-5p | Knop1 | up |
| rno-miR-411-5p | Prss53 | up |
| rno-miR-329-3p | LOC499339 | up |
| rno-miR-411-5p | LOC499339 | up |
| rno-miR-133a-3p | Icoslg | up |
| rno-miR-133b-3p | Icoslg | up |
| rno-miR-206-3p | Icoslg | up |
| rno-miR-329-3p | RGD1564149 | up |
| rno-miR-329-3p | Gstt3 | up |
| rno-miR-133a-3p | Mcm9 | up |
| rno-miR-133b-3p | Mcm9 | up |
| rno-miR-329-3p | Ccdc138 | up |
| rno-miR-329-3p | Mef2c | up |
| rno-miR-675-3p | Mef2c | up |
| rno-miR-206-3p | Ankrd34b | up |
| rno-miR-675-3p | Naaladl2 | up |
| rno-miR-133a-5p | Gpr160 | up |
| rno-miR-206-3p | Gpr160 | up |
| rno-miR-325-5p | Cks1b | up |
| rno-miR-411-5p | Lix1l | up |
| rno-miR-329-3p | Dnajb14 | up |
| rno-miR-133a-5p | RGD1564379 | up |
| rno-miR-329-3p | RGD1564379 | up |
| rno-miR-133a-3p | Tbc1d13 | up |
| rno-miR-133b-3p | Tbc1d13 | up |
| rno-miR-133a-3p | LOC499770 | up |
| rno-miR-133b-3p | LOC499770 | up |
| rno-miR-675-3p | Dcaf17 | up |
| rno-miR-206-3p | Tp53tg5 | up |
| rno-miR-329-3p | Lhfpl3 | up |
| rno-miR-411-5p | Phf14 | up |
| rno-miR-325-5p | Lrrc61 | up |
| rno-miR-329-3p | Lrrc61 | up |
| rno-miR-325-5p | Ccdc126 | up |
| rno-miR-133a-5p | Creb5 | up |
| rno-miR-206-3p | RGD1560028 | up |
| rno-miR-133a-3p | Emx1 | up |
| rno-miR-133b-3p | Emx1 | up |
| rno-miR-206-3p | Aak1 | up |
| rno-miR-325-5p | Lrrn1 | up |
| rno-miR-675-3p | Arl8b | up |
| rno-miR-675-3p | Srgap3 | up |
| rno-miR-133a-3p | Clec1a | up |
| rno-miR-133b-3p | Clec1a | up |
| rno-miR-325-5p | Klrc3 | up |
| rno-miR-329-3p | Styk1 | up |
| rno-miR-206-3p | RGD1560652 | up |
| rno-miR-133a-5p | Far2 | up |
| rno-miR-325-5p | Fam110b | up |
| rno-miR-411-5p | Rmdn1 | up |
| rno-miR-133a-3p | RGD1559864 | up |
| rno-miR-133b-3p | RGD1559864 | up |
| rno-miR-206-3p | Caap1 | up |
| rno-miR-206-3p | Eqtn | up |
| rno-miR-329-3p | Eqtn | up |
| rno-miR-206-3p | RGD1559493 | up |
| rno-miR-133a-3p | Rnf220 | up |
| rno-miR-133b-3p | Rnf220 | up |
| rno-miR-206-3p | Zbtb8b | up |
| rno-miR-329-3p | Lin28a | up |
| rno-miR-133a-5p | Ldlrap1 | up |
| rno-miR-133a-3p | Tmco4 | up |
| rno-miR-133b-3p | Tmco4 | up |
| rno-miR-206-3p | Arhgef33 | up |
| rno-miR-675-3p | Dcdc2c | up |
| rno-miR-206-3p | Fam110c | up |
| rno-miR-325-5p | Dact1 | up |
| rno-miR-325-5p | Ccdc177 | up |
| rno-miR-206-3p | Cactin | up |
| rno-miR-206-3p | Rspo2 | up |
| rno-miR-325-5p | Rspo2 | up |
| rno-miR-675-3p | Rspo2 | up |
| rno-miR-133a-3p | Mtbp | up |
| rno-miR-133b-3p | Mtbp | up |
| rno-miR-325-5p | Mroh5 | up |
| rno-miR-411-5p | Ccdc134 | up |
| rno-miR-133a-3p | RGD1566029 | up |
| rno-miR-133b-3p | RGD1566029 | up |
| rno-miR-329-3p | RGD1566029 | up |
| rno-miR-675-3p | RGD1566029 | up |
| rno-miR-206-3p | Arhgap42 | up |
| rno-miR-329-3p | LOC500956 | up |
| rno-miR-206-3p | Stt3a | up |
| rno-miR-206-3p | Zfp202 | up |
| rno-miR-206-3p | Cbl | up |
| rno-miR-329-3p | Cbl | up |
| rno-miR-133a-5p | Ddx6 | up |
| rno-miR-133a-3p | Cplx3 | up |
| rno-miR-133b-3p | Cplx3 | up |
| rno-miR-206-3p | RGD1560775 | up |
| rno-miR-206-3p | Slc25a36 | up |
| rno-miR-133a-3p | Kif9 | up |
| rno-miR-133b-3p | Kif9 | up |
| rno-miR-325-5p | Kif9 | up |
| rno-miR-206-3p | Tbc1d5 | up |
| rno-miR-133a-5p | Ankrd66 | up |
| rno-miR-206-3p | Boll | up |
| rno-miR-133a-5p | RGD1562029 | up |
| rno-miR-325-5p | Zdbf2 | up |
| rno-miR-411-5p | Spag16 | up |
| rno-miR-133a-3p | Gpr55 | up |
| rno-miR-133b-3p | Gpr55 | up |
| rno-miR-206-3p | Rbm44 | up |
| rno-miR-325-5p | Rbm44 | up |
| rno-miR-411-5p | Ppidl1 | up |
| rno-miR-133a-5p | Sh2d1a | up |
| rno-miR-329-3p | Rps6ka3 | up |
| rno-miR-329-3p | Prkx | up |
| rno-miR-133a-5p | Tceal3 | up |
| rno-miR-329-3p | Vma21 | up |
| rno-miR-133a-5p | Lsm11 | up |
| rno-miR-206-3p | Lsm11 | up |
| rno-miR-329-3p | Lsm11 | up |
| rno-miR-329-3p | Cenpv | up |
| rno-miR-329-3p | Cd209e | up |
| rno-miR-206-3p | Coro1c | up |
| rno-miR-133a-3p | Rab7b | up |
| rno-miR-133b-3p | Rab7b | up |
| rno-miR-206-3p | Rab7b | up |
| rno-miR-206-3p | Sh2d1b | up |
| rno-miR-133a-3p | Slc2a9 | up |
| rno-miR-133b-3p | Slc2a9 | up |
| rno-miR-329-3p | Slc2a9 | up |
| rno-miR-325-5p | LOC501934 | up |
| rno-miR-206-3p | Lrch1 | up |
| rno-miR-329-3p | Lrch1 | up |
| rno-miR-133a-3p | Rnf122 | up |
| rno-miR-133b-3p | Rnf122 | up |
| rno-miR-329-3p | Pom121l2 | up |
| rno-miR-329-3p | Slc38a8 | up |
| rno-miR-206-3p | Slc35f1 | up |
| rno-miR-329-3p | RGD1564854 | up |
| rno-miR-133a-5p | Dstn | up |
| rno-miR-206-3p | Kmt2c | up |
| rno-miR-206-3p | Cpa4 | up |
| rno-miR-325-5p | Agk | up |
| rno-miR-325-5p | Scrn1 | up |
| rno-miR-325-5p | Foxj2 | up |
| rno-miR-133a-5p | Clec9a | up |
| rno-miR-329-3p | Clec9a | up |
| rno-miR-329-3p | Eya1 | up |
| rno-miR-206-3p | RGD1560884 | up |
| rno-miR-329-3p | Angptl3 | up |
| rno-miR-133a-5p | Nipal3 | up |
| rno-miR-133a-5p | Ctnnbip1 | up |
| rno-miR-325-5p | Ctnnbip1 | up |
| rno-miR-206-3p | Glb1l2 | up |
| rno-miR-206-3p | Sowahc | up |
| rno-miR-133a-5p | Gja5 | up |
| rno-miR-411-5p | Galr1 | up |
| rno-miR-329-3p | Slc23a1 | up |
| rno-miR-329-3p | Slc23a2 | up |
| rno-miR-133a-3p | Rab27a | up |
| rno-miR-133b-3p | Rab27a | up |
| rno-miR-329-3p | Mapk9 | up |
| rno-miR-206-3p | Runx1 | up |
| rno-miR-329-3p | Calm2 | up |
| rno-miR-329-3p | Gnao1 | up |
| rno-miR-206-3p | Pde3a | up |
| rno-miR-133a-3p | Slc7a5 | up |
| rno-miR-133b-3p | Slc7a5 | up |
| rno-miR-133a-3p | Hpcal4 | up |
| rno-miR-133b-3p | Hpcal4 | up |
| rno-miR-206-3p | Coil | up |
| rno-miR-206-3p | Zfp260 | up |
| rno-miR-206-3p | Sprn | up |
| rno-miR-206-3p | Cltc | up |
| rno-miR-329-3p | Cltc | up |
| rno-miR-325-5p | Crk | up |
| rno-miR-329-3p | Crk | up |
| rno-miR-206-3p | Mid1 | up |
| rno-miR-329-3p | Mid1 | up |
| rno-miR-329-3p | Llgl1 | up |
| rno-miR-206-3p | Neurod2 | up |
| rno-miR-133a-3p | Nr4a2 | up |
| rno-miR-133b-3p | Nr4a2 | up |
| rno-miR-206-3p | Nr4a2 | up |
| rno-miR-133a-3p | Furin | up |
| rno-miR-133b-3p | Furin | up |
| rno-miR-133a-3p | Rgs3 | up |
| rno-miR-133b-3p | Rgs3 | up |
| rno-miR-329-3p | Rgs3 | up |
| rno-miR-325-5p | Slc12a3 | up |
| rno-miR-206-3p | Cnn3 | up |
| rno-miR-133a-3p | Pcdhga1 | up |
| rno-miR-133b-3p | Pcdhga1 | up |
| rno-miR-206-3p | Ywhab | up |
| rno-miR-133a-5p | Tgm2 | up |
| rno-miR-325-5p | Cript | up |
| rno-miR-411-5p | Cript | up |
| rno-miR-206-3p | Srek1 | up |
| rno-miR-329-3p | Pth1r | up |
| rno-miR-133a-3p | Kcnip2 | up |
| rno-miR-133b-3p | Kcnip2 | up |
| rno-miR-329-3p | Adam17 | up |
| rno-miR-329-3p | Zfp148 | up |
| rno-miR-133a-5p | Zranb2 | up |
| rno-miR-133a-5p | Nr1h2 | up |
| rno-miR-329-3p | Ccnd1 | up |
| rno-miR-329-3p | Foxm1 | up |
| rno-miR-675-3p | Fut2 | up |
| rno-miR-133a-3p | Sult4a1 | up |
| rno-miR-133b-3p | Sult4a1 | up |
| rno-miR-325-5p | Nup210 | up |
| rno-miR-329-3p | Map2k2 | up |
| rno-miR-329-3p | Klrg1 | up |
| rno-miR-325-5p | Ppp1r1a | up |
| rno-miR-329-3p | Ncan | up |
| rno-miR-325-5p | Stx8 | up |
| rno-miR-411-5p | Stx8 | up |
| rno-miR-206-3p | Grk5 | up |
| rno-miR-329-3p | Ptges | up |
| rno-miR-133a-3p | Aipl1 | up |
| rno-miR-133b-3p | Aipl1 | up |
| rno-miR-206-3p | Ip6k2 | up |
| rno-miR-206-3p | Tcam1 | up |
| rno-miR-411-5p | Tcam1 | up |
| rno-miR-329-3p | Shank3 | up |
| rno-miR-206-3p | Kcnk1 | up |
| rno-miR-133a-3p | Twist2 | up |
| rno-miR-133a-5p | Twist2 | up |
| rno-miR-133b-3p | Twist2 | up |
| rno-miR-133a-3p | Arpp19 | up |
| rno-miR-133a-5p | Arpp19 | up |
| rno-miR-133b-3p | Arpp19 | up |
| rno-miR-675-3p | Arpp19 | up |
| rno-miR-206-3p | Npff | up |
| rno-miR-133a-3p | Camkk1 | up |
| rno-miR-133b-3p | Camkk1 | up |
| rno-miR-206-3p | Cdkl3 | up |
| rno-miR-133a-3p | Vapb | up |
| rno-miR-133b-3p | Vapb | up |
| rno-miR-411-5p | Rfng | up |
| rno-miR-133a-3p | Bcl2l2 | up |
| rno-miR-133b-3p | Bcl2l2 | up |
| rno-miR-675-3p | Bcl2l2 | up |
| rno-miR-133a-5p | Clock | up |
| rno-miR-206-3p | Clock | up |
| rno-miR-329-3p | Clock | up |
| rno-miR-206-3p | Stx6 | up |
| rno-miR-411-5p | Syt9 | up |
| rno-miR-133a-3p | Lif | up |
| rno-miR-133b-3p | Lif | up |
| rno-miR-411-5p | Clcn4 | up |
| rno-miR-133a-5p | Epha8 | up |
| rno-miR-411-5p | Epha8 | up |
| rno-miR-133a-3p | Max | up |
| rno-miR-133b-3p | Max | up |
| rno-miR-206-3p | Pik3r3 | up |
| rno-miR-329-3p | Cxcl6 | up |
| rno-miR-329-3p | Cmklr1 | up |
| rno-miR-133a-5p | Surf4 | up |
| rno-miR-206-3p | Surf4 | up |
| rno-miR-206-3p | Jam2 | up |
| rno-miR-329-3p | Acap2 | up |
| rno-miR-325-5p | Ncoa4 | up |
| rno-miR-325-5p | LOC619574 | up |
| rno-miR-411-5p | Nrbp1 | up |
| rno-miR-325-5p | Per2 | up |
| rno-miR-411-5p | P2ry4 | up |
| rno-miR-206-3p | Hspd1 | up |
| rno-miR-133a-5p | Asic4 | up |
| rno-miR-133a-3p | Abcb9 | up |
| rno-miR-133b-3p | Abcb9 | up |
| rno-miR-206-3p | Casp7 | up |
| rno-miR-206-3p | Kit | up |
| rno-miR-329-3p | Ctgf | up |
| rno-miR-133a-3p | Gcnt1 | up |
| rno-miR-133b-3p | Gcnt1 | up |
| rno-miR-329-3p | Gcnt1 | up |
| rno-miR-133a-3p | Lrat | up |
| rno-miR-133b-3p | Lrat | up |
| rno-miR-325-5p | Csnk1g1 | up |
| rno-miR-329-3p | Xylt1 | up |
| rno-miR-206-3p | Pi4ka | up |
| rno-miR-133a-3p | Mafg | up |
| rno-miR-133b-3p | Mafg | up |
| rno-miR-206-3p | Pafah1b2 | up |
| rno-miR-411-5p | Pafah1b2 | up |
| rno-miR-329-3p | Oat | up |
| rno-miR-675-3p | Casp2 | up |
| rno-miR-329-3p | Dap | up |
| rno-miR-411-5p | Itgad | up |
| rno-miR-133a-5p | Pdlim5 | up |
| rno-miR-325-5p | Araf | up |
| rno-miR-206-3p | Ppib | up |
| rno-miR-133a-3p | Zmat3 | up |
| rno-miR-133b-3p | Zmat3 | up |
| rno-miR-206-3p | Zmat3 | up |
| rno-miR-329-3p | Zmat3 | up |
| rno-miR-329-3p | Pex14 | up |
| rno-miR-206-3p | Cdc42 | up |
| rno-miR-133a-5p | Ddb1 | up |
| rno-miR-133a-5p | Rbm10 | up |
| rno-miR-329-3p | Bcl2l11 | up |
| rno-miR-133a-5p | Slc22a23 | up |
| rno-miR-329-3p | Prkab2 | up |
| rno-miR-133a-5p | Cul5 | up |
| rno-miR-133a-5p | Snap23 | up |
| rno-miR-329-3p | Sgta | up |
| rno-miR-675-3p | Olah | up |
| rno-miR-325-5p | Gabarapl2 | up |
| rno-miR-126a-3p | Srd5a2 | up |
| rno-miR-325-5p | Tfrc | up |
| rno-miR-329-3p | Tfrc | up |
| rno-miR-133a-3p | Csnk1g3 | up |
| rno-miR-133b-3p | Csnk1g3 | up |
| rno-miR-206-3p | Ireb2 | up |
| rno-miR-206-3p | Bche | up |
| rno-miR-133a-3p | Dlgap1 | up |
| rno-miR-133b-3p | Dlgap1 | up |
| rno-miR-329-3p | Fat2 | up |
| rno-miR-133a-5p | Cldn1 | up |
| rno-miR-411-5p | Aqp3 | up |
| rno-miR-133a-3p | Hrsp12 | up |
| rno-miR-133b-3p | Hrsp12 | up |
| rno-miR-329-3p | Htra1 | up |
| rno-miR-325-5p | Sult1c3 | up |
| rno-miR-133a-5p | Stk3 | up |
| rno-miR-206-3p | Stk3 | up |
| rno-miR-329-3p | Stk3 | up |
| rno-miR-329-3p | B4galt6 | up |
| rno-miR-329-3p | Kcnh1 | up |
| rno-miR-329-3p | Agap2 | up |
| rno-miR-329-3p | Prkaa1 | up |
| rno-miR-206-3p | Adamts4 | up |
| rno-miR-329-3p | Adamts4 | up |
| rno-miR-206-3p | Drp2 | up |
| rno-miR-325-5p | Drp2 | up |
| rno-miR-206-3p | Atp13a3 | up |
| rno-miR-329-3p | Phka2 | up |
| rno-miR-329-3p | Zc3h4 | up |
| rno-miR-133a-5p | Ndufa10 | up |
| rno-miR-329-3p | LOC678772 | up |
| rno-miR-329-3p | Rbpj | up |
| rno-miR-206-3p | Arhgap19 | up |
| rno-miR-329-3p | Mettl1 | up |
| rno-miR-329-3p | 9-Mar | up |
| rno-miR-133a-5p | Irf2bp2 | up |
| rno-miR-329-3p | Lypd6 | up |
| rno-miR-133a-5p | Ldlrad4 | up |
| rno-miR-133a-5p | XAF1 | up |
| rno-miR-329-3p | Tmem178b | up |
| rno-miR-675-3p | Tmem178b | up |
| rno-miR-133a-3p | Adam12 | up |
| rno-miR-133b-3p | Adam12 | up |
| rno-miR-675-3p | Tcf7l2 | up |
| rno-miR-206-3p | Gpkow | up |
| rno-miR-133a-3p | LOC679894 | up |
| rno-miR-133b-3p | LOC679894 | up |
| rno-miR-206-3p | LOC679894 | up |
| rno-miR-329-3p | RGD1564247 | up |
| rno-miR-329-3p | Trpm7 | up |
| rno-miR-206-3p | LOC679924 | up |
| rno-miR-133a-5p | Tceal6 | up |
| rno-miR-206-3p | LOC679989 | up |
| rno-miR-206-3p | Hist1h3a | up |
| rno-miR-329-3p | Msrb3 | up |
| rno-miR-325-5p | Rprm | up |
| rno-miR-329-3p | Rprm | up |
| rno-miR-329-3p | Tlx2 | up |
| rno-miR-133a-3p | Zfp322a | up |
| rno-miR-133b-3p | Zfp322a | up |
| rno-miR-675-3p | Zfp322a | up |
| rno-miR-411-5p | Pcdhb20 | up |
| rno-miR-329-3p | Nudt11 | up |
| rno-miR-206-3p | Ccdc83 | up |
| rno-miR-329-3p | Gas2l3 | up |
| rno-miR-206-3p | Rsu1 | up |
| rno-miR-133a-3p | Haus3 | up |
| rno-miR-133b-3p | Haus3 | up |
| rno-miR-329-3p | Cd3eap | up |
| rno-miR-133a-5p | Whsc1 | up |
| rno-miR-206-3p | Rc3h1 | up |
| rno-miR-329-3p | Rc3h1 | up |
| rno-miR-206-3p | Lrch2 | up |
| rno-miR-675-3p | LOC680606 | up |
| rno-miR-133a-5p | LOC680663 | up |
| rno-miR-133a-3p | Golm1 | up |
| rno-miR-133b-3p | Golm1 | up |
| rno-miR-133a-3p | Zcchc16 | up |
| rno-miR-133b-3p | Zcchc16 | up |
| rno-miR-133a-3p | Treml2 | up |
| rno-miR-133b-3p | Treml2 | up |
| rno-miR-126a-3p | Eppk1 | up |
| rno-miR-329-3p | Lrtm2 | up |
| rno-miR-133a-3p | LOC680885 | up |
| rno-miR-133b-3p | LOC680885 | up |
| rno-miR-329-3p | Efcab12 | up |
| rno-miR-325-5p | LOC680913 | up |
| rno-miR-329-3p | Paqr6 | up |
| rno-miR-206-3p | Qsox2 | up |
| rno-miR-206-3p | Acnat1 | up |
| rno-miR-411-5p | Acnat1 | up |
| rno-miR-133a-3p | Slbp | up |
| rno-miR-133b-3p | Slbp | up |
| rno-miR-133a-3p | Fcamr | up |
| rno-miR-133b-3p | Fcamr | up |
| rno-miR-206-3p | LOC681180 | up |
| rno-miR-133a-3p | Zfp862 | up |
| rno-miR-133b-3p | Zfp862 | up |
| rno-miR-206-3p | Lysmd4 | up |
| rno-miR-206-3p | Slc25a18 | up |
| rno-miR-329-3p | Ism2 | up |
| rno-miR-325-5p | Dcdc2b | up |
| rno-miR-329-3p | Taf12 | up |
| rno-miR-325-5p | Aida | up |
| rno-miR-133a-5p | Mia3 | up |
| rno-miR-329-3p | Tnfaip3 | up |
| rno-miR-133a-5p | LOC683420 | up |
| rno-miR-329-3p | LOC683422 | up |
| rno-miR-329-3p | Tead4 | up |
| rno-miR-133a-3p | Cstf2 | up |
| rno-miR-133b-3p | Cstf2 | up |
| rno-miR-133a-5p | Sik3 | up |
| rno-miR-133a-3p | LOC684122 | up |
| rno-miR-133b-3p | LOC684122 | up |
| rno-miR-206-3p | LOC684545 | up |
| rno-miR-133a-5p | Gpr52 | up |
| rno-miR-329-3p | Gpr52 | up |
| rno-miR-206-3p | LOC684871 | up |
| rno-miR-133a-3p | Zmat4 | up |
| rno-miR-133b-3p | Zmat4 | up |
| rno-miR-206-3p | LOC684998 | up |
| rno-miR-325-5p | LOC685025 | up |
| rno-miR-329-3p | LOC685069 | up |
| rno-miR-206-3p | LOC685081 | up |
| rno-miR-206-3p | Mea1 | up |
| rno-miR-133a-5p | Gpatch8 | up |
| rno-miR-325-5p | Ears2l1 | up |
| rno-miR-133a-3p | Eny2 | up |
| rno-miR-133b-3p | Eny2 | up |
| rno-miR-325-5p | L1td1 | up |
| rno-miR-206-3p | Ankrd13c | up |
| rno-miR-133a-5p | LOC685378 | up |
| rno-miR-329-3p | Ppp1r26 | up |
| rno-miR-133a-3p | Emid1 | up |
| rno-miR-133b-3p | Emid1 | up |
| rno-miR-133a-3p | Zfp385a | up |
| rno-miR-133b-3p | Zfp385a | up |
| rno-miR-206-3p | Clgn | up |
| rno-miR-329-3p | Gtf3c4 | up |
| rno-miR-675-3p | LOC685574 | up |
| rno-miR-411-5p | Dnal1 | up |
| rno-miR-325-5p | LOC685680 | up |
| rno-miR-133a-3p | Eid2 | up |
| rno-miR-133b-3p | Eid2 | up |
| rno-miR-126a-3p | Supt3h | up |
| rno-miR-329-3p | Supt3h | up |
| rno-miR-411-5p | Supt3h | up |
| rno-miR-133a-5p | Aldh8a1 | up |
| rno-miR-325-5p | Jazf1 | up |
| rno-miR-133a-5p | Srrm3 | up |
| rno-miR-329-3p | LOC685909 | up |
| rno-miR-411-5p | Uroc1 | up |
| rno-miR-206-3p | Meis1 | up |
| rno-miR-133a-5p | Wdr82 | up |
| rno-miR-206-3p | Tmprss11a | up |
| rno-miR-325-5p | Iqsec1 | up |
| rno-miR-329-3p | LOC686753 | up |
| rno-miR-329-3p | Mphosph6 | up |
| rno-miR-206-3p | Hdac9 | up |
| rno-miR-411-5p | Hdac9 | up |
| rno-miR-329-3p | LOC687022 | up |
| rno-miR-325-5p | Etnppl | up |
| rno-miR-325-5p | Ric3 | up |
| rno-miR-329-3p | Ric3 | up |
| rno-miR-329-3p | Brap | up |
| rno-miR-329-3p | LOC687508 | up |
| rno-miR-329-3p | Stambpl1 | up |
| rno-miR-325-5p | Pgap3 | up |
| rno-miR-329-3p | Pgap3 | up |
| rno-miR-206-3p | Rnf157 | up |
| rno-miR-133a-3p | Znf750 | up |
| rno-miR-133b-3p | Znf750 | up |
| rno-miR-329-3p | Znf750 | up |
| rno-miR-206-3p | Mab21l1 | up |
| rno-miR-329-3p | Prmt8 | up |
| rno-miR-133a-3p | Atg10 | up |
| rno-miR-133b-3p | Atg10 | up |
| rno-miR-325-5p | Elmod2 | up |
| rno-miR-329-3p | Elmod2 | up |
| rno-miR-206-3p | Rundc3b | up |
| rno-miR-325-5p | Rundc3b | up |
| rno-miR-329-3p | Rundc3b | up |
| rno-miR-675-3p | Tcf23 | up |
| rno-miR-206-3p | LOC688708 | up |
| rno-miR-329-3p | Slirp | up |
| rno-miR-133a-3p | Pet100 | up |
| rno-miR-133b-3p | Pet100 | up |
| rno-miR-411-5p | Tbx10 | up |
| rno-miR-206-3p | Nap1l5 | up |
| rno-miR-133a-3p | Mtx3 | up |
| rno-miR-133b-3p | Mtx3 | up |
| rno-miR-675-3p | Mtx3 | up |
| rno-miR-325-5p | LOC689065 | up |
| rno-miR-133a-3p | Parvg | up |
| rno-miR-133b-3p | Parvg | up |
| rno-miR-206-3p | Vstm2a | up |
| rno-miR-329-3p | Tmem25 | up |
| rno-miR-206-3p | Chmp1b | up |
| rno-miR-329-3p | Chmp1b | up |
| rno-miR-126a-3p | Tbc1d22a | up |
| rno-miR-329-3p | Tbc1d22a | up |
| rno-miR-206-3p | LOC689479 | up |
| rno-miR-329-3p | Ryr2 | up |
| rno-miR-329-3p | Nkain3 | up |
| rno-miR-133a-5p | Psmg4 | up |
| rno-miR-329-3p | Psmg4 | up |
| rno-miR-206-3p | LOC689629 | up |
| rno-miR-133a-3p | Tdrd12 | up |
| rno-miR-133b-3p | Tdrd12 | up |
| rno-miR-206-3p | Vsx1 | up |
| rno-miR-411-5p | Ccdc167 | up |
| rno-miR-325-5p | RGD1565309 | up |
| rno-miR-325-5p | Klrb1 | up |
| rno-miR-133a-3p | Klrg2 | up |
| rno-miR-133b-3p | Klrg2 | up |
| rno-miR-206-3p | Fam91a1 | up |
| rno-miR-206-3p | Xlr3a | up |
| rno-miR-133a-3p | LOC690085 | up |
| rno-miR-133b-3p | LOC690085 | up |
| rno-miR-206-3p | Dnhd1-ps1 | up |
| rno-miR-325-5p | Zfp939 | up |
| rno-miR-133a-3p | Fam168b | up |
| rno-miR-133b-3p | Fam168b | up |
| rno-miR-329-3p | Pdf | up |
| rno-miR-133a-3p | Polr3gl | up |
| rno-miR-133b-3p | Polr3gl | up |
| rno-miR-411-5p | Yaf2 | up |
| rno-miR-325-5p | LOC690286 | up |
| rno-miR-133a-3p | Derl3 | up |
| rno-miR-133b-3p | Derl3 | up |
| rno-miR-329-3p | Lyrm2 | up |
| rno-miR-325-5p | Vangl1 | up |
| rno-miR-206-3p | Rab36 | up |
| rno-miR-675-3p | Ttll4 | up |
| rno-miR-206-3p | Adam30 | up |
| rno-miR-133a-5p | Tcl1a | up |
| rno-miR-133a-5p | Mxra7 | up |
| rno-miR-329-3p | Fbxo3 | up |
| rno-miR-325-5p | Wdr59 | up |
| rno-miR-329-3p | Adat1 | up |
| rno-miR-133a-3p | Klf16 | up |
| rno-miR-133b-3p | Klf16 | up |
| rno-miR-206-3p | Klf16 | up |
| rno-miR-206-3p | LOC690826 | up |
| rno-miR-329-3p | Sh3bp5l | up |
| rno-miR-325-5p | Muc15 | up |
| rno-miR-133a-5p | Uevld | up |
| rno-miR-206-3p | Uevld | up |
| rno-miR-411-5p | Elof1 | up |
| rno-miR-206-3p | Slc12a6 | up |
| rno-miR-325-5p | Slc12a6 | up |
| rno-miR-206-3p | Gemin5 | up |
| rno-miR-133a-3p | 6-Sep | up |
| rno-miR-133b-3p | 6-Sep | up |
| rno-miR-206-3p | Stk38l | up |
| rno-miR-133a-5p | LOC691418 | up |
| rno-miR-206-3p | LOC691422 | up |
| rno-miR-206-3p | Shisa7 | up |
| rno-miR-133a-3p | Slc25a54 | up |
| rno-miR-133b-3p | Slc25a54 | up |
| rno-miR-133a-5p | LOC691519 | up |
| rno-miR-206-3p | LOC691519 | up |
| rno-miR-206-3p | Lilra5 | up |
| rno-miR-329-3p | Pacs2 | up |
| rno-miR-133a-3p | RGD1559979 | up |
| rno-miR-133b-3p | RGD1559979 | up |
| rno-miR-325-5p | LOC691693 | up |
| rno-miR-206-3p | LOC691712 | up |
| rno-miR-206-3p | Ankrd63 | up |
| rno-miR-206-3p | Kctd4 | up |
| rno-miR-133a-3p | LOC691920 | up |
| rno-miR-133b-3p | LOC691920 | up |
| rno-miR-329-3p | Fam124a | up |
| rno-miR-133a-5p | LOC691952 | up |
| rno-miR-329-3p | LOC691952 | up |
| rno-miR-133a-3p | Sqrdl | up |
| rno-miR-133b-3p | Sqrdl | up |
| rno-miR-206-3p | LOC691984 | up |
| rno-miR-133a-3p | Pdxp | up |
| rno-miR-133b-3p | Pdxp | up |
| rno-miR-206-3p | Scn9a | up |
| rno-miR-325-5p | Shank1 | up |
| rno-miR-675-3p | Per3 | up |
| rno-miR-206-3p | Apaf1 | up |
| rno-miR-206-3p | Csf1 | up |
| rno-miR-133a-5p | Srebf1 | up |
| rno-miR-133a-5p | Prkaa2 | up |
| rno-miR-206-3p | Duox2 | up |
| rno-miR-133a-3p | Pigm | up |
| rno-miR-133b-3p | Pigm | up |
| rno-miR-411-5p | Arf2 | up |
| rno-miR-206-3p | Dnajc5 | up |
| rno-miR-329-3p | Dnajc5 | up |
| rno-miR-206-3p | Gdf10 | up |
| rno-miR-133a-3p | Hsf1 | up |
| rno-miR-133b-3p | Hsf1 | up |
| rno-miR-133a-3p | Abca2 | up |
| rno-miR-133b-3p | Abca2 | up |
| rno-miR-133a-5p | Aco2 | up |
| rno-miR-325-5p | Aspa | up |
| rno-miR-329-3p | Adamts1 | up |
| rno-miR-206-3p | Zfp36 | up |
| rno-miR-206-3p | Luzp1 | up |
| rno-miR-206-3p | Zbtb10 | up |
| rno-miR-675-3p | Inpp4a | up |
| rno-miR-411-5p | Erap1 | up |
| rno-miR-206-3p | Syt13 | up |
| rno-miR-206-3p | Nptxr | up |
| rno-miR-325-5p | Nptxr | up |
| rno-miR-329-3p | Mef2d | up |
| rno-miR-329-3p | Nrp2 | up |
| rno-miR-329-3p | Adar | up |
| rno-miR-126a-3p | Creb1 | up |
| rno-miR-206-3p | Creb1 | up |
| rno-miR-133a-3p | Cspg4 | up |
| rno-miR-133b-3p | Cspg4 | up |
| rno-miR-411-5p | Ctbs | up |
| rno-miR-206-3p | Gna12 | up |
| rno-miR-329-3p | Itpr2 | up |
| rno-miR-329-3p | Lifr | up |
| rno-miR-411-5p | Lifr | up |
| rno-miR-133a-3p | Mipep | up |
| rno-miR-133b-3p | Mipep | up |
| rno-miR-206-3p | Mipep | up |
| rno-miR-325-5p | Mipep | up |
| rno-miR-329-3p | Mmp14 | up |
| rno-miR-325-5p | Aldh6a1 | up |
| rno-miR-133a-3p | Camlg | up |
| rno-miR-133b-3p | Camlg | up |
| rno-miR-329-3p | Neo1 | up |
| rno-miR-133a-3p | Pde1c | up |
| rno-miR-133b-3p | Pde1c | up |
| rno-miR-325-5p | Pde1c | up |
| rno-miR-675-3p | Pde2a | up |
| rno-miR-329-3p | Pde7a | up |
| rno-miR-133a-5p | Pdpk1 | up |
| rno-miR-329-3p | Pdpk1 | up |
| rno-miR-329-3p | Pggt1b | up |
| rno-miR-206-3p | Pls3 | up |
| rno-miR-411-5p | Soat1 | up |
| rno-miR-329-3p | Stx3 | up |
| rno-miR-411-5p | Stx4 | up |
| rno-miR-206-3p | Tmsb4x | up |
| rno-miR-329-3p | Usf2 | up |
| rno-miR-325-5p | Doc2b | up |
| rno-miR-675-3p | Doc2b | up |
| rno-miR-206-3p | Dgkz | up |
| rno-miR-206-3p | Pard3 | up |
| rno-miR-133a-5p | Celsr2 | up |
| rno-miR-675-3p | Prep | up |
| rno-miR-411-5p | Ugdh | up |
| rno-miR-329-3p | Cdh1 | up |
| rno-miR-133a-3p | Timeless | up |
| rno-miR-133b-3p | Timeless | up |
| rno-miR-329-3p | Acox3 | up |
| rno-miR-325-5p | Atrn | up |
| rno-miR-675-3p | Dbnl | up |
| rno-miR-206-3p | Mkln1 | up |
| rno-miR-329-3p | Abcb11 | up |
| rno-miR-206-3p | Pafah1b1 | up |
| rno-miR-206-3p | Sort1 | up |
| rno-miR-206-3p | Thbd | up |
| rno-miR-325-5p | Thbd | up |
| rno-miR-329-3p | Apba1 | up |
| rno-miR-133a-3p | Apba3 | up |
| rno-miR-133b-3p | Apba3 | up |
| rno-miR-329-3p | Hipk3 | up |
| rno-miR-206-3p | Ugcg | up |
| rno-miR-206-3p | Gabbr2 | up |
| rno-miR-206-3p | Cngb1 | up |
| rno-miR-329-3p | Rtn4 | up |
| rno-miR-206-3p | Trpv1 | up |
| rno-miR-133a-3p | Gipc1 | up |
| rno-miR-133b-3p | Gipc1 | up |
| rno-miR-133a-5p | Cxxc4 | up |
| rno-miR-133a-5p | Dclk1 | up |
| rno-miR-325-5p | Dclk1 | up |
| rno-miR-133a-3p | C3ar1 | up |
| rno-miR-133b-3p | C3ar1 | up |
| rno-miR-411-5p | Ralbp1 | up |
| rno-miR-329-3p | Gsk3b | up |
| rno-miR-206-3p | Clcn3 | up |
| rno-miR-325-5p | Rab6a | up |
| rno-miR-325-5p | Dhh | up |
| rno-miR-329-3p | Dcx | up |
| rno-miR-329-3p | Puf60 | up |
| rno-miR-206-3p | Sfrp1 | up |
| rno-miR-325-5p | Klf5 | up |
| rno-miR-133a-3p | Asah1 | up |
| rno-miR-133b-3p | Asah1 | up |
| rno-miR-411-5p | Maged1 | up |
| rno-miR-206-3p | Strbp | up |
| rno-miR-329-3p | Gab2 | up |
| rno-miR-329-3p | Slc17a6 | up |
| rno-miR-329-3p | Ran | up |
| rno-miR-133a-5p | Otof | up |
| rno-miR-133a-3p | Srm | up |
| rno-miR-133b-3p | Srm | up |
| rno-miR-206-3p | Pola1 | up |
| rno-miR-325-5p | Pik3cb | up |
| rno-miR-133a-5p | Gas7 | up |
| rno-miR-206-3p | Gas7 | up |
| rno-miR-325-5p | Gas7 | up |
| rno-miR-329-3p | Kif3c | up |
| rno-miR-329-3p | Pex11a | up |
| rno-miR-206-3p | Slc44a1 | up |
| rno-miR-329-3p | Slc44a1 | up |
| rno-miR-206-3p | Cnga3 | up |
| rno-miR-206-3p | Ajuba | up |
| rno-miR-206-3p | Bmp7 | up |
| rno-miR-133a-3p | Nol3 | up |
| rno-miR-133b-3p | Nol3 | up |
| rno-miR-325-5p | Elf1 | up |
| rno-miR-329-3p | Elf1 | up |
| rno-miR-325-5p | Slc5a7 | up |
| rno-miR-411-5p | Rhoq | up |
| rno-miR-206-3p | Smad9 | up |
| rno-miR-206-3p | Ciita | up |
| rno-miR-206-3p | Idi1 | up |
| rno-miR-206-3p | Rfc1 | up |
| rno-miR-329-3p | Vegfb | up |
| rno-miR-133a-3p | Pip4k2b | up |
| rno-miR-133b-3p | Pip4k2b | up |
| rno-miR-329-3p | Sh2b1 | up |
| rno-miR-133a-5p | Rnf138 | up |
| rno-miR-206-3p | Ece1 | up |
| rno-miR-329-3p | Ece1 | up |
| rno-miR-329-3p | Nudt4 | up |
| rno-miR-329-3p | Fez2 | up |
| rno-miR-206-3p | Clic5 | up |
| rno-miR-675-3p | Kcnj13 | up |

**Table S4 GO analyses of target genes**

| GO Name | Diff Gene Counts in GO | Gene Amount in GO | Enrichment Score | p-value |
| --- | --- | --- | --- | --- |
| biological_process | 192 | 1408 | 2.572189 | 3.24E-33 |
| detection of chemical stimulus involved in sensory perception of smell | 1 | 1268 | 0.014876 | 9.92E-29 |
| positive regulation of transcription, DNA-dependent | 95 | 495 | 3.620118 | 1.23E-27 |
| regulation of transcription, DNA-dependent | 109 | 681 | 3.019143 | 1.10E-24 |
| positive regulation of transcription from RNA polymerase II promoter | 111 | 715 | 2.928338 | 5.22E-24 |
| negative regulation of transcription from RNA polymerase II promoter | 84 | 499 | 3.175288 | 1.12E-20 |
| positive regulation of cell proliferation | 72 | 388 | 3.500299 | 2.51E-20 |
| negative regulation of apoptotic process | 81 | 478 | 3.196402 | 3.69E-20 |
| negative regulation of transcription, DNA-dependent | 73 | 420 | 3.27852 | 6.51E-19 |
| protein phosphorylation | 65 | 353 | 3.473305 | 2.79E-18 |
| transcription, DNA-dependent | 90 | 640 | 2.65257 | 7.71E-17 |
| signal transduction | 57 | 377 | 2.851923 | 2.26E-12 |
| heart development | 38 | 191 | 3.752793 | 3.07E-12 |
| Wnt receptor signaling pathway | 29 | 116 | 4.71568 | 3.45E-12 |
| G-protein coupled receptor signaling pathway | 25 | 1454 | 0.324325 | 3.76E-12 |
| intracellular signal transduction | 42 | 236 | 3.356925 | 1.02E-11 |
| in utero embryonic development | 43 | 247 | 3.283793 | 1.22E-11 |
| cell adhesion | 44 | 260 | 3.192153 | 1.86E-11 |
| homophilic cell adhesion | 28 | 122 | 4.329149 | 7.54E-11 |
| response to hypoxia | 41 | 242 | 3.19575 | 9.02E-11 |
| cell migration | 28 | 126 | 4.191716 | 1.70E-10 |
| positive regulation of cell migration | 28 | 131 | 4.031726 | 4.46E-10 |
| protein transport | 42 | 271 | 2.923374 | 9.29E-10 |
| intracellular protein kinase cascade | 21 | 79 | 5.014141 | 1.14E-09 |
| angiogenesis | 29 | 152 | 3.598808 | 3.60E-09 |
| vesicle-mediated transport | 25 | 120 | 3.929733 | 7.08E-09 |
| negative regulation of cell proliferation | 43 | 308 | 2.633432 | 1.51E-08 |
| regulation of transcription from RNA polymerase II promoter | 33 | 202 | 3.081534 | 1.73E-08 |
| apoptotic process | 47 | 358 | 2.476391 | 2.89E-08 |
| brain development | 36 | 239 | 2.841247 | 3.37E-08 |
| G2/M transition of mitotic cell cycle | 13 | 36 | 6.811538 | 3.70E-08 |
| lamellipodium assembly | 11 | 25 | 8.299597 | 4.06E-08 |
| response to drug | 55 | 462 | 2.245562 | 5.17E-08 |
| positive regulation of protein kinase B signaling cascade | 17 | 65 | 4.933327 | 6.37E-08 |
| positive regulation of apoptotic process | 37 | 258 | 2.705119 | 8.04E-08 |
| small GTPase mediated signal transduction | 34 | 227 | 2.825253 | 9.57E-08 |
| activation of MAPK activity | 17 | 68 | 4.71568 | 1.32E-07 |
| protein autophosphorylation | 27 | 158 | 3.223376 | 1.45E-07 |
| regulation of cell migration | 14 | 47 | 5.618683 | 1.74E-07 |
| protein ubiquitination | 30 | 192 | 2.9473 | 2.23E-07 |
| microtubule cytoskeleton organization | 16 | 63 | 4.790532 | 2.52E-07 |
| canonical Wnt receptor signaling pathway | 18 | 79 | 4.297835 | 2.54E-07 |
| neuron differentiation | 23 | 125 | 3.470741 | 3.31E-07 |
| actin cytoskeleton organization | 22 | 116 | 3.577412 | 3.51E-07 |
| positive regulation of GTPase activity | 13 | 44 | 5.573076 | 5.58E-07 |
| neuron projection morphogenesis | 14 | 52 | 5.078425 | 7.01E-07 |
| negative regulation of gene expression | 19 | 93 | 3.853674 | 7.14E-07 |
| induction of apoptosis | 27 | 172 | 2.961008 | 8.55E-07 |
| positive regulation of gene expression | 28 | 185 | 2.854898 | 1.13E-06 |
| protein stabilization | 15 | 62 | 4.563561 | 1.23E-06 |
| proteolysis | 53 | 483 | 2.069822 | 1.25E-06 |
| endocytosis | 18 | 88 | 3.858284 | 1.41E-06 |
| negative regulation of neuron apoptotic process | 21 | 116 | 3.414803 | 1.48E-06 |
| proteasomal ubiquitin-dependent protein catabolic process | 15 | 63 | 4.491124 | 1.53E-06 |
| cellular response to interleukin-1 | 13 | 48 | 5.108653 | 1.70E-06 |
| protein complex assembly | 14 | 56 | 4.71568 | 1.88E-06 |
| chloride transport | 13 | 49 | 5.004395 | 2.20E-06 |
| lung development | 21 | 119 | 3.328715 | 2.29E-06 |
| neuron projection development | 19 | 100 | 3.583917 | 2.30E-06 |
| phosphatidylinositol-mediated signaling | 10 | 29 | 6.504386 | 2.72E-06 |
| autophagy | 13 | 50 | 4.904307 | 2.83E-06 |
| mitosis | 20 | 111 | 3.398688 | 2.92E-06 |
| positive regulation of peptidyl-serine phosphorylation | 13 | 51 | 4.808144 | 3.61E-06 |
| cell proliferation | 27 | 185 | 2.752938 | 3.68E-06 |
| regulation of ARF GTPase activity | 9 | 24 | 7.07352 | 4.12E-06 |
| embryo development | 21 | 125 | 3.168937 | 5.23E-06 |
| insulin receptor signaling pathway | 12 | 45 | 5.030059 | 5.36E-06 |
| negative regulation of insulin secretion | 10 | 31 | 6.084749 | 5.46E-06 |
| intracellular protein transport | 25 | 167 | 2.823761 | 5.47E-06 |
| memory | 14 | 61 | 4.329149 | 5.69E-06 |
| hippo signaling cascade | 7 | 14 | 9.43136 | 5.76E-06 |
| axonogenesis | 17 | 90 | 3.562958 | 9.02E-06 |
| positive regulation of MAPK cascade | 16 | 81 | 3.725969 | 9.33E-06 |
| dopamine receptor signaling pathway | 7 | 15 | 8.802603 | 1.03E-05 |
| cell division | 23 | 152 | 2.854227 | 1.12E-05 |
| transmembrane transport | 21 | 133 | 2.978324 | 1.43E-05 |
| cellular response to hypoxia | 14 | 66 | 4.001183 | 1.52E-05 |
| forebrain development | 16 | 84 | 3.592899 | 1.53E-05 |
| aging | 26 | 189 | 2.594872 | 1.70E-05 |
| cell differentiation | 33 | 271 | 2.296936 | 1.74E-05 |
| G1/S transition of mitotic cell cycle | 12 | 50 | 4.527053 | 1.77E-05 |
| cAMP-mediated signaling | 8 | 22 | 6.859171 | 2.02E-05 |
| positive regulation of DNA replication | 11 | 43 | 4.825347 | 2.16E-05 |
| neuron development | 11 | 43 | 4.825347 | 2.16E-05 |
| cell surface receptor signaling pathway | 14 | 68 | 3.883501 | 2.20E-05 |
| hemopoiesis | 14 | 69 | 3.827219 | 2.62E-05 |
| post-embryonic development | 16 | 88 | 3.429586 | 2.83E-05 |
| positive regulation of Rac GTPase activity | 7 | 17 | 7.767003 | 2.83E-05 |
| phosphatidylinositol phosphorylation | 7 | 17 | 7.767003 | 2.83E-05 |
| phosphatidylinositol-3-phosphate biosynthetic process | 6 | 12 | 9.43136 | 3.09E-05 |
| negative regulation of vascular permeability | 6 | 12 | 9.43136 | 3.09E-05 |
| translation | 4 | 384 | 0.196487 | 3.32E-05 |
| fat cell differentiation | 12 | 53 | 4.270805 | 3.35E-05 |
| ureteric bud development | 11 | 45 | 4.610887 | 3.46E-05 |
| cellular protein localization | 11 | 45 | 4.610887 | 3.46E-05 |
| protein ubiquitination involved in ubiquitin-dependent protein catabolic process | 11 | 45 | 4.610887 | 3.46E-05 |
| exocytosis | 13 | 62 | 3.955087 | 3.65E-05 |
| transport | 17 | 100 | 3.206662 | 3.88E-05 |
| regulation of gene expression | 19 | 123 | 2.913754 | 5.27E-05 |
| sodium ion transmembrane transport | 10 | 39 | 4.836595 | 5.31E-05 |
| stem cell differentiation | 8 | 25 | 6.036071 | 5.92E-05 |
| protein homooligomerization | 23 | 168 | 2.582396 | 5.92E-05 |
| activation of cysteine-type endopeptidase activity involved in apoptotic process | 14 | 74 | 3.568623 | 6.03E-05 |
| protein dephosphorylation | 15 | 84 | 3.368343 | 6.49E-05 |
| positive regulation of protein kinase activity | 11 | 48 | 4.322707 | 6.64E-05 |
| phosphatidylinositol metabolic process | 7 | 19 | 6.949423 | 6.68E-05 |
| regulation of synaptic plasticity | 10 | 40 | 4.71568 | 6.74E-05 |
| protein localization | 13 | 66 | 3.715384 | 7.38E-05 |
| response to cytokine stimulus | 16 | 95 | 3.176879 | 7.60E-05 |
| adenylate cyclase-modulating G-protein coupled receptor signaling pathway | 9 | 33 | 5.144378 | 7.87E-05 |
| synapse assembly | 9 | 33 | 5.144378 | 7.87E-05 |
| activation of JUN kinase activity | 8 | 26 | 5.803914 | 8.15E-05 |
| elevation of cytosolic calcium ion concentration | 19 | 127 | 2.821982 | 8.31E-05 |
| cytoplasmic mRNA processing body assembly | 5 | 9 | 10.47929 | 8.78E-05 |
| positive regulation of glycolysis | 5 | 9 | 10.47929 | 8.78E-05 |
| regulation of organ growth | 5 | 9 | 10.47929 | 8.78E-05 |
| behavioral response to ethanol | 5 | 9 | 10.47929 | 8.78E-05 |
| ubiquitin-dependent protein catabolic process | 20 | 139 | 2.71406 | 9.35E-05 |
| response to glucose stimulus | 16 | 97 | 3.111377 | 9.89E-05 |
| transcription from RNA polymerase II promoter | 18 | 118 | 2.877364 | 0.000101 |
| positive regulation of fat cell differentiation | 8 | 27 | 5.588954 | 0.00011 |
| response to stress | 20 | 141 | 2.675563 | 0.000115 |
| negative regulation of canonical Wnt receptor signaling pathway | 14 | 79 | 3.342761 | 0.000128 |
| iron ion homeostasis | 7 | 21 | 6.287573 | 0.00014 |
| cellular response to glucose starvation | 6 | 15 | 7.545088 | 0.000146 |
| response to morphine | 8 | 28 | 5.389349 | 0.000147 |
| dendrite morphogenesis | 8 | 28 | 5.389349 | 0.000147 |
| osteoblast differentiation | 12 | 61 | 3.710699 | 0.000148 |
| spermatogenesis | 32 | 283 | 2.132887 | 0.000154 |
| calcium ion transmembrane transport | 12 | 62 | 3.650849 | 0.000174 |
| mRNA processing | 18 | 124 | 2.738137 | 0.000195 |
| negative regulation of protein catabolic process | 7 | 22 | 6.001775 | 0.000196 |
| negative regulation of NF-kappaB transcription factor activity | 11 | 54 | 3.842406 | 0.00021 |
| Golgi to endosome transport | 4 | 6 | 12.57515 | 0.000217 |
| negative regulation of interleukin-8 production | 4 | 6 | 12.57515 | 0.000217 |
| response to oxidative stress | 18 | 125 | 2.716232 | 0.000217 |
| SCF-dependent proteasomal ubiquitin-dependent protein catabolic process | 6 | 16 | 7.07352 | 0.000223 |
| response to heat | 12 | 64 | 3.53676 | 0.00024 |
| cell fate commitment | 12 | 64 | 3.53676 | 0.00024 |
| regulation of apoptotic process | 18 | 126 | 2.694674 | 0.000241 |
| negative regulation of Wnt receptor signaling pathway | 10 | 46 | 4.100591 | 0.000242 |
| sodium ion transport | 13 | 74 | 3.313721 | 0.000254 |
| ion transmembrane transport | 15 | 95 | 2.978324 | 0.00028 |
| lymph vessel development | 5 | 11 | 8.573964 | 0.000294 |
| positive regulation of cardiac muscle hypertrophy | 5 | 11 | 8.573964 | 0.000294 |
| negative regulation of protein complex assembly | 5 | 11 | 8.573964 | 0.000294 |
| reduction of cytosolic calcium ion concentration | 5 | 11 | 8.573964 | 0.000294 |
| lipid phosphorylation | 3 | 3 | 18.86272 | 0.000298 |
| regulation of cell proliferation | 19 | 140 | 2.559941 | 0.000316 |
| neuron apoptotic process | 8 | 31 | 4.867799 | 0.000324 |
| ovulation cycle | 6 | 17 | 6.657431 | 0.000329 |
| brain morphogenesis | 6 | 17 | 6.657431 | 0.000329 |
| elevation of cytosolic calcium ion concentration involved in phospholipase C-activating G-protein coupled signaling pathway | 6 | 17 | 6.657431 | 0.000329 |
| blood vessel development | 11 | 57 | 3.640174 | 0.00035 |
| learning or memory | 10 | 48 | 3.929733 | 0.000353 |
| circadian rhythm | 12 | 67 | 3.378398 | 0.000379 |
| sensory perception of pain | 12 | 67 | 3.378398 | 0.000379 |
| response to DNA damage stimulus | 20 | 154 | 2.449704 | 0.000394 |
| response to estrogen stimulus | 15 | 98 | 2.887151 | 0.000399 |
| transforming growth factor beta receptor signaling pathway | 11 | 58 | 3.577412 | 0.000411 |
| DNA repair | 19 | 143 | 2.506236 | 0.000418 |
| cell communication | 12 | 68 | 3.328715 | 0.000438 |
| protein O-linked glycosylation | 6 | 18 | 6.287573 | 0.000471 |
| negative regulation of mitotic cell cycle | 6 | 18 | 6.287573 | 0.000471 |
| calcium ion transport | 14 | 89 | 2.96717 | 0.00048 |
| gastrulation with mouth forming second | 7 | 25 | 5.281562 | 0.000481 |
| positive regulation of Wnt receptor signaling pathway | 7 | 25 | 5.281562 | 0.000481 |
| actin filament bundle assembly | 7 | 25 | 5.281562 | 0.000481 |
| synaptic transmission, cholinergic | 5 | 12 | 7.859467 | 0.000482 |
| negative chemotaxis | 5 | 12 | 7.859467 | 0.000482 |
| positive regulation of excitatory postsynaptic membrane potential | 5 | 12 | 7.859467 | 0.000482 |
| ameboidal cell migration | 4 | 7 | 10.7787 | 0.000485 |
| desensitization of G-protein coupled receptor protein signaling pathway | 4 | 7 | 10.7787 | 0.000485 |
| mRNA splice site selection | 4 | 7 | 10.7787 | 0.000485 |
| negative regulation of smooth muscle cell apoptotic process | 4 | 7 | 10.7787 | 0.000485 |
| centrosome localization | 4 | 7 | 10.7787 | 0.000485 |
| cell cycle arrest | 13 | 79 | 3.103992 | 0.000502 |
| cilium assembly | 12 | 69 | 3.280473 | 0.000505 |
| amino acid transmembrane transport | 8 | 33 | 4.572781 | 0.000519 |
| positive regulation of JUN kinase activity | 8 | 33 | 4.572781 | 0.000519 |
| neuron migration | 15 | 101 | 2.801394 | 0.00056 |
| protein polyubiquitination | 11 | 60 | 3.458165 | 0.000561 |
| ion transport | 9 | 42 | 4.042012 | 0.000591 |
| keratinocyte differentiation | 9 | 42 | 4.042012 | 0.000591 |
| positive regulation of insulin secretion | 9 | 42 | 4.042012 | 0.000591 |
| microtubule-based movement | 11 | 61 | 3.401474 | 0.000652 |
| nervous system development | 21 | 167 | 2.371959 | 0.000656 |
| negative regulation of ossification | 6 | 19 | 5.956649 | 0.000658 |
| negative regulation of JNK cascade | 6 | 19 | 5.956649 | 0.000658 |
| positive regulation of neuron apoptotic process | 10 | 52 | 3.627446 | 0.000704 |
| multicellular organismal development | 23 | 197 | 2.202247 | 0.000717 |
| cAMP catabolic process | 5 | 13 | 7.254892 | 0.00075 |
| liver development | 16 | 115 | 2.624378 | 0.000761 |
| response to hormone stimulus | 14 | 93 | 2.839549 | 0.000766 |
| response to interleukin-1 | 8 | 35 | 4.311479 | 0.000801 |
| positive regulation of transforming growth factor beta receptor signaling pathway | 6 | 20 | 5.658816 | 0.000897 |
| positive regulation of synaptic transmission, glutamatergic | 6 | 20 | 5.658816 | 0.000897 |
| female meiosis | 4 | 8 | 9.43136 | 0.000928 |
| cerebral cortex development | 10 | 54 | 3.493096 | 0.000967 |
| cellular response to tumor necrosis factor | 10 | 54 | 3.493096 | 0.000967 |
| JNK cascade | 8 | 36 | 4.191716 | 0.000983 |
| termination of G-protein coupled receptor signaling pathway | 8 | 36 | 4.191716 | 0.000983 |
| branching morphogenesis of an epithelial tube | 8 | 36 | 4.191716 | 0.000983 |
| odontogenesis of dentin-containing tooth | 11 | 64 | 3.24203 | 0.001003 |
| response to lipopolysaccharide | 24 | 211 | 2.145523 | 0.001003 |
| negative regulation of protein phosphorylation | 9 | 45 | 3.772544 | 0.001018 |
| myelination | 9 | 45 | 3.772544 | 0.001018 |
| phospholipase C-activating G-protein coupled receptor signaling pathway | 7 | 28 | 4.71568 | 0.001031 |
| associative learning | 7 | 28 | 4.71568 | 0.001031 |
| regulation of cell shape | 13 | 85 | 2.884887 | 0.001046 |
| insulin-like growth factor receptor signaling pathway | 5 | 14 | 6.736686 | 0.001115 |
| positive regulation of neuron differentiation | 12 | 75 | 3.018035 | 0.001116 |
| leukemia inhibitory factor signaling pathway | 3 | 4 | 14.14704 | 0.001143 |
| smooth muscle contraction involved in micturition | 3 | 4 | 14.14704 | 0.001143 |
| negative regulation of cysteine-type endopeptidase activity involved in apoptotic process | 11 | 65 | 3.192153 | 0.00115 |
| adenylate cyclase-inhibiting G-protein coupled receptor signaling pathway | 6 | 21 | 5.389349 | 0.0012 |
| histone methylation | 6 | 21 | 5.389349 | 0.0012 |
| response to calcium ion | 12 | 76 | 2.978324 | 0.001262 |
| cation transport | 9 | 47 | 3.61201 | 0.001422 |
| dephosphorylation | 9 | 47 | 3.61201 | 0.001422 |
| cellular response to amino acid stimulus | 8 | 38 | 3.971099 | 0.001445 |
| cellular response to transforming growth factor beta stimulus | 8 | 38 | 3.971099 | 0.001445 |
| membrane protein ectodomain proteolysis | 6 | 22 | 5.144378 | 0.001577 |
| epidermal growth factor receptor signaling pathway | 6 | 22 | 5.144378 | 0.001577 |
| regulation of exocytosis | 6 | 22 | 5.144378 | 0.001577 |
| regulation of cell-matrix adhesion | 4 | 9 | 8.383431 | 0.001601 |
| germ cell migration | 4 | 9 | 8.383431 | 0.001601 |
| positive regulation of intracellular estrogen receptor signaling pathway | 4 | 9 | 8.383431 | 0.001601 |
| carbohydrate phosphorylation | 4 | 9 | 8.383431 | 0.001601 |
| positive regulation of peptidyl-tyrosine phosphorylation | 12 | 78 | 2.901957 | 0.001603 |
| peptidyl-threonine phosphorylation | 7 | 30 | 4.401301 | 0.001615 |
| positive regulation of mitosis | 7 | 30 | 4.401301 | 0.001615 |
| positive regulation of glucose import | 7 | 30 | 4.401301 | 0.001615 |
| positive regulation of ERK1 and ERK2 cascade | 13 | 89 | 2.755229 | 0.001636 |
| regulation of cell growth | 9 | 48 | 3.53676 | 0.001669 |
| integrin-mediated signaling pathway | 11 | 68 | 3.051322 | 0.001703 |
| axon guidance | 15 | 112 | 2.526257 | 0.001713 |
| negative regulation of transforming growth factor beta receptor signaling pathway | 8 | 39 | 3.869276 | 0.001734 |
| Notch signaling pathway | 10 | 58 | 3.252193 | 0.001739 |
| peptidyl-tyrosine phosphorylation | 10 | 58 | 3.252193 | 0.001739 |
| cell-cell adhesion | 13 | 90 | 2.724615 | 0.001821 |
| response to retinoic acid | 11 | 69 | 3.0071 | 0.00193 |
| skeletal muscle cell differentiation | 9 | 49 | 3.464581 | 0.00195 |
| glycogen metabolic process | 7 | 31 | 4.259324 | 0.001991 |
| regulation of excitatory postsynaptic membrane potential | 7 | 31 | 4.259324 | 0.001991 |
| autophagic vacuole assembly | 6 | 23 | 4.92071 | 0.002038 |
| ER to Golgi vesicle-mediated transport | 8 | 40 | 3.772544 | 0.002068 |
| spermatid development | 11 | 70 | 2.964142 | 0.002182 |
| positive regulation of cell-matrix adhesion | 5 | 16 | 5.8946 | 0.002226 |
| cellular membrane fusion | 5 | 16 | 5.8946 | 0.002226 |
| regulation of cell morphogenesis | 5 | 16 | 5.8946 | 0.002226 |
| circadian regulation of gene expression | 5 | 16 | 5.8946 | 0.002226 |
| axon extension | 5 | 16 | 5.8946 | 0.002226 |
| regulation of heart rate by cardiac conduction | 5 | 16 | 5.8946 | 0.002226 |
| protein kinase C-activating G-protein coupled receptor signaling pathway | 7 | 32 | 4.12622 | 0.002433 |
| multicellular organism growth | 11 | 71 | 2.922393 | 0.00246 |
| cell cycle | 16 | 128 | 2.35784 | 0.002488 |
| regulation of autophagy | 4 | 10 | 7.545088 | 0.002556 |
| L-glutamate transport | 4 | 10 | 7.545088 | 0.002556 |
| cerebral cortex radially oriented cell migration | 4 | 10 | 7.545088 | 0.002556 |
| central nervous system projection neuron axonogenesis | 4 | 10 | 7.545088 | 0.002556 |
| chondroitin sulfate biosynthetic process | 4 | 10 | 7.545088 | 0.002556 |
| regulation of vasodilation | 4 | 10 | 7.545088 | 0.002556 |
| positive regulation of vascular permeability | 4 | 10 | 7.545088 | 0.002556 |
| positive regulation of insulin-like growth factor receptor signaling pathway | 4 | 10 | 7.545088 | 0.002556 |
| somatic stem cell division | 4 | 10 | 7.545088 | 0.002556 |
| positive regulation of stress fiber assembly | 6 | 24 | 4.71568 | 0.002597 |
| cell chemotaxis | 6 | 24 | 4.71568 | 0.002597 |
| endocardium development | 3 | 5 | 11.31763 | 0.002745 |
| fructose 2,6-bisphosphate metabolic process | 3 | 5 | 11.31763 | 0.002745 |
| histone mRNA 3'-end processing | 3 | 5 | 11.31763 | 0.002745 |
| JUN phosphorylation | 3 | 5 | 11.31763 | 0.002745 |
| positive regulation of mammary gland epithelial cell proliferation | 3 | 5 | 11.31763 | 0.002745 |
| cellular triglyceride homeostasis | 3 | 5 | 11.31763 | 0.002745 |
| clustering of voltage-gated sodium channels | 3 | 5 | 11.31763 | 0.002745 |
| paraxial mesoderm formation | 3 | 5 | 11.31763 | 0.002745 |
| embryonic hindgut morphogenesis | 3 | 5 | 11.31763 | 0.002745 |
| regulation of phagocytosis | 3 | 5 | 11.31763 | 0.002745 |
| positive regulation of sarcomere organization | 3 | 5 | 11.31763 | 0.002745 |
| salivary gland cavitation | 3 | 5 | 11.31763 | 0.002745 |
| ciliary neurotrophic factor-mediated signaling pathway | 3 | 5 | 11.31763 | 0.002745 |
| organ morphogenesis | 11 | 72 | 2.881805 | 0.002767 |
| peptidyl-serine phosphorylation | 11 | 72 | 2.881805 | 0.002767 |
| negative regulation of cell growth | 14 | 106 | 2.491303 | 0.002878 |
| positive regulation of vasoconstriction | 7 | 33 | 4.001183 | 0.002948 |
| positive regulation of smooth muscle cell proliferation | 10 | 62 | 3.042374 | 0.002958 |
| mRNA polyadenylation | 5 | 17 | 5.547859 | 0.003017 |
| positive regulation of JAK-STAT cascade | 5 | 17 | 5.547859 | 0.003017 |
| cellular response to insulin stimulus | 12 | 84 | 2.694674 | 0.003114 |
| response to organic cyclic compound | 24 | 230 | 1.968284 | 0.003229 |
| negative regulation of protein binding | 6 | 25 | 4.527053 | 0.003265 |
| platelet-derived growth factor receptor signaling pathway | 6 | 25 | 4.527053 | 0.003265 |
| response to toxic substance | 13 | 96 | 2.554327 | 0.003334 |
| blood coagulation | 10 | 63 | 2.994083 | 0.003351 |
| positive regulation of epithelial cell proliferation | 10 | 63 | 2.994083 | 0.003351 |
| cellular calcium ion homeostasis | 11 | 74 | 2.803918 | 0.003475 |
| response to inorganic substance | 7 | 34 | 3.883501 | 0.003544 |
| regulation of cell adhesion | 7 | 34 | 3.883501 | 0.003544 |
| cellular response to lipopolysaccharide | 13 | 97 | 2.527993 | 0.003667 |
| response to ethanol | 17 | 142 | 2.258213 | 0.003747 |
| response to manganese ion | 4 | 11 | 6.859171 | 0.003849 |
| positive regulation of triglyceride biosynthetic process | 4 | 11 | 6.859171 | 0.003849 |
| Rac protein signal transduction | 4 | 11 | 6.859171 | 0.003849 |
| ventricular system development | 4 | 11 | 6.859171 | 0.003849 |
| histone lysine methylation | 4 | 11 | 6.859171 | 0.003849 |
| positive regulation of renal sodium excretion | 4 | 11 | 6.859171 | 0.003849 |
| megakaryocyte development | 4 | 11 | 6.859171 | 0.003849 |
| regulation of GTPase activity | 4 | 11 | 6.859171 | 0.003849 |
| negative regulation of translational initiation | 4 | 11 | 6.859171 | 0.003849 |
| embryonic viscerocranium morphogenesis | 4 | 11 | 6.859171 | 0.003849 |
| cellular response to electrical stimulus | 4 | 11 | 6.859171 | 0.003849 |
| response to estradiol stimulus | 17 | 143 | 2.242421 | 0.003932 |
| learning | 8 | 44 | 3.429586 | 0.003949 |
| visual learning | 8 | 44 | 3.429586 | 0.003949 |
| response to nicotine | 8 | 44 | 3.429586 | 0.003949 |
| cellular response to glucose stimulus | 8 | 44 | 3.429586 | 0.003949 |
| ovarian follicle development | 9 | 54 | 3.143787 | 0.003979 |
| embryonic skeletal system morphogenesis | 9 | 54 | 3.143787 | 0.003979 |
| humoral immune response | 5 | 18 | 5.239645 | 0.003996 |
| L-amino acid transport | 5 | 18 | 5.239645 | 0.003996 |
| filopodium assembly | 5 | 18 | 5.239645 | 0.003996 |
| male gonad development | 13 | 98 | 2.502198 | 0.004026 |
| cellular protein modification process | 6 | 26 | 4.352935 | 0.004055 |
| gastrulation | 6 | 26 | 4.352935 | 0.004055 |

**Table S5 Pathway analyses of target genes**

| Pathway Name | Diff Gene Counts in Pathway | Gene Amount in Pathway | Enrichment Score | p-value |
| --- | --- | --- | --- | --- |
| Olfactory transduction | 9 | 1202 | 0.141235 | 7.68E-18 |
| Pathways in cancer | 58 | 331 | 3.30525 | 2.00E-15 |
| Neurotrophin signaling pathway | 32 | 123 | 4.907374 | 7.47E-14 |
| Regulation of actin cytoskeleton | 43 | 219 | 3.703639 | 1.74E-13 |
| Wnt signaling pathway | 34 | 148 | 4.333328 | 6.14E-13 |
| Insulin signaling pathway | 33 | 143 | 4.352935 | 1.19E-12 |
| Metabolic pathways | 130 | 1272 | 1.927794 | 1.83E-12 |
| HIF-1 signaling pathway | 30 | 122 | 4.638374 | 2.29E-12 |
| Progesterone-mediated oocyte maturation | 25 | 86 | 5.483349 | 3.00E-12 |
| Phosphatidylinositol signaling system | 24 | 82 | 5.520796 | 7.11E-12 |
| Endocytosis | 42 | 236 | 3.356925 | 1.02E-11 |
| MAPK signaling pathway | 44 | 265 | 3.131923 | 3.58E-11 |
| Hippo signaling pathway | 32 | 159 | 3.796271 | 1.26E-10 |
| Prostate cancer | 23 | 88 | 4.930029 | 2.53E-10 |
| Focal adhesion | 36 | 208 | 3.264702 | 7.29E-10 |
| Acute myeloid leukemia | 18 | 57 | 5.956649 | 8.74E-10 |
| Pancreatic cancer | 19 | 67 | 5.34913 | 2.25E-09 |
| HTLV-I infection | 44 | 305 | 2.721179 | 3.65E-09 |
| Axon guidance | 27 | 136 | 3.744805 | 5.21E-09 |
| mTOR signaling pathway | 18 | 64 | 5.30514 | 7.10E-09 |
| Glioma | 18 | 64 | 5.30514 | 7.10E-09 |
| Endometrial cancer | 16 | 52 | 5.803914 | 1.24E-08 |
| Adherens junction | 19 | 74 | 4.843131 | 1.40E-08 |
| ErbB signaling pathway | 21 | 90 | 4.401301 | 1.48E-08 |
| Inositol phosphate metabolism | 18 | 67 | 5.067597 | 1.59E-08 |
| PI3K-Akt signaling pathway | 46 | 344 | 2.522341 | 1.82E-08 |
| Colorectal cancer | 18 | 68 | 4.993073 | 2.05E-08 |
| Circadian rhythm | 12 | 29 | 7.805264 | 2.14E-08 |
| Neuroactive ligand-receptor interaction | 41 | 290 | 2.666798 | 2.32E-08 |
| Hepatitis C | 25 | 127 | 3.713134 | 2.39E-08 |
| Chronic myeloid leukemia | 19 | 77 | 4.654438 | 2.84E-08 |
| Proteoglycans in cancer | 35 | 228 | 2.895593 | 3.24E-08 |
| Morphine addiction | 21 | 94 | 4.214012 | 3.38E-08 |
| Glutamatergic synapse | 24 | 121 | 3.741366 | 3.98E-08 |
| Cholinergic synapse | 23 | 113 | 3.839315 | 4.69E-08 |
| Epstein-Barr virus infection | 35 | 232 | 2.845669 | 5.09E-08 |
| Insulin secretion | 20 | 88 | 4.286982 | 5.40E-08 |
| Natural killer cell mediated cytotoxicity | 22 | 105 | 3.952189 | 5.41E-08 |
| T cell receptor signaling pathway | 23 | 116 | 3.740022 | 7.86E-08 |
| Influenza A | 29 | 177 | 3.090502 | 1.26E-07 |
| Transcriptional misregulation in cancer | 29 | 179 | 3.055971 | 1.62E-07 |
| Renal cell carcinoma | 17 | 69 | 4.647337 | 1.66E-07 |
| Osteoclast differentiation | 24 | 131 | 3.455766 | 1.96E-07 |
| Fc gamma R-mediated phagocytosis | 20 | 95 | 3.971099 | 2.10E-07 |
| Dopaminergic synapse | 24 | 132 | 3.429586 | 2.27E-07 |
| Calcium signaling pathway | 30 | 193 | 2.932029 | 2.51E-07 |
| Bacterial invasion of epithelial cells | 18 | 79 | 4.297835 | 2.54E-07 |
| Fc epsilon RI signaling pathway | 17 | 71 | 4.516426 | 2.61E-07 |
| Melanoma | 17 | 71 | 4.516426 | 2.61E-07 |
| Vascular smooth muscle contraction | 23 | 124 | 3.49873 | 2.84E-07 |
| Estrogen signaling pathway | 20 | 97 | 3.889221 | 3.02E-07 |
| Circadian entrainment | 20 | 98 | 3.849535 | 3.61E-07 |
| Ubiquitin mediated proteolysis | 24 | 136 | 3.328715 | 4.07E-07 |
| Type II diabetes mellitus | 14 | 51 | 5.178002 | 5.38E-07 |
| Leukocyte transendothelial migration | 22 | 119 | 3.487226 | 5.59E-07 |
| GABAergic synapse | 19 | 92 | 3.895562 | 5.97E-07 |
| Retrograde endocannabinoid signaling | 20 | 105 | 3.592899 | 1.17E-06 |
| Cell adhesion molecules (CAMs) | 27 | 175 | 2.910248 | 1.22E-06 |
| Melanogenesis | 19 | 98 | 3.657058 | 1.67E-06 |
| GnRH signaling pathway | 18 | 91 | 3.731088 | 2.37E-06 |
| Small cell lung cancer | 18 | 91 | 3.731088 | 2.37E-06 |
| Toxoplasmosis | 21 | 126 | 3.143787 | 5.96E-06 |
| Chagas disease (American trypanosomiasis) | 19 | 107 | 3.349455 | 6.64E-06 |
| Non-small cell lung cancer | 13 | 54 | 4.541025 | 7.25E-06 |
| Apoptosis | 17 | 90 | 3.562958 | 9.02E-06 |
| Gastric acid secretion | 15 | 73 | 3.875901 | 1.10E-05 |
| Viral carcinogenesis | 31 | 239 | 2.446629 | 1.28E-05 |
| Aldosterone-regulated sodium reabsorption | 11 | 41 | 5.06073 | 1.31E-05 |
| Oocyte meiosis | 19 | 114 | 3.143787 | 1.73E-05 |
| SNARE interactions in vesicular transport | 10 | 35 | 5.389349 | 1.86E-05 |
| Chemokine signaling pathway | 25 | 180 | 2.619822 | 2.12E-05 |
| B cell receptor signaling pathway | 15 | 79 | 3.581529 | 3.03E-05 |
| Long-term depression | 13 | 62 | 3.955087 | 3.65E-05 |
| Long-term potentiation | 14 | 71 | 3.71941 | 3.70E-05 |
| Protein processing in endoplasmic reticulum | 23 | 165 | 2.629349 | 4.42E-05 |
| Hepatitis B | 21 | 143 | 2.77005 | 4.48E-05 |
| Jak-STAT signaling pathway | 21 | 145 | 2.731842 | 5.54E-05 |
| Cytokine-cytokine receptor interaction | 27 | 215 | 2.368807 | 7.72E-05 |
| Measles | 20 | 140 | 2.694674 | 0.000104 |
| Amphetamine addiction | 13 | 69 | 3.553846 | 0.00012 |
| Notch signaling pathway | 11 | 51 | 4.06843 | 0.000121 |
| Endocrine and other factor-regulated calcium reabsorption | 11 | 52 | 3.990191 | 0.000146 |
| Vitamin B6 metabolism | 5 | 10 | 9.43136 | 0.000168 |
| Dorso-ventral axis formation | 7 | 22 | 6.001775 | 0.000196 |
| Herpes simplex infection | 26 | 218 | 2.249682 | 0.000216 |
| Adipocytokine signaling pathway | 13 | 73 | 3.359115 | 0.00022 |
| Thyroid cancer | 8 | 30 | 5.030059 | 0.000252 |
| Cocaine addiction | 10 | 47 | 4.013345 | 0.000293 |
| Toll-like receptor signaling pathway | 15 | 96 | 2.9473 | 0.000315 |
| Ovarian steroidogenesis | 11 | 57 | 3.640174 | 0.00035 |
| mRNA surveillance pathway | 15 | 97 | 2.916916 | 0.000355 |
| VEGF signaling pathway | 12 | 68 | 3.328715 | 0.000438 |
| Salivary secretion | 13 | 78 | 3.143787 | 0.00044 |
| Purine metabolism | 22 | 179 | 2.318323 | 0.00055 |
| RNA degradation | 13 | 81 | 3.02735 | 0.000647 |
| Serotonergic synapse | 17 | 125 | 2.56533 | 0.000667 |
| Vasopressin-regulated water reabsorption | 9 | 43 | 3.948011 | 0.000712 |
| Cell cycle | 17 | 126 | 2.54497 | 0.000734 |
| Pertussis | 12 | 72 | 3.143787 | 0.00076 |
| Lysine degradation | 10 | 53 | 3.559004 | 0.000827 |
| Bladder cancer | 8 | 37 | 4.078426 | 0.001196 |
| Alcoholism | 21 | 182 | 2.176468 | 0.001492 |
| Amoebiasis | 15 | 111 | 2.549016 | 0.001559 |
| Other types of O-glycan biosynthesis | 7 | 30 | 4.401301 | 0.001615 |
| Gap junction | 13 | 89 | 2.755229 | 0.001636 |
| Lysosome | 16 | 127 | 2.376406 | 0.002287 |
| Vitamin digestion and absorption | 6 | 24 | 4.71568 | 0.002597 |
| Tuberculosis | 21 | 186 | 2.129662 | 0.002743 |
| NF-kappa B signaling pathway | 13 | 94 | 2.608674 | 0.002744 |
| Carbohydrate digestion and absorption | 8 | 42 | 3.592899 | 0.002889 |
| Hypertrophic cardiomyopathy (HCM) | 12 | 84 | 2.694674 | 0.003114 |
| Tight junction | 17 | 138 | 2.323668 | 0.003175 |
| Arrhythmogenic right ventricular cardiomyopathy (ARVC) | 11 | 74 | 2.803918 | 0.003475 |
| Alzheimer's disease | 23 | 214 | 2.027302 | 0.003576 |
| Synaptic vesicle cycle | 10 | 64 | 2.9473 | 0.003786 |
| p53 signaling pathway | 11 | 75 | 2.766532 | 0.00388 |
| Arginine and proline metabolism | 9 | 56 | 3.031509 | 0.005156 |
| Dilated cardiomyopathy | 12 | 90 | 2.515029 | 0.005641 |
| Glycosaminoglycan biosynthesis - chondroitin sulfate / dermatan sulfate | 5 | 20 | 4.71568 | 0.006619 |
| Ribosome | 2 | 197 | 0.1915 | 0.006932 |
| Hedgehog signaling pathway | 8 | 48 | 3.143787 | 0.006972 |
| TGF-beta signaling pathway | 11 | 83 | 2.499879 | 0.008701 |
| RNA transport | 18 | 168 | 2.021006 | 0.009486 |
| N-Glycan biosynthesis | 8 | 51 | 2.958858 | 0.010227 |
| Hematopoietic cell lineage | 11 | 85 | 2.441058 | 0.010448 |
| Fructose and mannose metabolism | 7 | 42 | 3.143787 | 0.012291 |
| Alanine, aspartate and glutamate metabolism | 6 | 33 | 3.429586 | 0.014215 |
| Glycosaminoglycan biosynthesis - heparan sulfate / heparin | 5 | 24 | 3.929733 | 0.015216 |
| Basal cell carcinoma | 8 | 55 | 2.743668 | 0.016234 |
| ABC transporters | 7 | 46 | 2.870414 | 0.020303 |
| Salmonella infection | 10 | 81 | 2.328731 | 0.0209 |
| Histidine metabolism | 5 | 26 | 3.627446 | 0.021573 |
| Sphingolipid metabolism | 7 | 47 | 2.809341 | 0.02279 |
| Nucleotide excision repair | 7 | 47 | 2.809341 | 0.02279 |
| Peroxisome | 10 | 83 | 2.272617 | 0.024621 |
| Phototransduction | 5 | 27 | 3.493096 | 0.025344 |
| Selenocompound metabolism | 4 | 18 | 4.191716 | 0.026518 |
| Cysteine and methionine metabolism | 6 | 38 | 2.978324 | 0.028311 |
| Glycosphingolipid biosynthesis - lacto and neolacto series | 5 | 28 | 3.368343 | 0.029536 |
| Bile secretion | 9 | 73 | 2.325541 | 0.029692 |
| Regulation of autophagy | 5 | 29 | 3.252193 | 0.034168 |
| Nicotine addiction | 6 | 40 | 2.829408 | 0.035999 |
| Fanconi anemia pathway | 7 | 52 | 2.539212 | 0.038609 |
| beta-Alanine metabolism | 5 | 30 | 3.143787 | 0.039256 |
| Spliceosome | 14 | 141 | 1.872894 | 0.04389 |
| Cardiac muscle contraction | 10 | 86 | 2.19334 | 0.049498 |

**Table S6 Degree of Functions in miRNA-function network**

| Function Name | Degree(number of miRNAs regulating the function) |
| --- | --- |
| negative regulation of transcription from RNA polymerase II promoter | 9 |
| response to hypoxia | 9 |
| in utero embryonic development | 9 |
| amino acid transmembrane transport | 9 |
| transcription, DNA-dependent | 9 |
| regulation of transcription, DNA-dependent | 9 |
| transcription from RNA polymerase II promoter | 9 |
| protein phosphorylation | 9 |
| sodium ion transport | 9 |
| cell adhesion | 9 |
| signal transduction | 9 |
| heart development | 9 |
| aging | 9 |
| biological_process | 9 |
| response to drug | 9 |
| regulation of apoptotic process | 9 |
| negative regulation of transcription, DNA-dependent | 9 |
| positive regulation of transcription, DNA-dependent | 9 |
| positive regulation of transcription from RNA polymerase II promoter | 9 |
| protein stabilization | 9 |
| G2/M transition of mitotic cell cycle | 8 |
| angiogenesis | 8 |
| blood vessel development | 8 |
| osteoblast differentiation | 8 |
| DNA repair | 8 |
| regulation of transcription from RNA polymerase II promoter | 8 |
| protein dephosphorylation | 8 |
| proteolysis | 8 |
| intracellular protein transport | 8 |
| apoptotic process | 8 |
| induction of apoptosis | 8 |
| activation of cysteine-type endopeptidase activity involved in apoptotic process | 8 |
| cell cycle | 8 |
| cell cycle arrest | 8 |
| mitosis | 8 |
| homophilic cell adhesion | 8 |
| cell surface receptor signaling pathway | 8 |
| transforming growth factor beta receptor signaling pathway | 8 |
| G-protein coupled receptor signaling pathway | 8 |
| small GTPase mediated signal transduction | 8 |
| spermatogenesis | 8 |
| axonogenesis | 8 |
| axon guidance | 8 |
| brain development | 8 |
| learning or memory | 8 |
| circadian rhythm | 8 |
| cell proliferation | 8 |
| positive regulation of cell proliferation | 8 |
| negative regulation of cell proliferation | 8 |
| male gonad development | 8 |
| response to hormone stimulus | 8 |
| negative regulation of gene expression | 8 |
| response to organic cyclic compound | 8 |
| protein transport | 8 |
| L-amino acid transport | 8 |
| Wnt receptor signaling pathway | 8 |
| cell-cell adhesion | 8 |
| cell migration | 8 |
| protein ubiquitination | 8 |
| actin cytoskeleton organization | 8 |
| cell differentiation | 8 |
| neuron differentiation | 8 |
| forebrain development | 8 |
| neuron projection development | 8 |
| response to lipopolysaccharide | 8 |
| ion transmembrane transport | 8 |
| intracellular signal transduction | 8 |
| cilium assembly | 8 |
| odontogenesis of dentin-containing tooth | 8 |
| protein ubiquitination involved in ubiquitin-dependent protein catabolic process | 8 |
| positive regulation of apoptotic process | 8 |
| negative regulation of apoptotic process | 8 |
| proteasomal ubiquitin-dependent protein catabolic process | 8 |
| positive regulation of MAPK cascade | 8 |
| negative regulation of neuron apoptotic process | 8 |
| fat cell differentiation | 8 |
| response to ethanol | 8 |
| positive regulation of neuron differentiation | 8 |
| neuron development | 8 |
| neuron projection morphogenesis | 8 |
| cell division | 8 |
| cellular response to lipopolysaccharide | 8 |
| cellular response to hypoxia | 8 |
| negative regulation of canonical Wnt receptor signaling pathway | 8 |
| activation of MAPK activity | 7 |
| protein polyubiquitination | 7 |
| microtubule cytoskeleton organization | 7 |
| ureteric bud development | 7 |
| neuron migration | 7 |
| liver development | 7 |
| cAMP catabolic process | 7 |
| mRNA processing | 7 |
| protein complex assembly | 7 |
| ubiquitin-dependent protein catabolic process | 7 |
| transport | 7 |
| ion transport | 7 |
| chloride transport | 7 |
| cellular calcium ion homeostasis | 7 |
| exocytosis | 7 |
| endocytosis | 7 |
| autophagy | 7 |
| response to stress | 7 |
| response to DNA damage stimulus | 7 |
| response to oxidative stress | 7 |
| microtubule-based movement | 7 |
| cell communication | 7 |
| elevation of cytosolic calcium ion concentration | 7 |
| intracellular protein kinase cascade | 7 |
| nervous system development | 7 |
| synapse assembly | 7 |
| blood coagulation | 7 |
| memory | 7 |
| protein localization | 7 |
| insulin receptor signaling pathway | 7 |
| visual learning | 7 |
| response to heat | 7 |
| response to glucose stimulus | 7 |
| embryo development | 7 |
| positive regulation of gene expression | 7 |
| vesicle-mediated transport | 7 |
| dephosphorylation | 7 |
| lamellipodium assembly | 7 |
| positive regulation of Wnt receptor signaling pathway | 7 |
| lung development | 7 |
| positive regulation of cell migration | 7 |
| cellular response to insulin stimulus | 7 |
| response to cytokine stimulus | 7 |
| phosphatidylinositol-3-phosphate biosynthetic process | 7 |
| negative regulation of protein catabolic process | 7 |
| positive regulation of neuron apoptotic process | 7 |
| positive regulation of GTPase activity | 7 |
| response to estrogen stimulus | 7 |
| positive regulation of protein kinase activity | 7 |
| positive regulation of vasoconstriction | 7 |
| protein autophosphorylation | 7 |
| phosphatidylinositol-mediated signaling | 7 |
| positive regulation of smooth muscle cell proliferation | 7 |
| stem cell differentiation | 7 |
| protein homooligomerization | 7 |
| neuron apoptotic process | 7 |
| response to calcium ion | 7 |
| iron ion homeostasis | 7 |
| canonical Wnt receptor signaling pathway | 7 |
| positive regulation of ERK1 and ERK2 cascade | 7 |
| cellular response to tumor necrosis factor | 7 |
| autophagic vacuole assembly | 6 |
| negative regulation of protein phosphorylation | 6 |
| positive regulation of cell-matrix adhesion | 6 |
| mRNA polyadenylation | 6 |
| cellular protein modification process | 6 |
| protein O-linked glycosylation | 6 |
| dopamine receptor signaling pathway | 6 |
| Notch signaling pathway | 6 |
| JNK cascade | 6 |
| multicellular organismal development | 6 |
| spermatid development | 6 |
| associative learning | 6 |
| regulation of cell shape | 6 |
| post-embryonic development | 6 |
| organ morphogenesis | 6 |
| response to inorganic substance | 6 |
| regulation of gene expression | 6 |
| regulation of exocytosis | 6 |
| sensory perception of pain | 6 |
| cAMP-mediated signaling | 6 |
| central nervous system projection neuron axonogenesis | 6 |
| cerebral cortex development | 6 |
| SCF-dependent proteasomal ubiquitin-dependent protein catabolic process | 6 |
| positive regulation of insulin secretion | 6 |
| negative regulation of NF-kappaB transcription factor activity | 6 |
| regulation of ARF GTPase activity | 6 |
| response to retinoic acid | 6 |
| positive regulation of peptidyl-serine phosphorylation | 6 |
| multicellular organism growth | 6 |
| sodium ion transmembrane transport | 6 |
| termination of G-protein coupled receptor signaling pathway | 6 |
| regulation of cell proliferation | 6 |
| cellular response to glucose starvation | 6 |
| response to morphine | 6 |
| cell fate commitment | 6 |
| positive regulation of fat cell differentiation | 6 |
| positive regulation of glycolysis | 6 |
| positive regulation of glucose import | 6 |
| negative regulation of insulin secretion | 6 |
| insulin-like growth factor receptor signaling pathway | 6 |
| regulation of synaptic plasticity | 6 |
| embryonic skeletal system morphogenesis | 6 |
| brain morphogenesis | 6 |
| positive regulation of epithelial cell proliferation | 6 |
| positive regulation of protein kinase B signaling cascade | 6 |
| transmembrane transport | 6 |
| response to interleukin-1 | 6 |
| cellular response to amino acid stimulus | 6 |
| cellular response to interleukin-1 | 6 |
| G1/S transition of mitotic cell cycle | 5 |
| ovarian follicle development | 5 |
| ameboidal cell migration | 5 |
| gastrulation with mouth forming second | 5 |
| histone mRNA 3'-end processing | 5 |
| membrane protein ectodomain proteolysis | 5 |
| cation transport | 5 |
| calcium ion transport | 5 |
| adenylate cyclase-modulating G-protein coupled receptor signaling pathway | 5 |
| phospholipase C-activating G-protein coupled receptor signaling pathway | 5 |
| integrin-mediated signaling pathway | 5 |
| activation of JUN kinase activity | 5 |
| synaptic transmission, cholinergic | 5 |
| germ cell migration | 5 |
| response to toxic substance | 5 |
| regulation of autophagy | 5 |
| histone methylation | 5 |
| Rac protein signal transduction | 5 |
| peptidyl-serine phosphorylation | 5 |
| peptidyl-tyrosine phosphorylation | 5 |
| cerebral cortex radially oriented cell migration | 5 |
| keratinocyte differentiation | 5 |
| negative regulation of ossification | 5 |
| negative regulation of cell growth | 5 |
| positive regulation of transforming growth factor beta receptor signaling pathway | 5 |
| negative regulation of transforming growth factor beta receptor signaling pathway | 5 |
| negative regulation of protein binding | 5 |
| positive regulation of Rac GTPase activity | 5 |
| cellular protein localization | 5 |
| histone lysine methylation | 5 |
| response to nicotine | 5 |
| hippo signaling cascade | 5 |
| positive regulation of renal sodium excretion | 5 |
| megakaryocyte development | 5 |
| regulation of vasodilation | 5 |
| myelination | 5 |
| negative regulation of vascular permeability | 5 |
| negative regulation of cysteine-type endopeptidase activity involved in apoptotic process | 5 |
| positive regulation of JUN kinase activity | 5 |
| negative regulation of mitotic cell cycle | 5 |
| negative regulation of JNK cascade | 5 |
| phosphatidylinositol metabolic process | 5 |
| axon extension | 5 |
| branching morphogenesis of an epithelial tube | 5 |
| dendrite morphogenesis | 5 |
| leukemia inhibitory factor signaling pathway | 5 |
| positive regulation of peptidyl-tyrosine phosphorylation | 5 |
| negative chemotaxis | 5 |
| actin filament bundle assembly | 5 |
| positive regulation of synaptic transmission, glutamatergic | 5 |
| regulation of excitatory postsynaptic membrane potential | 5 |
| ciliary neurotrophic factor-mediated signaling pathway | 5 |
| calcium ion transmembrane transport | 5 |
| cellular response to glucose stimulus | 5 |
| regulation of cell growth | 4 |
| lymph vessel development | 4 |
| glycogen metabolic process | 4 |
| mRNA splice site selection | 4 |
| Golgi to endosome transport | 4 |
| cellular membrane fusion | 4 |
| humoral immune response | 4 |
| female meiosis | 4 |
| epidermal growth factor receptor signaling pathway | 4 |
| adenylate cyclase-inhibiting G-protein coupled receptor signaling pathway | 4 |
| protein kinase C-activating G-protein coupled receptor signaling pathway | 4 |
| gastrulation | 4 |
| learning | 4 |
| L-glutamate transport | 4 |
| ventricular system development | 4 |
| hemopoiesis | 4 |
| regulation of cell adhesion | 4 |
| negative regulation of Wnt receptor signaling pathway | 4 |
| chondroitin sulfate biosynthetic process | 4 |
| regulation of cell migration | 4 |
| negative regulation of protein complex assembly | 4 |
| response to estradiol stimulus | 4 |
| negative regulation of interleukin-8 production | 4 |
| circadian regulation of gene expression | 4 |
| cytoplasmic mRNA processing body assembly | 4 |
| skeletal muscle cell differentiation | 4 |
| positive regulation of DNA replication | 4 |
| positive regulation of mitosis | 4 |
| positive regulation of JAK-STAT cascade | 4 |
| regulation of organ growth | 4 |
| carbohydrate phosphorylation | 4 |
| filopodium assembly | 4 |
| phosphatidylinositol phosphorylation | 4 |
| platelet-derived growth factor receptor signaling pathway | 4 |
| behavioral response to ethanol | 4 |
| embryonic viscerocranium morphogenesis | 4 |
| positive regulation of stress fiber assembly | 4 |
| cell chemotaxis | 4 |
| cellular response to transforming growth factor beta stimulus | 4 |
| regulation of heart rate by cardiac conduction | 4 |
| positive regulation of excitatory postsynaptic membrane potential | 4 |
| regulation of cell-matrix adhesion | 3 |
| endocardium development | 3 |
| ER to Golgi vesicle-mediated transport | 3 |
| response to manganese ion | 3 |
| positive regulation of cardiac muscle hypertrophy | 3 |
| positive regulation of triglyceride biosynthetic process | 3 |
| peptidyl-threonine phosphorylation | 3 |
| regulation of cell morphogenesis | 3 |
| positive regulation of intracellular estrogen receptor signaling pathway | 3 |
| cellular triglyceride homeostasis | 3 |
| ovulation cycle | 3 |
| regulation of GTPase activity | 3 |
| positive regulation of vascular permeability | 3 |
| positive regulation of insulin-like growth factor receptor signaling pathway | 3 |
| negative regulation of translational initiation | 3 |
| lipid phosphorylation | 3 |
| somatic stem cell division | 3 |
| paraxial mesoderm formation | 3 |
| embryonic hindgut morphogenesis | 3 |
| regulation of phagocytosis | 3 |
| reduction of cytosolic calcium ion concentration | 3 |
| elevation of cytosolic calcium ion concentration involved in phospholipase C-activating G-protein coupled signaling pathway | 3 |
| centrosome localization | 3 |
| smooth muscle contraction involved in micturition | 3 |
| desensitization of G-protein coupled receptor protein signaling pathway | 2 |
| fructose 2,6-bisphosphate metabolic process | 2 |
| translation | 2 |
| JUN phosphorylation | 2 |
| positive regulation of mammary gland epithelial cell proliferation | 2 |
| negative regulation of smooth muscle cell apoptotic process | 2 |
| clustering of voltage-gated sodium channels | 2 |
| positive regulation of sarcomere organization | 2 |
| salivary gland cavitation | 2 |
| cellular response to electrical stimulus | 2 |
| detection of chemical stimulus involved in sensory perception of smell | 1 |

**Table S7 Degree of target genes in miRNA-gene network**

| Gene Symbol | Degree (the number of miRNAs modulating the target genes) |
| --- | --- |
| Bcl2l1 | 5 |
| Arpp19 | 4 |
| Cpsf7 | 4 |
| Il34 | 4 |
| Lasp1 | 4 |
| Map4 | 4 |
| Mdm4 | 4 |
| Mipep | 4 |
| Mpzl2 | 4 |
| Ndrg1 | 4 |
| Rtkn2 | 4 |
| Slc38a1 | 4 |
| Tfcp2l1 | 4 |
| Wasf2 | 4 |
| Zmat3 | 4 |
| Agap1 | 3 |
| Bcl2l2 | 3 |
| Celf1 | 3 |
| Clock | 3 |
| Ddi2 | 3 |
| Deptor | 3 |
| Dgcr2 | 3 |
| Disc1 | 3 |
| Enc1 | 3 |
| Epha7 | 3 |
| Ermp1 | 3 |
| Fancc | 3 |
| Fktn | 3 |
| Gas2l1 | 3 |
| Gas7 | 3 |
| Gdnf | 3 |
| Glp1r | 3 |
| Has3 | 3 |
| Ifit2 | 3 |
| Kif9 | 3 |
| Klf16 | 3 |
| LOC100910212 | 3 |
| Lsm11 | 3 |
| Mecom | 3 |
| Mtf1 | 3 |
| Nfat5 | 3 |
| Nlrp3 | 3 |
| Nr4a2 | 3 |
| Pde1c | 3 |
| Phex | 3 |
| Pip4k2a | 3 |
| Podxl | 3 |
| Pofut1 | 3 |
| Ppara | 3 |
| Psd3 | 3 |
| Pura | 3 |
| Rab7b | 3 |
| Rgs3 | 3 |
| Rspo2 | 3 |
| Sfxn5 | 3 |
| Slc4a1 | 3 |
| Soga1 | 3 |
| Spred3 | 3 |
| Stk3 | 3 |
| Supt3h | 3 |
| Tab2 | 3 |
| Tbl1x | 3 |
| Tmem237 | 3 |
| Tpm3 | 3 |
| Twist2 | 3 |
| Ube2i | 3 |
| Usp49 | 3 |
| Zfp219 | 3 |
| Zfp488 | 3 |
| Zfp518b | 3 |
| Abca2 | 2 |
| Abcb9 | 2 |
| Adam12 | 2 |
| Adam32 | 2 |
| Adamts4 | 2 |
| Adcy6 | 2 |
| Adora1 | 2 |
| Adora2a | 2 |
| Adra2c | 2 |
| Ahcyl1 | 2 |
| Aipl1 | 2 |
| Akap13 | 2 |
| Amigo1 | 2 |
| Ankrd12 | 2 |
| Ap1b1 | 2 |
| Apba3 | 2 |
| Appbp2 | 2 |
| Arfip2 | 2 |
| Arhgap30 | 2 |
| Arhgef7 | 2 |
| Arnt2 | 2 |
| Arrb1 | 2 |
| Asah1 | 2 |
| Atg10 | 2 |
| Atg14 | 2 |
| Atxn2 | 2 |
| Brf1 | 2 |
| C3ar1 | 2 |
| Cacna1d | 2 |
| Cacna2d2 | 2 |
| Camkk1 | 2 |
| Camlg | 2 |
| Capn1 | 2 |
| Caskin1 | 2 |
| Cbl | 2 |
| Ccdc85b | 2 |
| Ccnc | 2 |
| Ccnt1 | 2 |
| Cd4 | 2 |
| Cdh16 | 2 |
| Cdkl5 | 2 |
| Cdkn2b | 2 |
| Chek2 | 2 |
| Chmp6 | 2 |
| Clstn2 | 2 |
| Cltc | 2 |
| Cobl | 2 |
| Col17a1 | 2 |
| Coro2b | 2 |
| Cox15 | 2 |
| Cplx3 | 2 |
| Creb1 | 2 |
| Crk | 2 |
| Csnk1g3 | 2 |
| Cspg4 | 2 |
| Cstf1 | 2 |
| Ctnnbip1 | 2 |
| Cxcl12 | 2 |
| Cyp26c1 | 2 |
| Daam2 | 2 |
| Dapk2 | 2 |
| Dclk1 | 2 |
| Dkk3 | 2 |
| Dlg5 | 2 |
| Dlgap3 | 2 |
| Dlx1 | 2 |
| Dnajc5 | 2 |
| Dnm3 | 2 |
| Doc2b | 2 |
| Dot1l | 2 |
| Dusp15 | 2 |
| Dusp9 | 2 |
| E2f7 | 2 |
| Ece1 | 2 |
| Efemp1 | 2 |
| Eid2 | 2 |
| Elf1 | 2 |
| Elf2 | 2 |
| Elmod2 | 2 |
| Emx1 | 2 |
| Eny2 | 2 |
| Epha8 | 2 |
| Eqtn | 2 |
| Etv3 | 2 |
| Fbxl14 | 2 |
| Fbxl7 | 2 |
| Fbxw11 | 2 |
| Fcamr | 2 |
| Fgf1 | 2 |
| Fip1l1 | 2 |
| Fndc3a | 2 |
| Foxc1 | 2 |
| Foxc2 | 2 |
| Foxp1 | 2 |
| Frs2 | 2 |
| Furin | 2 |
| Fzd8 | 2 |
| Gck | 2 |
| Gclm | 2 |
| Gdf7 | 2 |
| Gfpt1 | 2 |
| Gipc1 | 2 |
| Glis2 | 2 |
| Gls2 | 2 |
| Gpn2 | 2 |
| Gpr161 | 2 |
| Gpr35 | 2 |
| Gpr55 | 2 |
| Grhl2 | 2 |
| Hdac9 | 2 |
| Hecw2 | 2 |
| Hnrnpu | 2 |
| Hook1 | 2 |
| Hoxb3 | 2 |
| Hsf1 | 2 |
| Igf1r | 2 |
| Il6r | 2 |
| Inpp5e | 2 |
| Irf1 | 2 |
| Kctd2 | 2 |
| Kif13b | 2 |
| Kif16b | 2 |
| Klhl17 | 2 |
| Ksr2 | 2 |
| Lamb3 | 2 |
| Lamc2 | 2 |
| Lgals8 | 2 |
| Lif | 2 |
| Lifr | 2 |
| Lig3 | 2 |
| Lmln | 2 |
| LOC100910977 | 2 |
| LOC499770 | 2 |
| LOC691920 | 2 |
| Lrat | 2 |
| Lrrc16a | 2 |
| Lrrc55 | 2 |
| Mafg | 2 |
| Map1a | 2 |
| Map3k11 | 2 |
| Mapk13 | 2 |
| Marf1 | 2 |
| Max | 2 |
| Mdfic | 2 |
| Mef2c | 2 |
| Mfsd9 | 2 |
| MGC109340 | 2 |
| Mmaa | 2 |
| Mmp17 | 2 |
| Mov10l1 | 2 |
| Mrc2 | 2 |
| Msx3 | 2 |
| Mtbp | 2 |
| Mtf2 | 2 |
| Naa60 | 2 |
| Nlgn3 | 2 |
| Nol3 | 2 |
| Nsmce2 | 2 |
| Ntrk3 | 2 |
| Oprk1 | 2 |
| Orai2 | 2 |
| Otog | 2 |
| Otub1 | 2 |
| P2ry2 | 2 |
| Pafah1b2 | 2 |
| Papss2 | 2 |
| Pcdhga1 | 2 |
| Pcdhga11 | 2 |
| Pcdhga12 | 2 |
| Pcdhga2 | 2 |
| Pcdhga3 | 2 |
| Pcdhga5 | 2 |
| Pcdhga7 | 2 |
| Pcdhga8 | 2 |
| Pcdhga9 | 2 |
| Pcdhgb7 | 2 |
| Pcdhgb8 | 2 |
| Pcdhgc3 | 2 |
| Pdgfra | 2 |
| Pdpk1 | 2 |
| Pdxp | 2 |
| Pex19 | 2 |
| Pfkfb2 | 2 |
| Pfkfb3 | 2 |
| Pik3cd | 2 |
| Pip4k2b | 2 |
| Pitpnc1 | 2 |
| Pkn2 | 2 |
| Plch1 | 2 |
| Plscr2 | 2 |
| Pom121 | 2 |
| Pou3f3 | 2 |
| Ppp2r2d | 2 |
| Prc1 | 2 |
| Prkd3 | 2 |
| Prss35 | 2 |
| Prune | 2 |
| Psmg2 | 2 |
| Ptbp2 | 2 |
| Ptpn4 | 2 |
| Ptprb | 2 |
| Ptprd | 2 |
| Ptprk | 2 |
| Ptprt | 2 |
| Pxk | 2 |
| Rab27a | 2 |
| Ralgds | 2 |
| Raph1 | 2 |
| Rassf2 | 2 |
| Rc3h1 | 2 |
| Rdh12 | 2 |
| Rela | 2 |
| RGD1564379 | 2 |
| Rgl1 | 2 |
| Ric3 | 2 |
| Rnf114 | 2 |
| Rnf8 | 2 |
| Rprm | 2 |
| Rraga | 2 |
| Rtn4rl1 | 2 |
| Sarm1 | 2 |
| Scarb2 | 2 |
| Scin | 2 |
| Scnn1a | 2 |
| Sdad1 | 2 |
| 6-Sep | 2 |
| 8-Sep | 2 |
| Sh3bp2 | 2 |
| Shisa5 | 2 |
| Slbp | 2 |
| Slc11a2 | 2 |
| Slc12a6 | 2 |
| Slc25a13 | 2 |
| Slc25a54 | 2 |
| Slc38a3 | 2 |
| Slc44a1 | 2 |
| Slc7a5 | 2 |
| Slco2a1 | 2 |
| Smoc1 | 2 |
| Snai3 | 2 |
| Snph | 2 |
| Snx18 | 2 |
| Snx30 | 2 |
| Spns2 | 2 |
| Spock2 | 2 |
| Sppl2b | 2 |
| Spry4 | 2 |
| Sptb | 2 |
| Srgap2 | 2 |
| Ston2 | 2 |
| Stx1a | 2 |
| Stx8 | 2 |
| Sv2a | 2 |
| Sytl4 | 2 |
| Taf9b | 2 |
| Tbl1xr1 | 2 |
| Tceal8 | 2 |
| Tcf7 | 2 |
| Tcp11 | 2 |
| Tdrd12 | 2 |
| Tfrc | 2 |
| Thbd | 2 |
| Timeless | 2 |
| Tmem128 | 2 |
| Tmem18 | 2 |
| Tmprss2 | 2 |
| Tnpo1 | 2 |
| Tomm40l | 2 |
| Tp53inp1 | 2 |
| Trpv3 | 2 |
| Ttll9 | 2 |
| Tub | 2 |
| Ubqln1 | 2 |
| Uevld | 2 |
| Unc79 | 2 |
| Uncx | 2 |
| Vamp2 | 2 |
| Vapb | 2 |
| Vezf1 | 2 |
| Vldlr | 2 |
| Wdr6 | 2 |
| Zfp236 | 2 |
| Zfp385a | 2 |
| Zfp862 | 2 |
| Zfyve27 | 2 |
| Zhx3 | 2 |
| A1cf | 1 |
| Aak1 | 1 |
| Abca8a | 1 |
| Abcb11 | 1 |
| Abcc8 | 1 |
| Abcd3 | 1 |
| Abl2 | 1 |
| Ablim1 | 1 |
| Acap2 | 1 |
| Acer2 | 1 |
| Acsl6 | 1 |
| Acvr1b | 1 |
| Adam17 | 1 |
| Adam19 | 1 |
| Adam30 | 1 |
| Adamts1 | 1 |
| Adamts3 | 1 |
| Adar | 1 |
| Adcy9 | 1 |
| Adcyap1r1 | 1 |
| Adipor1 | 1 |
| Adk | 1 |
| Adra1a | 1 |
| Adrbk1 | 1 |
| Adrbk2 | 1 |
| Adss | 1 |
| Aes | 1 |
| Agap2 | 1 |
| Agap3 | 1 |
| Agfg1 | 1 |
| Agk | 1 |
| Ago1 | 1 |
| Agtr1a | 1 |
| Aida | 1 |
| Aifm2 | 1 |
| Ajuba | 1 |
| Akap10 | 1 |
| Akt1s1 | 1 |
| Alg1 | 1 |
| Alg9 | 1 |
| Alkbh1 | 1 |
| Alkbh3 | 1 |
| Als2 | 1 |
| Amot | 1 |
| Amotl1 | 1 |
| Amt | 1 |
| Anapc11 | 1 |
| Angpt4 | 1 |
| Angptl3 | 1 |
| Ankrd54 | 1 |
| Ano1 | 1 |
| Ano6 | 1 |
| Ap1s1 | 1 |
| Apaf1 | 1 |
| Apba1 | 1 |
| Apc2 | 1 |
| Apold1 | 1 |
| Aqp3 | 1 |
| Aqp4 | 1 |
| Araf | 1 |
| Arap2 | 1 |
| Arel1 | 1 |
| Arf2 | 1 |
| Arf3 | 1 |
| Arfgap1 | 1 |
| Arfgap2 | 1 |
| Arfrp1 | 1 |
| Arhgap29 | 1 |
| Arhgap42 | 1 |
| Arhgap9 | 1 |
| Arhgef39 | 1 |
| Arhgef4 | 1 |
| Arhgef5 | 1 |
| Arl10 | 1 |
| Arl4a | 1 |
| Arl8b | 1 |
| Asb2 | 1 |
| Ascc3 | 1 |
| Ash2l | 1 |
| Atcay | 1 |
| Atf6 | 1 |
| Atg13 | 1 |
| Atg7 | 1 |
| Atg9a | 1 |
| Atl2 | 1 |
| Atp13a3 | 1 |
| Atp1b1 | 1 |
| Atp2b3 | 1 |
| Atpaf1 | 1 |
| Atrn | 1 |
| Atrx | 1 |
| Atxn7l4 | 1 |
| Avl9 | 1 |
| Avpr1b | 1 |
| B3galt5 | 1 |
| Bag4 | 1 |
| Baiap2 | 1 |
| Bbs1 | 1 |
| Bche | 1 |
| Bcl11a | 1 |
| Bcl2l11 | 1 |
| Bcl6b | 1 |
| Bdkrb2 | 1 |
| Best3 | 1 |
| Bet1 | 1 |
| Bfar | 1 |
| Bhlhe22 | 1 |
| Bicd2 | 1 |
| Blmh | 1 |
| Bloc1s2 | 1 |
| Bmf | 1 |
| Bmp2k | 1 |
| Bmp7 | 1 |
| Bnip1 | 1 |
| Bnip3l | 1 |
| Braf | 1 |
| Brd2 | 1 |
| Brd3 | 1 |
| Brwd1 | 1 |
| Bsnd | 1 |
| Btbd9 | 1 |
| Btla | 1 |
| C1qtnf1 | 1 |
| C1qtnf3 | 1 |
| Cacna1c | 1 |
| Cactin | 1 |
| Cacul1 | 1 |
| Cacybp | 1 |
| Calm2 | 1 |
| Cask | 1 |
| Casp2 | 1 |
| Casp3 | 1 |
| Casp7 | 1 |
| Cbx6 | 1 |
| Ccdc91 | 1 |
| Ccnd1 | 1 |
| Cd44 | 1 |
| Cdc23 | 1 |
| Cdc25a | 1 |
| Cdc25c | 1 |
| Cdc42 | 1 |
| Cdc7 | 1 |
| Cdh1 | 1 |
| Cdh12 | 1 |
| Cdh22 | 1 |
| Cdip1 | 1 |
| Cdk14 | 1 |
| Cdk19 | 1 |
| Cebpa | 1 |
| Celsr2 | 1 |
| Cenpv | 1 |
| Cerk | 1 |
| Cftr | 1 |
| Chd2 | 1 |
| Chmp4c | 1 |
| Chrna5 | 1 |
| Chrna7 | 1 |
| Chst7 | 1 |
| Chsy1 | 1 |
| Ciapin1 | 1 |
| Ciita | 1 |
| Cks1b | 1 |
| Clasp2 | 1 |
| Clcn3 | 1 |
| Clcn4 | 1 |
| Clgn | 1 |
| Clic3 | 1 |
| Clic5 | 1 |
| Cmklr1 | 1 |
| Cml3 | 1 |
| Cndp1 | 1 |
| Cnga3 | 1 |
| Cngb1 | 1 |
| Cnot2 | 1 |
| Col16a1 | 1 |
| Col4a3bp | 1 |
| Col4a4 | 1 |
| Copg1 | 1 |
| Coro1c | 1 |
| Cpa4 | 1 |
| Cpeb1 | 1 |
| Cpsf2 | 1 |
| Cpsf6 | 1 |
| Cpt1a | 1 |
| Creb5 | 1 |
| Crebl2 | 1 |
| Creg1 | 1 |
| Crim1 | 1 |
| Crmp1 | 1 |
| Cryz | 1 |
| Csf1 | 1 |
| Csnk1g1 | 1 |
| Csnk2a1 | 1 |
| Csnk2a2 | 1 |
| Ctgf | 1 |
| Ctnnal1 | 1 |
| Ctnnd2 | 1 |
| Ctsk | 1 |
| Cul1 | 1 |
| Cul4b | 1 |
| Cul5 | 1 |
| Cxxc4 | 1 |
| Cyp2w1 | 1 |
| Cyp7a1 | 1 |
| Cysltr1 | 1 |
| Cyth3 | 1 |
| Cytip | 1 |
| Cytl1 | 1 |
| Dact1 | 1 |
| Dap | 1 |
| Dbnl | 1 |
| Dcaf17 | 1 |
| Dcdc2c | 1 |
| Dcx | 1 |
| Ddb1 | 1 |
| Ddc | 1 |
| Ddx5 | 1 |
| Ddx6 | 1 |
| Depdc1b | 1 |
| Desi2 | 1 |
| Dgat2 | 1 |
| Dgcr8 | 1 |
| Dgkg | 1 |
| Dgkh | 1 |
| Dgkz | 1 |
| Dhh | 1 |
| Diras1 | 1 |
| Dirc2 | 1 |
| Dixdc1 | 1 |
| Dnah1 | 1 |
| Dnaja3 | 1 |
| Dnhd1-ps1 | 1 |
| Dnmt3a | 1 |
| Dock3 | 1 |
| Dock5 | 1 |
| Dock9 | 1 |
| Drd2 | 1 |
| Dsc2 | 1 |
| Dsg2 | 1 |
| Dspp | 1 |
| Dtx3l | 1 |
| Duox2 | 1 |
| Dusp16 | 1 |
| Dvl2 | 1 |
| E2f1 | 1 |
| Eaf1 | 1 |
| Ebf3 | 1 |
| Eda | 1 |
| Edem3 | 1 |
| Edn1 | 1 |
| Eef1e1 | 1 |
| Egfr | 1 |
| Eif4e | 1 |
| Eif4ebp1 | 1 |
| Ell3 | 1 |
| Eno2 | 1 |
| Eogt | 1 |
| Epc1 | 1 |
| Epha2 | 1 |
| Epo | 1 |
| Eps8 | 1 |
| Erap1 | 1 |
| Ercc4 | 1 |
| Ercc8 | 1 |
| Ergic1 | 1 |
| Esrp2 | 1 |
| Ets1 | 1 |
| Ets2 | 1 |
| Exoc8 | 1 |
| Eya1 | 1 |
| F2rl3 | 1 |
| Fam107a | 1 |
| Fam110c | 1 |
| Fam26e | 1 |
| Fam3d | 1 |
| Fam63b | 1 |
| Fan1 | 1 |
| Fancl | 1 |
| Fas | 1 |
| Fat2 | 1 |
| Fbln5 | 1 |
| Fbxl5 | 1 |
| Fbxo4 | 1 |
| Fech | 1 |
| Fez2 | 1 |
| Fgd4 | 1 |
| Fhod1 | 1 |
| Fitm2 | 1 |
| Flnb | 1 |
| Fn1 | 1 |
| Fndc3b | 1 |
| Fosb | 1 |
| Foxj2 | 1 |
| Foxm1 | 1 |
| Foxo4 | 1 |
| Fshr | 1 |
| Fst | 1 |
| Fzd10 | 1 |
| Fzd7 | 1 |
| Gab1 | 1 |
| Gab2 | 1 |
| Gabarapl2 | 1 |
| Gabrb1 | 1 |
| Galr1 | 1 |
| Gas2 | 1 |
| Gas2l3 | 1 |
| Gatad2a | 1 |
| Gclc | 1 |
| Gcm2 | 1 |
| Gdap1 | 1 |
| Gdf10 | 1 |
| Gdi2 | 1 |
| Gfra1 | 1 |
| Gga1 | 1 |
| Ghrhr | 1 |
| Gimap5 | 1 |
| Gipr | 1 |
| Gja5 | 1 |
| Gjb3 | 1 |
| Glg1 | 1 |
| Glra2 | 1 |
| Glul | 1 |
| Gmnc | 1 |
| Gna12 | 1 |
| Gnai3 | 1 |
| Gnao1 | 1 |
| Gng4 | 1 |
| Gnptab | 1 |
| Got2 | 1 |
| Gpr116 | 1 |
| Gpr56 | 1 |
| Gpx5 | 1 |
| Gramd1b | 1 |
| Grb10 | 1 |
| Grin2a | 1 |
| Grk5 | 1 |
| Gsk3b | 1 |
| Gtf2e1 | 1 |
| Guca1b | 1 |
| Gucy1b2 | 1 |
| Gucy2g | 1 |
| Hace1 | 1 |
| Haus1 | 1 |
| Hbp1 | 1 |
| Hck | 1 |
| Hectd2 | 1 |
| Herc6 | 1 |
| Heyl | 1 |
| Higd1a | 1 |
| Hinfp | 1 |
| Hipk1 | 1 |
| Hipk2 | 1 |
| Hipk3 | 1 |
| Hira | 1 |
| Hivep3 | 1 |
| Hk2 | 1 |
| Hmbox1 | 1 |
| Hoxa11 | 1 |
| Hoxa2 | 1 |
| Hoxd4 | 1 |
| Hps3 | 1 |
| Hps4 | 1 |
| Hs1bp3 | 1 |
| Hs3st2 | 1 |
| Hs6st1 | 1 |
| Hspa12a | 1 |
| Hspa5 | 1 |
| Hspa9 | 1 |
| Hspd1 | 1 |
| Htra1 | 1 |
| Htra4 | 1 |
| Htt | 1 |
| Icam1 | 1 |
| Ids | 1 |
| Ifngr1 | 1 |
| Ift140 | 1 |
| Igf1 | 1 |
| Igfbp4 | 1 |
| Il12rb2 | 1 |
| Il6st | 1 |
| Impa2 | 1 |
| Impad1 | 1 |
| Inpp4a | 1 |
| Insl3 | 1 |
| Insr | 1 |
| Ip6k2 | 1 |
| Ipo4 | 1 |
| Iqgap3 | 1 |
| Irak1 | 1 |
| Irak3 | 1 |
| Ireb2 | 1 |
| Irf6 | 1 |
| Irs2 | 1 |
| Irx4 | 1 |
| Itch | 1 |
| Itga1 | 1 |
| Itga8 | 1 |
| Itgad | 1 |
| Itgal | 1 |
| Itpr2 | 1 |
| Jazf1 | 1 |
| Jdp2 | 1 |
| Josd1 | 1 |
| Kank2 | 1 |
| Katnal1 | 1 |
| Kcnd1 | 1 |
| Kcne4 | 1 |
| Kcnk1 | 1 |
| Kcnmb1 | 1 |
| Kctd10 | 1 |
| Kctd20 | 1 |
| Kctd4 | 1 |
| Kctd5 | 1 |
| Kdm8 | 1 |
| Kera | 1 |
| Kif18b | 1 |
| Kif20b | 1 |
| Kif27 | 1 |
| Kif3c | 1 |
| Kirrel | 1 |
| Kit | 1 |
| Klf12 | 1 |
| Klf5 | 1 |
| Klf7 | 1 |
| Klhl12 | 1 |
| Klhl21 | 1 |
| Kmt2d | 1 |
| Ktn1 | 1 |
| Lamp1 | 1 |
| Lats1 | 1 |
| Lats2 | 1 |
| Lcorl | 1 |
| Lcp1 | 1 |
| Ldb3 | 1 |
| Ldlrap1 | 1 |
| Lfng | 1 |
| Lhpp | 1 |
| Limd1 | 1 |
| Lin28a | 1 |
| Lipi | 1 |
| Lix1l | 1 |
| Llgl1 | 1 |
| LOC100360606 | 1 |
| LOC100909998 | 1 |
| LOC100910318 | 1 |
| LOC100910823 | 1 |
| LOC100911440 | 1 |
| LOC100911794 | 1 |
| LOC100911837 | 1 |
| LOC683422 | 1 |
| LOC689629 | 1 |
| LOC691422 | 1 |
| Lpar1 | 1 |
| Lpp | 1 |
| Lppr4 | 1 |
| Lrch4 | 1 |
| Lrrc26 | 1 |
| Lrsam1 | 1 |
| Lsamp | 1 |
| Lsm14a | 1 |
| Ltf | 1 |
| Ly6e | 1 |
| Mab21l1 | 1 |
| Maged1 | 1 |
| Maob | 1 |
| Map2k2 | 1 |
| Map2k7 | 1 |
| Mapk10 | 1 |
| Mapk8ip3 | 1 |
| Mapk9 | 1 |
| Mapkap1 | 1 |
| Mapt | 1 |
| 1-Mar | 1 |
| 10-Mar | 1 |
| Mat1a | 1 |
| Mbtps2 | 1 |
| Mea1 | 1 |
| Mecp2 | 1 |
| Med17 | 1 |
| Med23 | 1 |
| Med26 | 1 |
| Mef2a | 1 |
| Mef2d | 1 |
| Meis1 | 1 |
| Meox1 | 1 |
| Metap1 | 1 |
| Mgat4a | 1 |
| Mme | 1 |
| Mmgt1 | 1 |
| Mmgt2 | 1 |
| Mmp14 | 1 |
| Mob1a | 1 |
| Mos | 1 |
| Mrpl12 | 1 |
| Mrps18b | 1 |
| Mta2 | 1 |
| Mta3 | 1 |
| Mthfr | 1 |
| Mthfs | 1 |
| Mtmr1 | 1 |
| Mtus1 | 1 |
| Muc15 | 1 |
| Muc20 | 1 |
| Mum1 | 1 |
| Mxi1 | 1 |
| Myf5 | 1 |
| Mylk | 1 |
| Mylk3 | 1 |
| Myo1e | 1 |
| Myo5b | 1 |
| Myrip | 1 |
| Nab2 | 1 |
| Nabp2 | 1 |
| Nanos1 | 1 |
| Ncam2 | 1 |
| Ncan | 1 |
| Ncoa1 | 1 |
| Ncoa4 | 1 |
| Ndel1 | 1 |
| Ndrg2 | 1 |
| Ndst1 | 1 |
| Ndufa10 | 1 |
| Ndufaf4 | 1 |
| Nek1 | 1 |
| Nek11 | 1 |
| Nek7 | 1 |
| Neo1 | 1 |
| Neto1 | 1 |
| Neurod2 | 1 |
| Nexn | 1 |
| Nf2 | 1 |
| Nfasc | 1 |
| Nfatc2 | 1 |
| Nfx1 | 1 |
| Nfya | 1 |
| Nif3l1 | 1 |
| Nkd2 | 1 |
| Nlgn2 | 1 |
| Nlk | 1 |
| Nln | 1 |
| Nlrp1a | 1 |
| Notch1 | 1 |
| Notch4 | 1 |
| Nova1 | 1 |
| Nox1 | 1 |
| Npat | 1 |
| Npepo | 1 |
| Npff | 1 |
| Npm1 | 1 |
| Nprl3 | 1 |
| Nqo2 | 1 |
| Nr1h2 | 1 |
| Nrcam | 1 |
| Nrp2 | 1 |
| Nsd1 | 1 |
| Nsmf | 1 |
| Nsun4 | 1 |
| Ntng1 | 1 |
| Nudt4 | 1 |
| Nup210 | 1 |
| Nxt2 | 1 |
| Nyx | 1 |
| Oas1d | 1 |
| Ocrl | 1 |
| Olr300 | 1 |
| Onecut1 | 1 |
| Onecut2 | 1 |
| Ophn1 | 1 |
| Oprm1 | 1 |
| Osgin1 | 1 |
| Otof | 1 |
| P2ry4 | 1 |
| Pafah1b1 | 1 |
| Paics | 1 |
| Paip2b | 1 |
| Pak3 | 1 |
| Pan2 | 1 |
| Panx1 | 1 |
| Papd4 | 1 |
| Papolg | 1 |
| Pard3 | 1 |
| Parvb | 1 |
| Pbx2 | 1 |
| Pcdh20 | 1 |
| Pcdh9 | 1 |
| Pcdhb11 | 1 |
| Pcdhb20 | 1 |
| Pcdhga4 | 1 |
| Pde2a | 1 |
| Pde3a | 1 |
| Pde4d | 1 |
| Pde6h | 1 |
| Pde7a | 1 |
| Pde7b | 1 |
| Pdf | 1 |
| Pdgfa | 1 |
| Pdk1 | 1 |
| Pdlim5 | 1 |
| Pdzd8 | 1 |
| Per2 | 1 |
| Per3 | 1 |
| Pex11a | 1 |
| Pex14 | 1 |
| Pfas | 1 |
| Pfkl | 1 |
| Pggt1b | 1 |
| Phf3 | 1 |
| Phip | 1 |
| Phka2 | 1 |
| Phkb | 1 |
| Phpt1 | 1 |
| Pi4ka | 1 |
| Pik3c2b | 1 |
| Pik3ca | 1 |
| Pik3cb | 1 |
| Pik3r2 | 1 |
| Pik3r3 | 1 |
| Pikfyve | 1 |
| Pip5k1b | 1 |
| Pitpnb | 1 |
| Pkd2l1 | 1 |
| Pkd2l2 | 1 |
| Pla2g4a | 1 |
| Plag1 | 1 |
| Plcb1 | 1 |
| Plcg1 | 1 |
| Plch2 | 1 |
| Plcl2 | 1 |
| Plekha1 | 1 |
| Plxna2 | 1 |
| Plxnb1 | 1 |
| Plxnc1 | 1 |
| Pml | 1 |
| Pogk | 1 |
| Poglut1 | 1 |
| Pola1 | 1 |
| Polr3c | 1 |
| Polr3f | 1 |
| Pou3f1 | 1 |
| Pou4f1 | 1 |
| Pou6f1 | 1 |
| Ppap2b | 1 |
| Ppm1a | 1 |
| Ppm1b | 1 |
| Ppm1k | 1 |
| Ppm1l | 1 |
| Ppp1r1a | 1 |
| Ppp1r2 | 1 |
| Ppp1r3b | 1 |
| Ppp2r3a | 1 |
| Prep | 1 |
| Prkaa1 | 1 |
| Prkaa2 | 1 |
| Prkar1a | 1 |
| Prkx | 1 |
| Prmt6 | 1 |
| Prmt8 | 1 |
| Prnd | 1 |
| Prokr1 | 1 |
| Prox1 | 1 |
| Prpf18 | 1 |
| Prpf3 | 1 |
| Prps1 | 1 |
| Prss53 | 1 |
| Prune2 | 1 |
| Psen1 | 1 |
| Psip1 | 1 |
| Ptges | 1 |
| Pth1r | 1 |
| Ptplad1 | 1 |
| Ptpn3 | 1 |
| Ptprj | 1 |
| Ptprz1 | 1 |
| Puf60 | 1 |
| Pvrl1 | 1 |
| Rab15 | 1 |
| Rab34 | 1 |
| Rab36 | 1 |
| Rab3ip | 1 |
| Rab40b | 1 |
| Rabl3 | 1 |
| Ralbp1 | 1 |
| Ran | 1 |
| Rangap1 | 1 |
| Rapgef4 | 1 |
| Rara | 1 |
| Rasd2 | 1 |
| Rasgef1a | 1 |
| Rasgrp3 | 1 |
| Rb1cc1 | 1 |
| Rbm10 | 1 |
| Rbm25 | 1 |
| Rbm4 | 1 |
| Rbm4b | 1 |
| Rbmx | 1 |
| Rbpj | 1 |
| Rbx1 | 1 |
| Rcn1 | 1 |
| Rcor2 | 1 |
| Rev3l | 1 |
| Rfc1 | 1 |
| Rfng | 1 |
| Rfx5 | 1 |
| RGD1309821 | 1 |
| RGD735029 | 1 |
| Rgn | 1 |
| Rgs17 | 1 |
| Rgs18 | 1 |
| Rgs4 | 1 |
| Rhbdl3 | 1 |
| Rhobtb2 | 1 |
| Rhod | 1 |
| Rhoq | 1 |
| Rims4 | 1 |
| Rin1 | 1 |
| Rin2 | 1 |
| Rnf138 | 1 |
| Rnf144b | 1 |
| Rnf38 | 1 |
| Rnmt | 1 |
| Rogdi | 1 |
| Rora | 1 |
| Rorb | 1 |
| Rp2 | 1 |
| Rpa1 | 1 |
| Rpl15 | 1 |
| Rps6ka2 | 1 |
| Rps6ka3 | 1 |
| Rps6ka5 | 1 |
| Rrbp1 | 1 |
| RT1-Db1 | 1 |
| Rtf1 | 1 |
| Rtn4 | 1 |
| Runx1 | 1 |
| Rybp | 1 |
| Ryr2 | 1 |
| S1pr1 | 1 |
| Sarnp | 1 |
| Sbno1 | 1 |
| Scamp1 | 1 |
| Scarb1 | 1 |
| Sclt1 | 1 |
| Scn3a | 1 |
| Scn4b | 1 |
| Scn9a | 1 |
| Scrn1 | 1 |
| Scube1 | 1 |
| Sdc3 | 1 |
| Sec63 | 1 |
| Sel1l | 1 |
| Sema3a | 1 |
| Sema5a | 1 |
| Sema6a | 1 |
| Senp1 | 1 |
| Senp5 | 1 |
| Senp6 | 1 |
| Sfn | 1 |
| Sfrp1 | 1 |
| Sh2b1 | 1 |
| Sh2d1a | 1 |
| Sh2d1b | 1 |
| Sh3pxd2a | 1 |
| Shank1 | 1 |
| Shank2 | 1 |
| Shank3 | 1 |
| Shb | 1 |
| Shroom4 | 1 |
| Sim1 | 1 |
| Sin3a | 1 |
| Sirt4 | 1 |
| Six4 | 1 |
| Ska1 | 1 |
| Skp2 | 1 |
| Slc12a3 | 1 |
| Slc12a5 | 1 |
| Slc12a8 | 1 |
| Slc17a6 | 1 |
| Slc19a2 | 1 |
| Slc1a5 | 1 |
| Slc22a23 | 1 |
| Slc23a1 | 1 |
| Slc23a2 | 1 |
| Slc25a22 | 1 |
| Slc25a25 | 1 |
| Slc25a36 | 1 |
| Slc28a3 | 1 |
| Slc30a6 | 1 |
| Slc35b3 | 1 |
| Slc35d1 | 1 |
| Slc35f6 | 1 |
| Slc38a4 | 1 |
| Slc44a2 | 1 |
| Slc46a1 | 1 |
| Slc4a11 | 1 |
| Slc5a12 | 1 |
| Slc5a7 | 1 |
| Slc6a6 | 1 |
| Slc8a1 | 1 |
| Slc9a8 | 1 |
| Slfn14 | 1 |
| Slit2 | 1 |
| Slitrk3 | 1 |
| Smad3 | 1 |
| Smad9 | 1 |
| Smap2 | 1 |
| Snap23 | 1 |
| Snrk | 1 |
| Snx19 | 1 |
| Snx2 | 1 |
| Snx24 | 1 |
| Snx33 | 1 |
| Snx5 | 1 |
| Socs7 | 1 |
| Sorcs1 | 1 |
| Sorl1 | 1 |
| Sort1 | 1 |
| Sox17 | 1 |
| Spag16 | 1 |
| Sparcl1 | 1 |
| Specc1 | 1 |
| Spib | 1 |
| Spire1 | 1 |
| Spred1 | 1 |
| Spsb2 | 1 |
| Sra1 | 1 |
| Srd5a2 | 1 |
| Srebf1 | 1 |
| Srek1 | 1 |
| Srgap1 | 1 |
| Srgap3 | 1 |
| Srpk1 | 1 |
| Srsf10 | 1 |
| Srsf9 | 1 |
| Srxn1 | 1 |
| Ss18 | 1 |
| Ssh2 | 1 |
| Sstr4 | 1 |
| St3gal1 | 1 |
| St3gal6 | 1 |
| Stard13 | 1 |
| Stk10 | 1 |
| Stk38l | 1 |
| Strbp | 1 |
| Stx17 | 1 |
| Stx3 | 1 |
| Stx4 | 1 |
| Stx6 | 1 |
| Stxbp6 | 1 |
| Supt20 | 1 |
| Suv39h2 | 1 |
| Syk | 1 |
| Syt13 | 1 |
| Tacc3 | 1 |
| Tacr1 | 1 |
| Taf12 | 1 |
| Taf1a | 1 |
| Tapt1 | 1 |
| Tbx10 | 1 |
| Tbx19 | 1 |
| Tbx4 | 1 |
| Tcerg1 | 1 |
| Tcf20 | 1 |
| Tcf7l2 | 1 |
| Tcl1a | 1 |
| Tead1 | 1 |
| Terf1 | 1 |
| Tet2 | 1 |
| Tex261 | 1 |
| Tfb2m | 1 |
| Tfe3 | 1 |
| Tfpi | 1 |
| Tgfbr1 | 1 |
| Tgm2 | 1 |
| Tgoln2 | 1 |
| Tgs1 | 1 |
| Them4 | 1 |
| Thrb | 1 |
| Tlk1 | 1 |
| Tln2 | 1 |
| Tm2d2 | 1 |
| Tmed1 | 1 |
| Tmeff2 | 1 |
| Tmem119 | 1 |
| Tmem204 | 1 |
| Tmem56 | 1 |
| Tmprss11e | 1 |
| Tmprss13 | 1 |
| Tmsb4x | 1 |
| Tmx1 | 1 |
| Tnfaip1 | 1 |
| Tnfrsf8 | 1 |
| Tnfsf9 | 1 |
| Tnk1 | 1 |
| Tnks | 1 |
| Tnpo2 | 1 |
| Tnrc6b | 1 |
| Tns1 | 1 |
| Tp53bp1 | 1 |
| Traf3 | 1 |
| Trak2 | 1 |
| Trappc3 | 1 |
| Trerf1 | 1 |
| Trhde | 1 |
| Trim2 | 1 |
| Trim26 | 1 |
| Trim39 | 1 |
| Trim41 | 1 |
| Trim66 | 1 |
| Trpm7 | 1 |
| Trpv1 | 1 |
| Tspy1 | 1 |
| Tspyl5 | 1 |
| Ttbk2 | 1 |
| Ttl | 1 |
| Ttll7 | 1 |
| Tubgcp6 | 1 |
| Twf1 | 1 |
| U2af1l4 | 1 |
| Ube2m | 1 |
| Ube2w | 1 |
| Ube4a | 1 |
| Ubp1 | 1 |
| Ubr7 | 1 |
| Ugdh | 1 |
| Ulk4 | 1 |
| Unc5c | 1 |
| Ung | 1 |
| Usf2 | 1 |
| Usp13 | 1 |
| Usp19 | 1 |
| Usp24 | 1 |
| Usp27x | 1 |
| Usp33 | 1 |
| Usp38 | 1 |
| Usp46 | 1 |
| Usp47 | 1 |
| Usp7 | 1 |
| Utrn | 1 |
| Vangl1 | 1 |
| Vangl2 | 1 |
| Vav3 | 1 |
| Vcam1 | 1 |
| Vcl | 1 |
| Vegfb | 1 |
| Vgll3 | 1 |
| Vps4b | 1 |
| Vps54 | 1 |
| Vsx1 | 1 |
| Wapal | 1 |
| Wdr44 | 1 |
| Wdr45b | 1 |
| Wee1 | 1 |
| Wfikkn2 | 1 |
| Whsc1 | 1 |
| Whsc1l1 | 1 |
| Wnk3 | 1 |
| Wnt6 | 1 |
| Wwox | 1 |
| Wwp1 | 1 |
| XAF1 | 1 |
| Xcr1 | 1 |
| Xpo7 | 1 |
| Xrn2 | 1 |
| Xylt1 | 1 |
| Yaf2 | 1 |
| Yeats4 | 1 |
| Yipf5 | 1 |
| Ywhab | 1 |
| Zbtb10 | 1 |
| Zbtb40 | 1 |
| Zbtb7a | 1 |
| Zc3h3 | 1 |
| Zfhx4 | 1 |
| Zfp14 | 1 |
| Zfp148 | 1 |
| Zfp167 | 1 |
| Zfp187 | 1 |
| Zfp191 | 1 |
| Zfp202 | 1 |
| Zfp26 | 1 |
| Zfp260 | 1 |
| Zfp286a | 1 |
| Zfp335 | 1 |
| Zfp36 | 1 |
| Zfp386 | 1 |
| Zfp438 | 1 |
| Zfp445 | 1 |
| Zfp462 | 1 |
| Zfp689 | 1 |
| Zfp780b | 1 |
| Zfp819 | 1 |
| Zfp867 | 1 |
| Zfpm2 | 1 |
| Zfyve9 | 1 |
| Zhx1 | 1 |
| Zic2 | 1 |
| Zmiz2 | 1 |
| Zmym4 | 1 |
| Zranb2 | 1 |
